# Global burden and trend of stroke attributable to metabolic risks among young adults (20–39 years old) from 1990 to 2021

**DOI:** 10.3389/fcvm.2025.1561052

**Published:** 2025-06-02

**Authors:** Qingguo Liu, Jiao Su, Yuanhao Liang, Xiaofeng He

**Affiliations:** ^1^ Department of Neurosurgery, Heping Hospital Affiliated to Changzhi Medical College, Changzhi, China; ^2^ Department of Biochemistry, Changzhi Medical College, Changzhi, China; ^3^ Clinical Experimental Center, Jiangmen Key Laboratory of Clinical Biobanks and Translational Research, Jiangmen Central Hospital, Jiangmen, China; ^4^ Institute of Evidence-Based Medicine, Heping Hospital Affiliated to Changzhi Medical College, Changzhi, China

**Keywords:** global burden of disease study, stroke, young adults, metabolic risk factors, geographical distribution, long-term trend

## Abstract

**Objectives:**

Stroke is increasingly affecting young adults, with metabolic-risk factors playing a critical role in this trend. This study aims to assess the global burden and trends of stroke and its subtypes attributable to metabolic-risks in young adults from 1990 to 2021.

**Methods:**

Data from the Global Burden of Disease Study (GBD) 2021 were analyzed to assess the disability-adjusted life years (DALYs) attributed to metabolic-risks for stroke and its subtypes in young adults across 204 countries and territories. Estimated annual percentage changes in the age-standardized DALYs rate (ASDR) of stroke, by age, sex, socio-demographic index (SDI), and subtype, were calculated to quantify the temporal trends.

**Results:**

In 2021, metabolic risk factors were responsible for approximately 3,960,349 stroke-DALYs in young adults globally, accounting for 45.44% of the total stroke burden in this group. High systolic blood pressure was the leading contributor (35.43%), followed by high LDL cholesterol (9.13%), high BMI (7.26%), kidney dysfunction (5.47%), and high fasting plasma glucose (2.42%). From 1990 to 2021, the absolute number of stroke-related DALYs attributable to metabolic-risks increased by 22.23%, while the ASDR decreased by 0.78% annually. Regional disparities were evident, with East Asia reporting the largest number of stroke-DALYs attributable to metabolic-risks and Southeast Asia exhibiting the highest ASDR. Notably, the proportion of stroke-DALYs attributable to metabolic-risks showed a positive association with SDI and increased across all regions during the study period. The most notable increases were observed in Eastern Europe. By stroke subtype, metabolic risk factors contributed to 1,147,521 DALYs from ischemic stroke, 2,267,874 from intracerebral hemorrhage, and 544,954 from subarachnoid hemorrhage in 2021. The ASDR of all subtypes declined from 1990 to 2021, with the steepest decline for subarachnoid hemorrhage (EAPC = –1.37%). However, ASDR increased in specific regions, notably Sub-Saharan Africa for ischemic stroke and the Caribbean and Oceania for intracerebral hemorrhage and subarachnoid hemorrhage.

**Conclusions:**

Despite a decline in ASDR, the absolute burden of stroke attributable to metabolic risks among young adults has increased globally, with significant regional and national disparities. Targeted prevention strategies addressing metabolic risk factors are urgently needed, particularly in high-burden regions.

## 1 Introduction

Traditionally, stroke has been considered a condition that primarily affects older adults; However, there is a concerning shift, with an increasing number of stroke cases being reported among younger populations, largely due to the rising prevalence of metabolic diseases (1–3). When stroke occurs in young adults, it often leads to significant long-term morbidity, disability, and a diminished quality of life (4). Stroke survivors in this age group may experience challenges such as cognitive impairments, loss of independence, and difficulties in pursuing education or career aspirations (5, 6). Furthermore, the growing incidence of stroke in younger individuals places additional strain on healthcare systems, as these patients often require prolonged medical care, rehabilitation, and long-term support services (7).

Understanding the trends and burden of stroke and its subtypes among young adults is essential for developing evidence-based prevention and treatment strategies tailored specifically to this age group. The increasing recognition of metabolic risk factors—such as obesity, hypertension, diabetes, and dyslipidemia—in young adults highlights the urgent need to address these underlying causes of stroke (8, 9). Historically, these conditions were more common in older populations, but they are now emerging at younger ages, significantly contributing to the rising burden of stroke in this group. Early identification of these metabolic risks provides an opportunity for timely interventions that could prevent or delay the onset of stroke in later life (10).

By analyzing stroke trends attributable to metabolic risks in young adults, public health strategies can be refined to allocate resources more effectively and provide tailored prevention and management guidelines. A key advantage of this approach is the identification of modifiable risk factors that can be addressed early. Lifestyle changes, such as weight management, regular physical activity, and improved nutrition, have been shown to reduce the risk of both metabolic diseases and stroke (11, 12). Early intervention in adolescence and young adulthood can mitigate future stroke risks and help reduce the long-term societal burden of the disease (13). Public health campaigns can also be better designed to raise awareness about the link between metabolic conditions and stroke, while health policies can prioritize early screening, prevention, and intervention, particularly in regions where metabolic risk factors are rising rapidly (14).

In conclusion, evaluating the burden and trends of stroke attributable to metabolic risks in young adults is critical for the development of effective, age-specific prevention and intervention strategies. Given the increasing prevalence of metabolic risk factors in this age group, addressing these issues is essential not only for improving individual health outcomes but also for reducing the broader societal impact of stroke (15).

## 2 Methods

### 2.1 Global burden of disease study

The Global Burden of Disease Study (GBD) is a comprehensive research initiative that assesses the impact of diseases, injuries, and risk factors on global health across countries, regions, and over time (16). It was launched by the World Health Organization (WHO) and the Institute for Health Metrics and Evaluation (IHME) in collaboration with over 1,000 researchers globally. The GBD provides a detailed understanding of the distribution and trends of diseases and injuries, as well as their associated risk factors, by calculating key health metrics such as mortality, morbidity, and disability-adjusted life years (DALYs) (17, 18).

The GBD study integrates data from diverse sources, including vital registration systems, surveys, and health studies, to produce estimates for nearly all countries and territories. These estimates are updated annually, offering insights into health priorities and helping to guide global health policy and resource allocation (19). One of the major strengths of the GBD is its ability to analyze health trends over time, identify emerging health threats, and highlight regional disparities in health outcomes (17, 18).

### 2.2 Data source and collection

Stroke, as defined by the World Health Organization (WHO), is characterized by rapidly developing clinical signs of focal disturbance in cerebral function, lasting more than 24 h or leading to death (20). In the GBD 2021, the burden of stroke was modeled using verbal autopsy and vital registration data (17). Stroke was classified according to the International Classification of Diseases (ICD) codes: G45–G46.8, I60–I63.9, I65–I66.9, I67.0–I67.3, I67.5–I67.6, I68.1–I68.2, and I69.0–I69.3 based on ICD-10, as well as 430–435.9, 437.0–437.2, and 437.5–437.8 under ICD-9 (20). Furthermore, data were collected for specific stroke subtypes, including ischemic stroke (ICD-10: G45–G46.8, I63–I63.9, I65–I66.9, I67.2–I67.848, I69.3–I69.4; ICD-9: 433–435.9, 437.0–437.2, 437.4–437.9), intracerebral hemorrhage (ICD-10: I61–I62, I62.9, I69.0–I69.298; ICD-9: 431, 431.1–432.9), and subarachnoid hemorrhage (ICD-10: I60–I60.9, I67.0–I67.1; ICD-9: 430–430.9, 431.0, 437.3). Comprehensive details regarding the stroke burden estimation methodology can be found in the GBD 2021 methods appendices (https://www.healthdata.org/gbd/methods-appendices-2021/stroke).

To analyze the attributable burden of stroke from metabolic risk factors in the GBD 2021, population attributable fractions (PAFs) of DALYs were calculated. Notably, the Global Burden of Disease (GBD) framework estimates the burden of stroke attributable to metabolic risks using the Comparative Risk Assessment (CRA) methodology (18, 21). Rather than categorizing individuals as simply “having” or “not having” a metabolic risk factor, this approach quantifies the proportion of stroke burden attributable to each risk factor by comparing observed exposure distributions with the theoretical minimum risk exposure level (TMREL). This methodology provides a nuanced, population-level assessment of risk factor contributions, allowing for a more comprehensive understanding of their impact without implying direct causality at the individual level. This process involved several steps: (1) identifying risk-outcome pairs, (2) estimating relative risk for each exposure, (3) determining exposure levels by age, sex, location, and year, (4) identifying the TMREL and counterfactual exposure, (5) estimating the attributable burden and PAFs, and (6) calculating DALYs attributable to various combinations of risk factors (18). The metabolic risk factors considered in this analysis included high body-mass index (BMI), high fasting plasma glucose, high systolic blood pressure, high low-density lipoprotein (LDL) cholesterol, and kidney dysfunction (Supplementary Table S1) (20). In this study, the denominator is the total number of stroke-related DALYs in young adults (20–39 years old). The attributable proportion is derived by dividing the stroke DALYs attributable to metabolic risks by the total stroke DALYs in that group.

For this study, data on the DALYs attributable to metabolic risk factors for stroke in individuals aged 20–39 years were extracted from the GBD 2021 using the Global Health Data Exchange (GHDx) query tool (http://ghdx.healthdata.org/gbd-results-tool). DALYs represent a combined measure of health loss from both fatal and non-fatal outcomes, calculated as the sum of years of life lost (YLLs) and years lived with disability (YLDs) (22).

### 2.3 Socio-demographic index (SDI)

The SDI is a composite indicator used to assess development conditions and is closely linked to health outcomes (17). It is calculated as the geometric mean of three components: (1) lag-distributed income per capita, (2) total fertility rate among women under 25 years of age, and (3) average education level of individuals aged 15 years and older. The SDI has values ranging from 0 to 1, with higher values indicating more developed conditions. The SDI is categorized into five quintiles for 204 countries and territories: low, low-middle, middle, high-middle, and high (Supplementary Table S2). Additionally, all 204 countries and territories were categorized into 21 GBD regions based on epidemiological similarities and geographic proximity (Supplementary Table S3).

### 2.4 Statistical analysis

To examine the burden of stroke and its subtypes attributable to metabolic risk factors among young adults, we calculated the age-standardized DALYs rate (ASDR) per 100,000 individuals using the following formula:

$$\mathrm{ASDR}\;=\;\frac{\sum_{i\;=\;1}^{A}\;a_{i}w_{i}}{\sum_{i\;=\;1}^{A}\;w_{i}}\;\times 100,000$$

In this formula, $a_{i}$ signifies the age-specific rate of DALYs within the $i^{th}$ age subgroup. $w_{i}$ represents the standard population size for that age group, based on the GBD standard Population Estimates (1950–2021) (23). The summation (∑) accounts for all age groups included in the analysis. This method ensures that differences in population age structures do not distort comparisons across regions or time periods. By weighting age-specific DALY rates according to a standard population, ASDR allows for a more accurate comparison of stroke burden between countries and over time.

Additionally, assuming a linear relationship between the natural logarithm of ASDR and time, the model is expressed as y=α+βx+ε, where y=ln(ASDR), x=calendaryear, and *ε* = the error term. The Estimated Annual Percent Change (EAPC) was computed using the formula: 100×(exp(β)−1) (24). The 95% confidence intervals (CIs) was derived from the linear regression model. If both the EAPC and the lower bound of its 95% CI were positive, the ASDR was considered to show an increasing trend. Conversely, if the EAPC and the upper bound of the 95% CI were negative, the ASDR was considered to show a decreasing trend.

Moreover. to assess the relationship between stroke DALYs attributable to metabolic risk factors and SDI, Spearman correlation analysis was conducted to calculate the correlation coefficient (*ρ*) and *p*-values. Statistical analysis was performed using R software, version 4.1.0 (R Foundation for Statistical Computing). A two-tailed *p*-value of < 0.05 was considered statistically significant.

## 3 Results

### 3.1 Global DALYs of stroke attributable to metabolic risks

In 2021, metabolic risk factors were responsible for approximately 3,960,349 stroke-DALYs in young adults globally, accounting for 45.44% of the total stroke DALYs in this group (Table 1, Supplementary Table S4). Among these, high systolic blood pressure was the leading contributor, accounting for 35.43% of stroke DALYs. Other significant contributors included high LDL cholesterol (9.13%), high BMI (7.26%), kidney dysfunction (5.47%), and high fasting plasma glucose (2.42%) (Supplementary Table S3). Moreover, the proportion of stroke DALYs attributable to metabolic risks was higher in male young adults compared to female young adults, except for high LDL cholesterol and high BMI (Supplementary Table S4).

**Table 1:** Global disability-adjusted life years (DALYs) of stroke attributable to metabolic risk factors among young adults in 1990 and 2021, and estimated annual percentage changes from 1990 to 2021.

Characteristics	1990		2021		1990–2021	
	Number of DALYs	Age-standardized rate per 100,000 population (95% UI)	Number of DALYs	Age-standardized rate per 100,000 population (95% UI)	Relative change (%)	Estimated annual percentage changes (95% CI)
Global	3,240,105	210.91 (210.68 to 211.14)	3,960,349	168.46 (168.3 to 168.63)	22.23	−0.78 (−0.87 to −0.69)
Sex						
Female	1,331,292	123.29 (123.09 to 123.49)	1,434,322	175.26 (174.97 to 175.56)	7.74	−1.24 (−1.32 to −1.17)
Male	1,908,813	212.73 (212.47 to 213)	2,526,026	245.62 (245.27 to 245.97)	32.33	−0.48 (−0.6 to −0.37)
Cause						
Ischemic stroke	877,865	56.74 (56.86 to 56.62)	1,147,521	48.81 (48.9 to 48.72)	30.72	−0.55 (−0.59 to −0.51)
Intracerebral hemorrhage	1,836,282	119.92 (120.09 to 119.75)	2,267,874	96.47 (96.6 to 96.34)	23.5	−0.74 (−0.89 to −0.59)
Subarachnoid hemorrhage	525,957	34.25 (34.34 to 34.15)	544,954	23.18 (23.24 to 23.12)	3.61	−1.37 (−1.43 to −1.32)
Socio-demographic index						
High	362,647	127.79 (127.37 to 128.2)	257,341	82.89 (82.57 to 83.22)	−29.04	−1.4 (−1.6 to −1.2)
High-middle	745,242	218.6 (218.1 to 219.09)	678,109	167.16 (166.76 to 167.56)	−9.01	−1.09 (−1.28 to −0.91)
Middle	1,175,103	236.12 (235.69 to 236.55)	1,389,321	181.26 (180.95 to 181.56)	18.23	−0.86 (−0.96 to −0.76)
Low-middle	710,820	240.49 (239.92 to 241.05)	1,120,486	192.87 (192.52 to 193.23)	57.63	−0.69 (−0.78 to −0.59)
Low	242,727	209.18 (208.34 to 210.02)	511,404	180.13 (179.64 to 180.63)	110.69	−0.56 (−0.63 to −0.48)
GBD regions						
High-income Asia Pacific	83,143	160.21 (159.12 to 161.3)	29,070	64.2 (63.46 to 64.94)	−65.04	−3.61 (−3.93 to −3.28)
Central Asia	57,429	286.62 (284.26 to 289)	61,228	196.22 (194.67 to 197.79)	6.62	−1.71 (−2.01 to −1.4)
East Asia	796,107	202.68 (202.24 to 203.13)	839,079	186.62 (186.22 to 187.02)	5.4	−0.35 (−0.49 to −0.21)
South Asia	503,615	174.5 (174.01 to 174.98)	793,596	136.35 (136.05 to 136.65)	57.58	−0.81 (−0.96 to −0.66)
Southeast Asia	490,123	375.06 (374 to 376.12)	698,404	318.53 (317.78 to 319.28)	42.5	−0.38 (−0.52 to −0.24)
Australasia	4,402	67.77 (65.78 to 69.8)	3,587	39.85 (38.56 to 41.18)	−18.51	−2.11 (−2.28 to −1.94)
Caribbean	21,021	213.56 (210.66 to 216.49)	30,015	211.98 (209.58 to 214.39)	42.79	0.5 (0.24 to 0.76)
Central Europe	93,103	238.3 (236.77 to 239.84)	36,341	112.27 (111.11 to 113.44)	−60.97	−2.7 (−2.82 to −2.59)
Eastern Europe	167,731	226.63 (225.54 to 227.72)	152,438	229.75 (228.58 to 230.92)	−9.12	−0.46 (−0.94 to 0.02)
Western Europe	136,190	118.94 (118.31 to 119.57)	47,011	42.31 (41.92 to 42.69)	−65.48	−3.54 (−3.7 to −3.37)
Andean Latin America	14,468	142.97 (140.63 to 145.33)	23,547	113.04 (111.59 to 114.49)	62.75	−0.53 (−0.85 to −0.21)
Central Latin America	63,289	143.59 (142.47 to 144.72)	77,570	100.35 (99.65 to 101.06)	22.56	−1.21 (−1.57 to −0.86)
Southern Latin America	23,356	167.32 (165.18 to 169.48)	19,031	91.97 (90.67 to 93.28)	−18.52	−1.73 (−1.88 to −1.59)
Tropical Latin America	138,647	317.41 (315.74 to 319.1)	91,669	124.92 (124.12 to 125.74)	−33.88	−3.27 (−3.49 to −3.04)
North Africa and Middle East	283,360	328.38 (327.17 to 329.6)	419,983	206 (205.38 to 206.63)	48.22	−1.53 (−1.56 to −1.49)
High-income North America	87,842	92.02 (91.41 to 92.63)	78,705	77.96 (77.41 to 78.5)	−10.4	−0.51 (−0.69 to −0.33)
Oceania	4,912	283.82 (275.86 to 291.95)	12,315	306.01 (300.61 to 311.48)	150.71	0.26 (0.1 to 0.43)
Central Sub-Saharan Africa	28,056	218.6 (216.03 to 221.2)	52,577	153.65 (152.33 to 154.98)	87.4	−1.31 (−1.39 to −1.24)
Eastern Sub-Saharan Africa	95,924	225.32 (223.88 to 226.76)	205,089	189.16 (188.34 to 189.99)	113.8	−0.69 (−0.74 to −0.63)
Southern Sub-Saharan Africa	49,250	357.48 (354.3 to 360.67)	53,288	199.17 (197.48 to 200.87)	8.2	−2.09 (−2.93 to −1.24)
Western Sub-Saharan Africa	98,136	219.8 (218.42 to 221.19)	235,804	197.95 (197.15 to 198.76)	140.28	−0.23 (−0.36 to −0.11)

The ASDR of stroke attributed to metabolic risks decreased from 210.91 (95% UI, 210.68 to 211.14) per 100,000 population in 1990 to 168.46 (95% UI, 168.3 to 168.63) per 100,000 population in 2021, reflecting an average annual decline of 0.78% (95% CI, −0.87 to −0.69) (Table 1). Despite this decrease in ASDR, the global absolute number of stroke-related DALYs attributable metabolic risks increased by 22.23% from 1990 to 2021. Additionally, the proportion of DALYs attributable to metabolic risks rose from 35.15% in 1990 to 45.44% in 2021 (Table 1, Supplementary Table S4).

### 3.2 Regional DALYs of stroke attributable to metabolic risks

**Figure 1:**
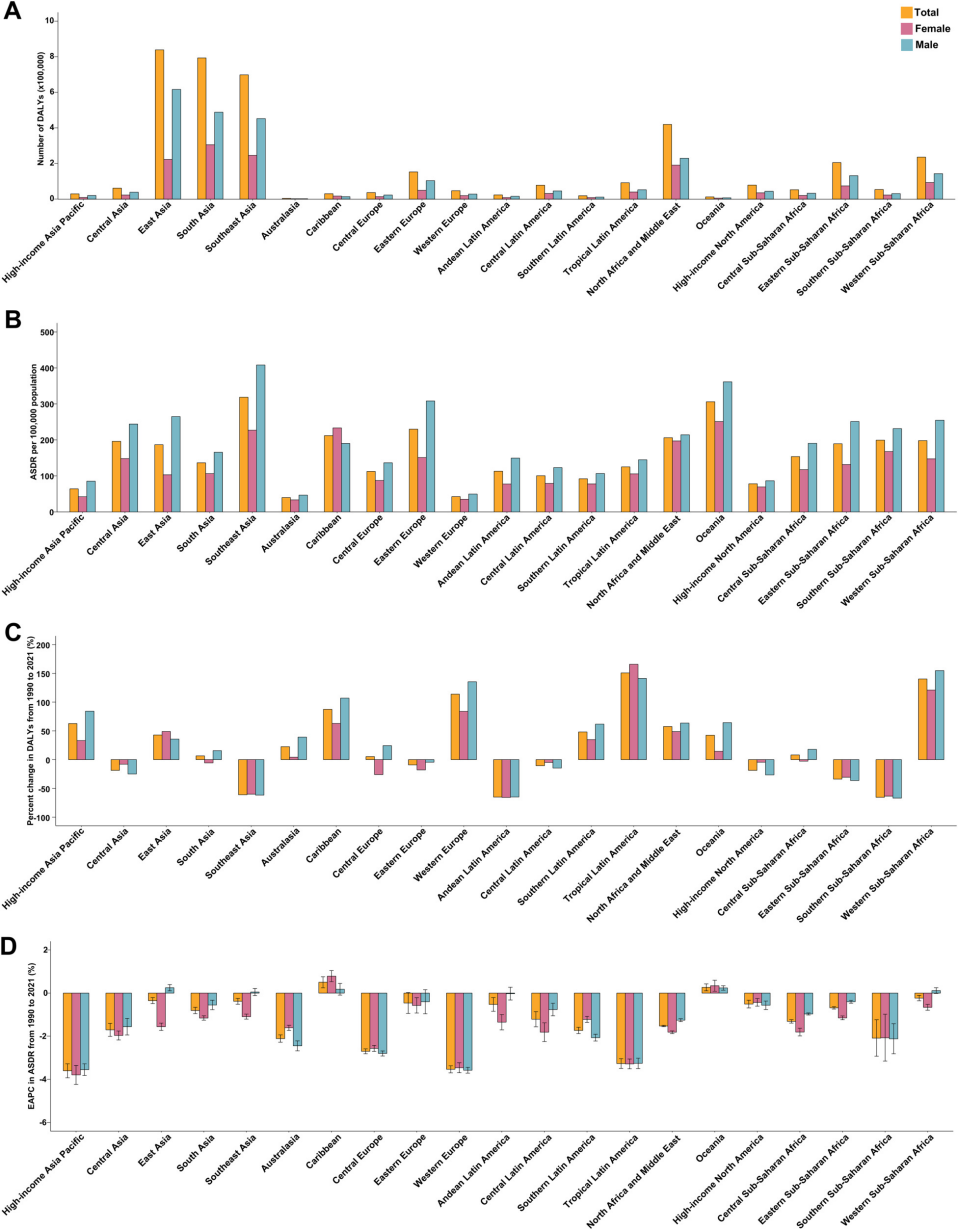
Disability-Adjusted Life Years (DALYs) and age-standardized DALYs rate (ASDR) of stroke among young adults in 2021, as well as their change from 1990 to 2021, across 21 GBD regions, by sex. **(A)** DALYs of stroke in young adults in 2021. **(B)** ASDR of stroke in young adults in 2021. **(C)** Relative change in DALYs of stroke among young adults between 1990 and 2021. **(D)** Estimated annual percentage change (EAPC) of ASDR for stroke in young adults from 1990 to 2021. GBD, Global Burden of Disease Study.

**Figure 2:**
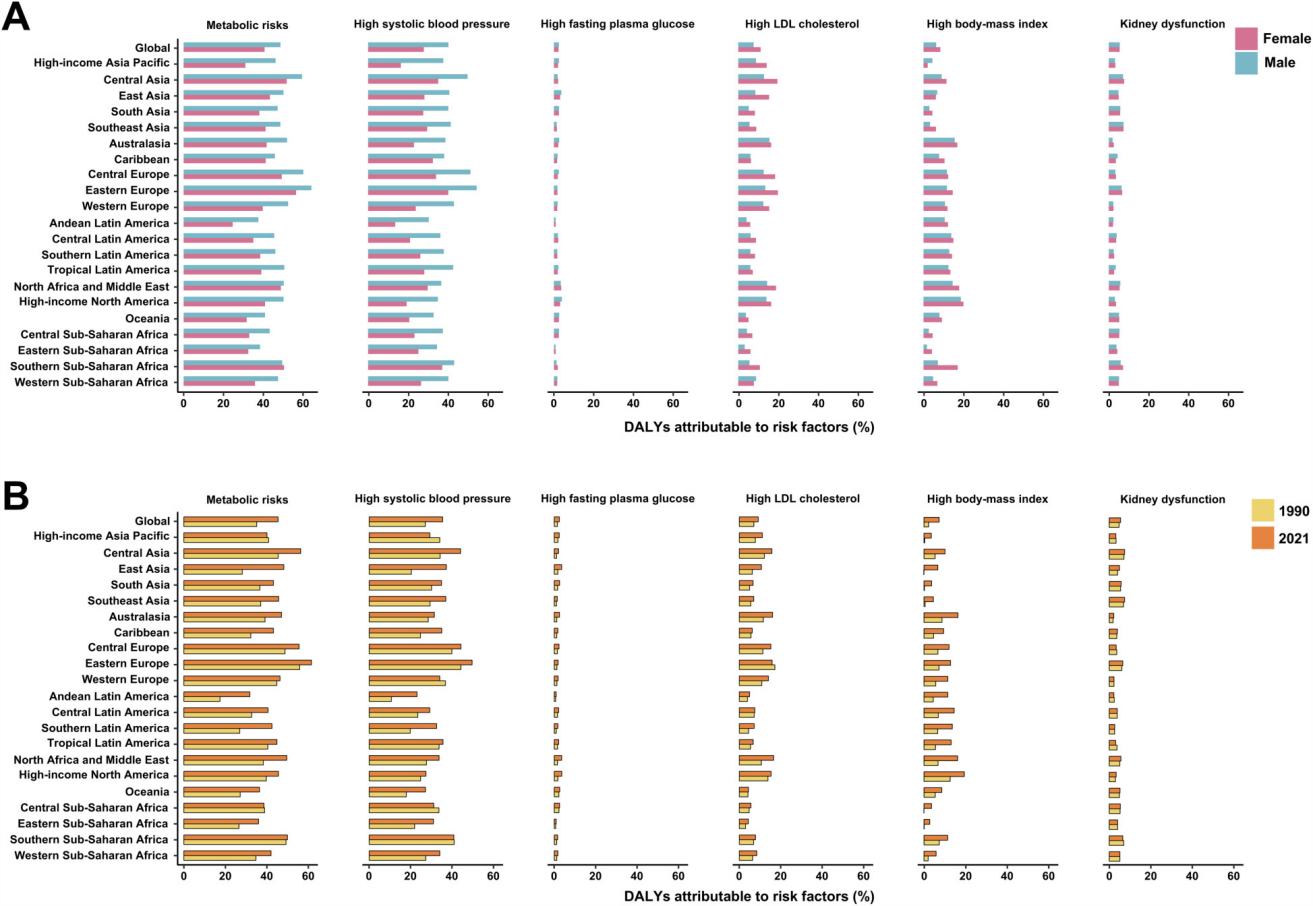
Proportion of disability-adjusted life-years (DALYs) attributable to metabolic risk factors for stroke among young adults by sex and year, globally and for 21 GBD regions. **(A)** The fractions of DALYs due to stroke attributable to metabolic risk factors for female and male young adults in 2021. **(B)** The fractions of DALYs due to stroke attributable to metabolic risk factors for both sexes combined in 1990 and 2021. GBD, Global Burden of Disease Study.

In 2021, East Asia had the largest number of stroke-related DALYs attributable to metabolic risks, while Southeast Asia had the highest ASDR for stroke (Figures 1A,B). However, the highest proportion of stroke-DALYs attributable to metabolic risks was observed in Eastern Europe (Figure 2A). The proportion of stroke-DALYs attributable to metabolic risks was higher in male young adults compared to female young adults across 21 GBD regions, except for Southern Sub-Saharan Africa (Figure 2A). Specifically, the highest proportion of stroke-DALYs attributable to high systolic blood pressure was found in Eastern Europe (49.59%), high fasting plasma glucose in High-income North America (3.59%), high LDL cholesterol in North Africa and the Middle East (16.52%), high BMI in High-income North America (19.43%), and kidney dysfunction in Southeast Asia (7.42%) (Figure 2A).

Regionally, 13 of the 21 GBD regions experienced an increase in the absolute number of stroke-related DALYs attributable to metabolic risks from 1990 to 2021, with the largest increase observed in Tropical Latin America (Figure 1C). In contrast, an increase in the ASDR of stroke attributable to metabolic risks during the same period was only observed in the Caribbean and Oceania (Figure 1D). Notably, the proportion of stroke-DALYs attributable to metabolic risks universally increased across all 21 GBD regions between 1990 and 2021 (Figure 2B).

### 3.3 National and territorial DALYs of stroke attributable to metabolic risks

**Figure 3:**
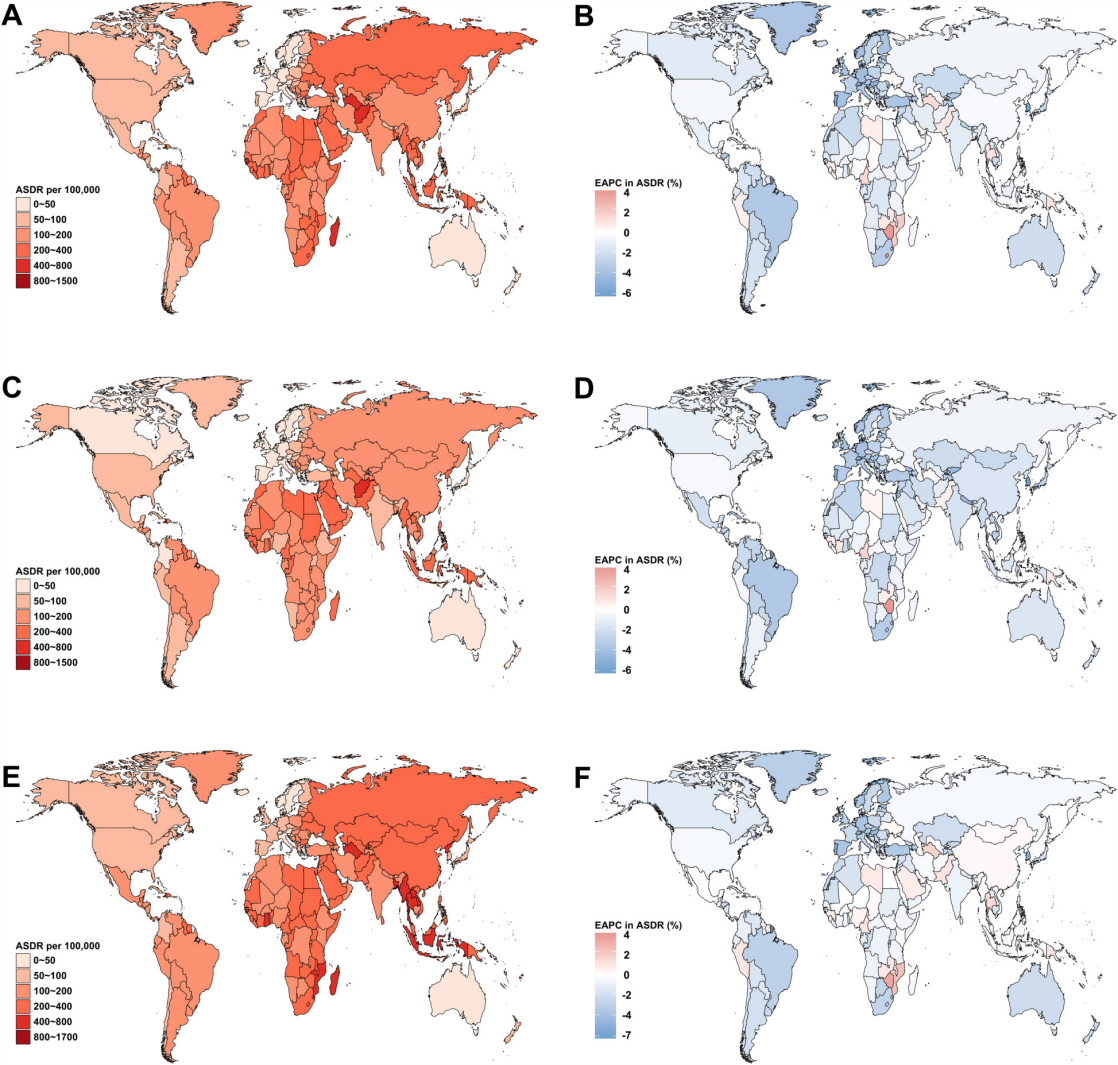
The global age-standardized DALYs rate (ASDR) of stroke in young adults across 204 countries and territories in 2021, and their estimated annual percentage changes (EAPC) from 1990 to 2021, by sex. The ASDR **(A)** and its EAPC **(B)** for both sexes combined. ASDR **(C)** and its EAPC **(D)** for female. ASDR **(E)** and its EAPC **(F)** for male. DALYs, disability-adjusted life-years.

**Figure 4:**
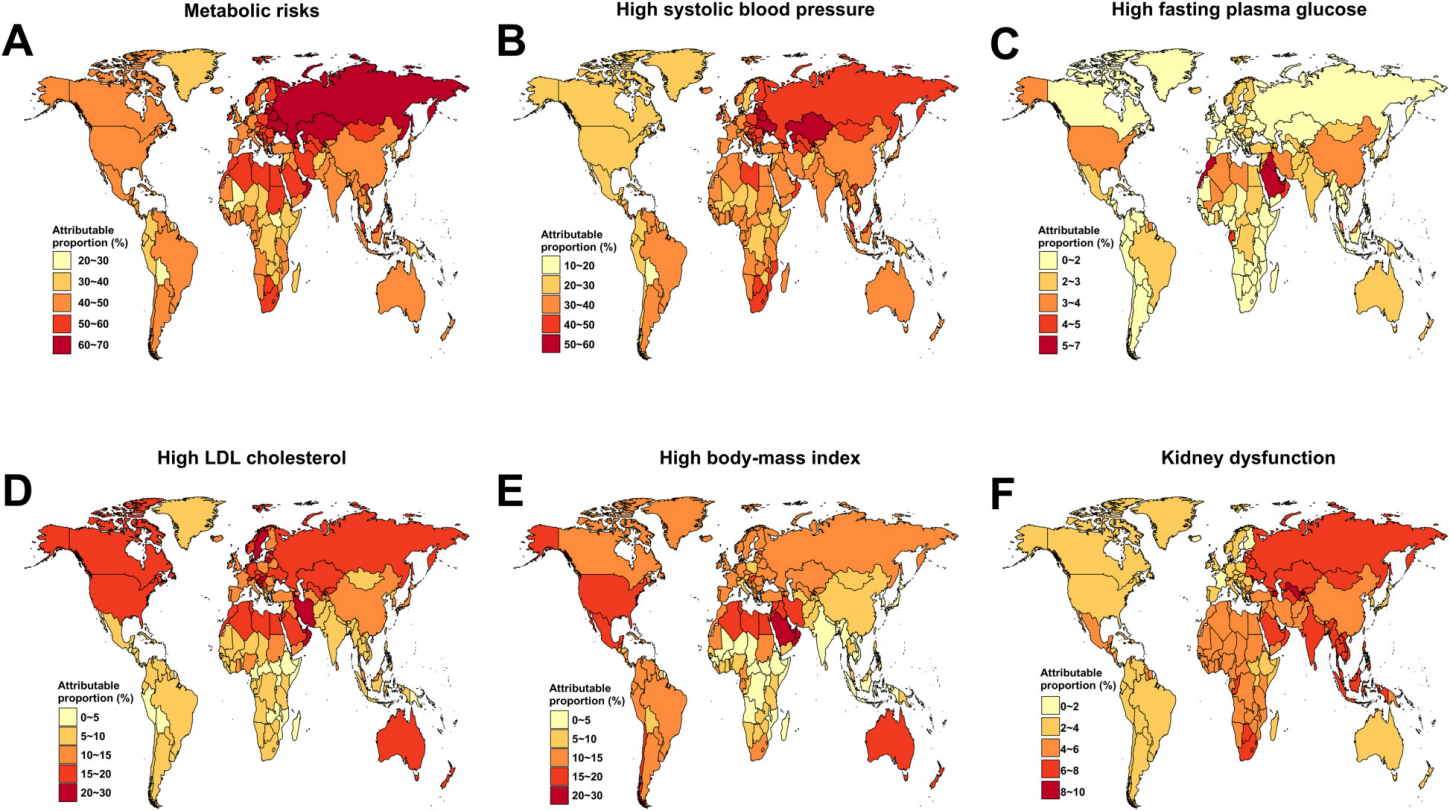
Proportion of disability-adjusted life-years (DALYs) attributable to metabolic risk factors for stroke among young adults across 204 countries and territories, by specific risk factors, 2021. **(A)** Stroke attributable to overall metabolic risk. **(B)** Stroke attributable to high systolic blood pressure. **(C)** Stroke attributable to high fasting plasma glucose. **(D)** Stroke attributable to high LDL cholesterol. **(E)** Stroke attributable to high body-mass index. **(F)** Stroke attributable to kidney dysfunction.

At the national level, in 2021, Nauru had the highest ASDR for stroke DALYs attributable to metabolic risks in both female and male young adults (Figures 3A,C,E, and Supplementary Table S5). Belarus reported the highest proportion of stroke-DALYs attributable to metabolic risks (66.31%) (Figure 4A), and the highest proportion of stroke-DALYs attributable to high systolic blood pressure (56.86%) (Figure 4B). Saudi Arabia had the highest proportion of stroke-DALYs attributable to high fasting plasma glucose (6.89%) (Figure 4C), while Kuwait reported the highest proportion of stroke-DALYs attributable to high LDL cholesterol (25.99%) (Figure 4D). The United Arab Emirates had the highest proportion of stroke-DALYs attributable to high BMI (26.42%) (Figure 4E), and Seychelles reported the highest proportion of stroke-DALYs attributable to kidney dysfunction (8.50%) (Figure 4F).

From 1990 to 2021, 27 countries and territories experienced an increase in the ASDR of stroke attributable to metabolic risks for both sexes combined, with the fastest increases observed in Zimbabwe (EAPC = 3.87) and Lesotho (EAPC = 3.69) (Figure 3B). Among female young adults, 22 countries and territories saw an increase in the ASDR of stroke attributable to metabolic risks, with the largest increases in Zimbabwe (EAPC = 3.90) and Lesotho (EAPC = 3.82) (Figure 3D). In contrast, 34 countries and territories experienced an increase in the ASDR of stroke attributable to metabolic risks in male young adults during the same period, with the fastest increases again observed in Zimbabwe (EAPC = 3.93) and Lesotho (EAPC = 3.35) (Figure 3F).

### 3.4 DALYs of stroke attributable to metabolic risks, by SDI

**Figure 5:**
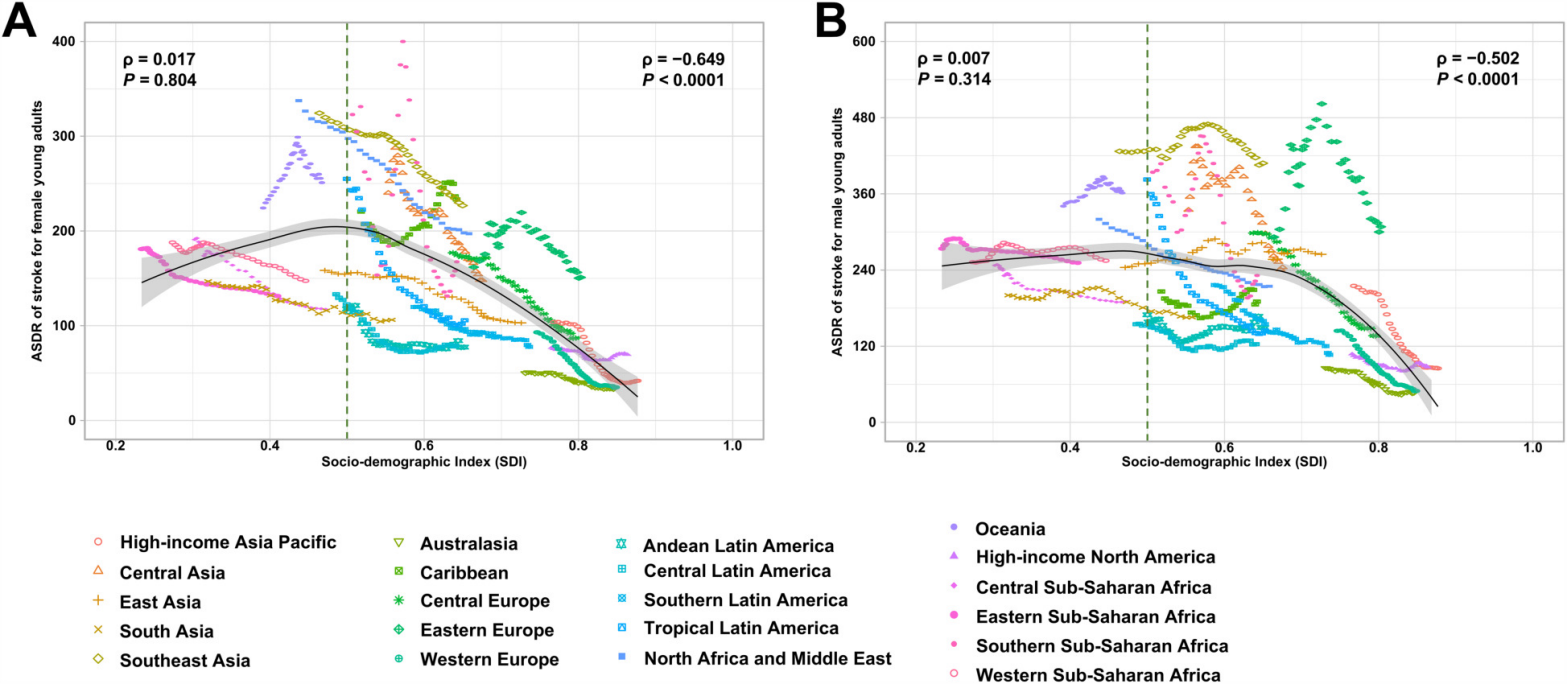
Age-standardized DALYs rate (ASDR) of stroke attributable to metabolic risk factors in **(A)** female and **(B)** male young adults, globally and for 21 GBD regions, by SDI, 1990-2021. Expected value, based on SDI and ASDR in all 21 GBD regions, are shown as a solid line; expected values based on a calculation accounting for the SDI and ASDR across all 21 GBD regions. Thirty-two points are plotted for each region and show the observed ASDR for each year from 1990 to 2021 for that region. The shaded area indicates the 95% CI of the expected values. Points above the solid line represent a higher-than-expected value, and those below the line show a lower-than-expected value. DALYs, disability-adjusted life-years; SDI, Socio-demographic index; GBD, Global Burden of Disease Study.

**Figure 6:**
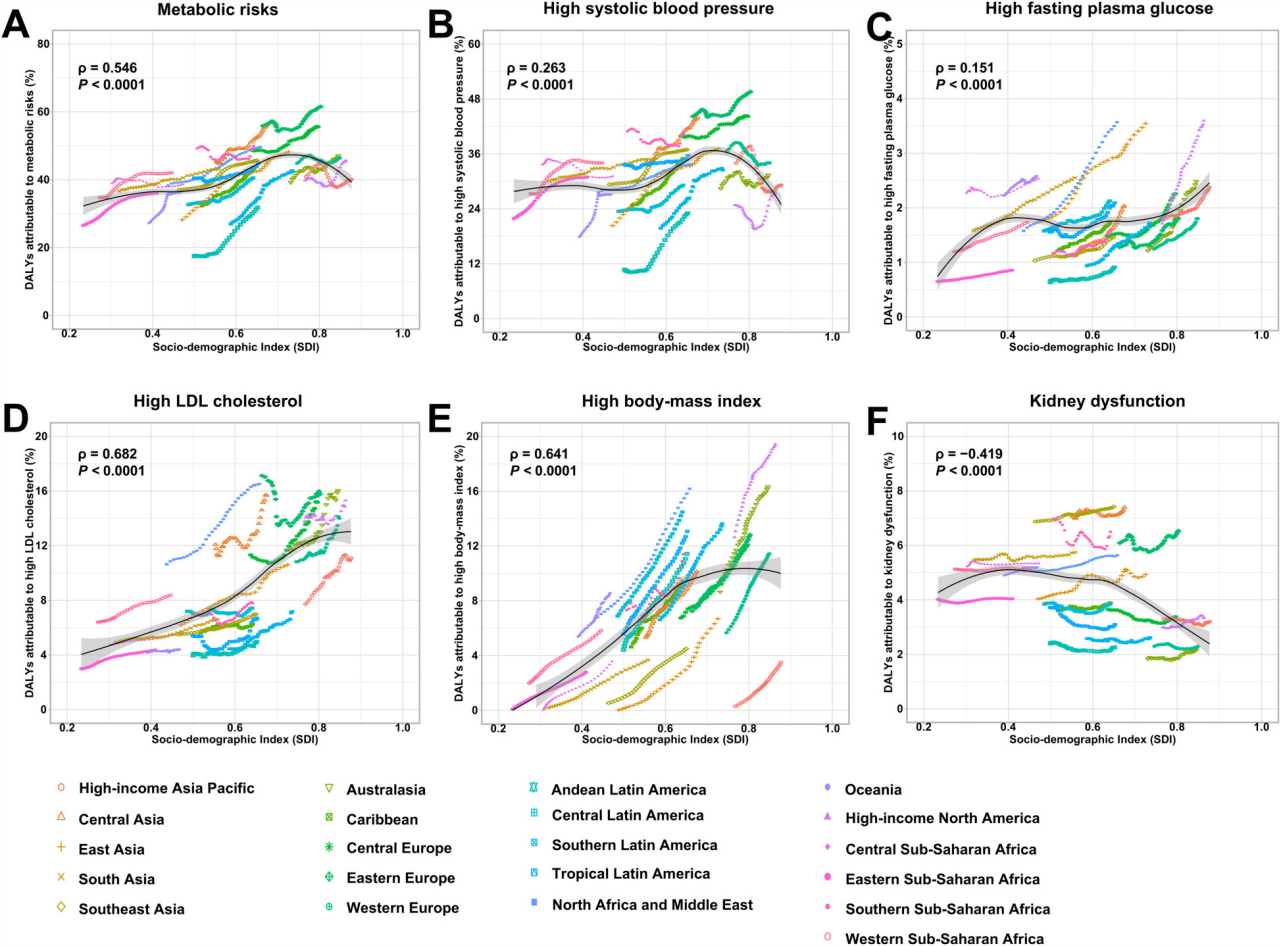
Proportion of disability-adjusted life-years (DALYs) of stroke attributable to metabolic risk factors in young adults, globally and for 21 GBD regions, by SDI and risk factors, 1990-2021. **(A)** Overall metabolic risk. **(B)** High systolic blood pressure. **(C)** High fasting plasma glucose. **(D)** High LDL cholesterol. **(E)** High body-mass index. **(F)** Kidney dysfunction. Expected value, based on SDI and attributable fraction in all 21 GBD regions, are shown as a solid line; expected values based on a calculation accounting for the SDI and attributable fraction across all 21 GBD regions. Thirty-two points are plotted for each region and show the observed proportion for each year from 1990 to 2021 for that region. The shaded area indicates the 95% CI of the expected value. Points above the solid line represent a higher-than-expected value, and those below the line show a lower-than-expected value. SDI, Socio-demographic index; GBD, Global Burden of Disease Study.

In general, the ASDR of stroke attributable to metabolic risks decreased in regions with higher SDI (Figure 5). Additionally, regions with higher SDI levels experienced a faster decline in ASDR between 1990 and 2021 (Table 1). However, the proportion of stroke-related DALYs attributable to metabolic risks increased with rising SDI, except for kidney dysfunction, which showed a negative correlation with SDI (Figure 6). Moreover, the proportion of stroke-DALYs attributable to high fasting plasma glucose was higher in male young adults than female young adults in regions with higher SDI, whereas it was higher in female young adults than male young adults in low- and middle-SDI regions (Supplementary Table S4).

### 3.5 Stroke DALYs attributable to metabolic risks by subtype

In 2021, the global burden of stroke attributable to metabolic risk factors was substantial, with 1,147,521 DALYs from ischemic stroke, 2,267,874 from intracerebral hemorrhage, and 544,954 from subarachnoid hemorrhage (Table 1). The corresponding ASDR were 48.81 (95% UI: 48.9–48.72) for ischemic stroke, 96.47 (95% UI: 96.6–96.34) for intracerebral hemorrhage, and 23.18 (95% UI: 23.24–23.12) for subarachnoid hemorrhage. Between 1990 and 2021, the ASDR declined annually by 0.55% (95% CI: −0.59 to −0.51) for ischemic stroke, 0.74% (95% CI: −0.89 to −0.59) for intracerebral hemorrhage, and 1.37% (95% CI: −1.43 to −1.32) for subarachnoid hemorrhage (Table 1).

**Figure 7:**
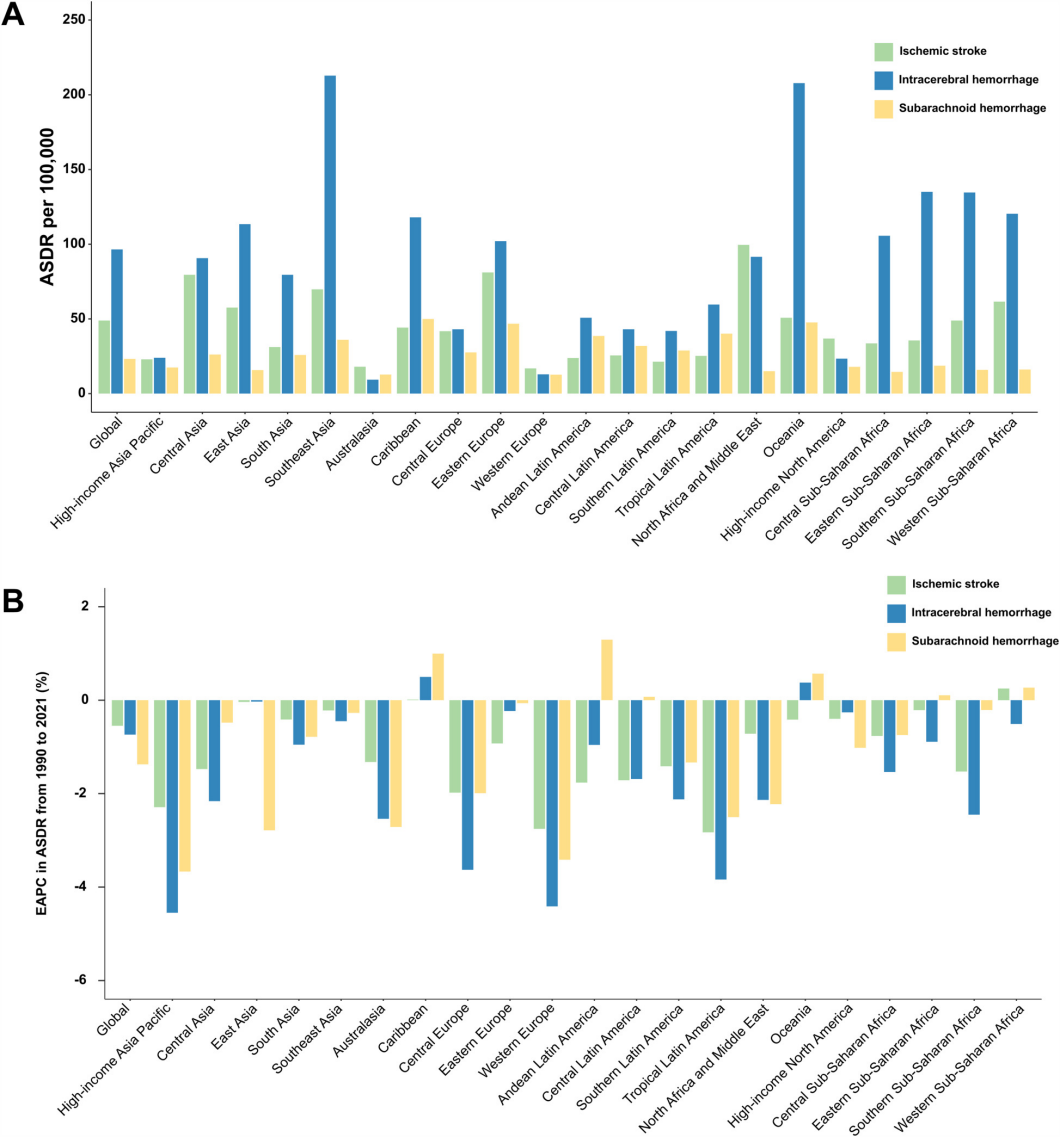
Age-standardized DALYs rate (ASDR) of stroke among young adults in 2021, as well as their change from 1990 to 2021, across the globe and 21 GBD regions, by subtype. **(A)** ASDR of stroke in young adults in 2021. **(B)** Estimated annual percentage change (EAPC) of ASDR for stroke in young adults from 1990 to 2021. GBD, Global Burden of Disease Study.

**Figure 8:**
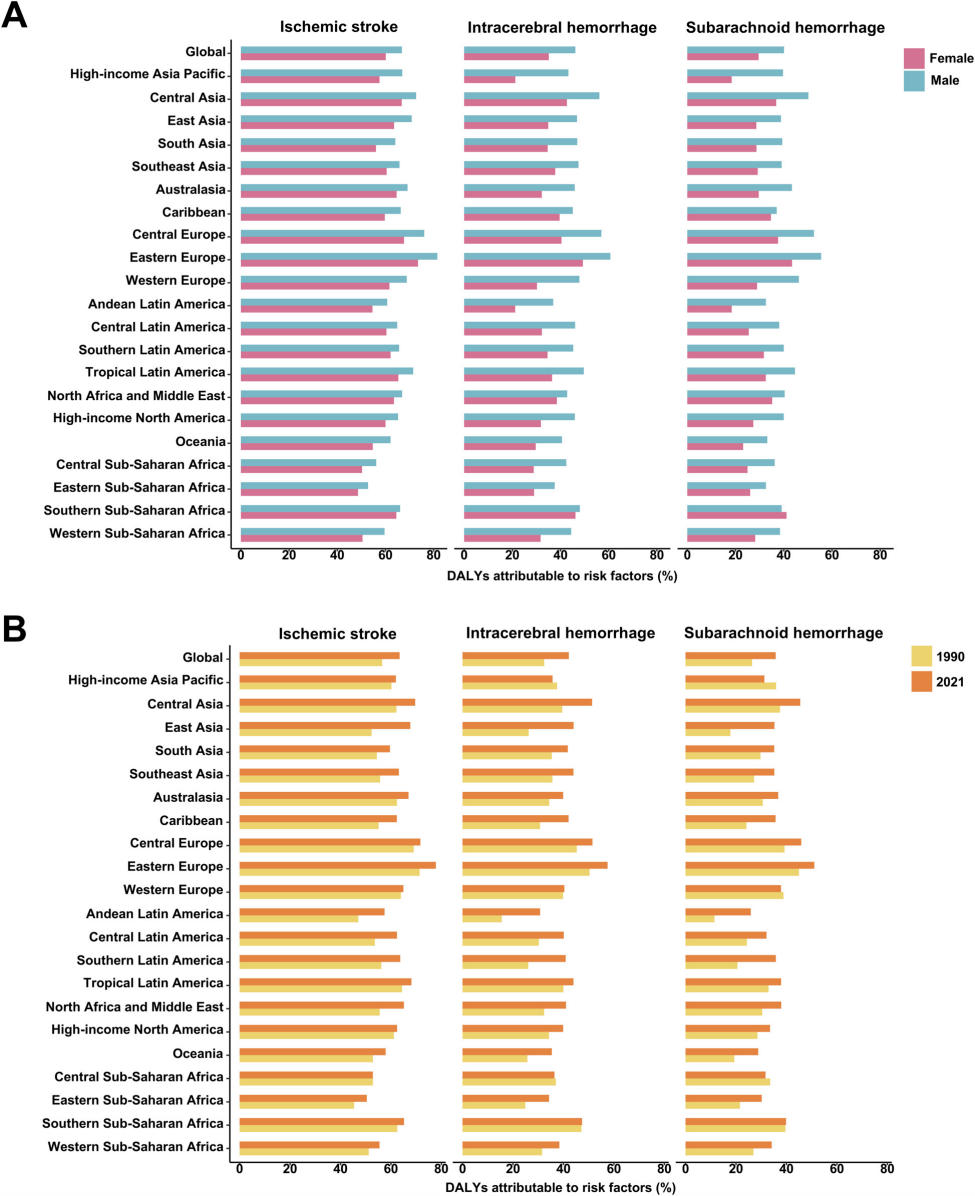
Proportion of disability-adjusted life-years (DALYs) attributable to metabolic risk factors for strokes' subtype among young adults by sex and year, globally and for 21 GBD regions. **(A)** The fractions of DALYs due to strokes' subtype attributable to metabolic risk factors for female and male young adults in 2021. **(B)** The fractions of DALYs due to strokes' subtype attributable to metabolic risk factors for both sexes combined in 1990 and 2021.

In 2021, the highest ASDR for ischemic stroke was recorded in North Africa and the Middle East (99.52 [95% UI: 99.95–99.09), while Oceania had the highest ASDR for intracerebral hemorrhage (207.74 [95% UI: 212.27–203.29) (Figure 7A, Supplementary Table S6–S7). The highest ASDR for subarachnoid hemorrhage was observed in the Caribbean (49.92 [95% UI: 51.1–48.76) (Supplementary Table S8). From 1990 to 2021, an increase in ASDR for ischemic stroke was observed only in Sub-Saharan Africa (EAPC = 0.25) (Figure 7B, Supplementary Table S6). Similarly, ASDR for intracerebral hemorrhage increased in the Caribbean (EAPC = 0.50) and Oceania (EAPC = 0.37) (Supplementary Table S7). For subarachnoid hemorrhage, ASDR increased in the Caribbean (EAPC = 0.99), Oceania (EAPC = 0.57), Western Sub-Saharan Africa (EAPC = 0.27), and Eastern Sub-Saharan Africa (EAPC = 0.10) (Supplementary Table S8). For both males and females, Eastern Europe had the highest proportion of DALYs from ischemic stroke, intracerebral hemorrhage, and subarachnoid hemorrhage attributable to metabolic risks (Figure 8A). Moreover, the metabolic-risk-attributable proportion of DALYs from these stroke subtypes increased globally and across all 21 GBD regions between 1990 and 2021 (Figure 8B).

**Figure 9:**
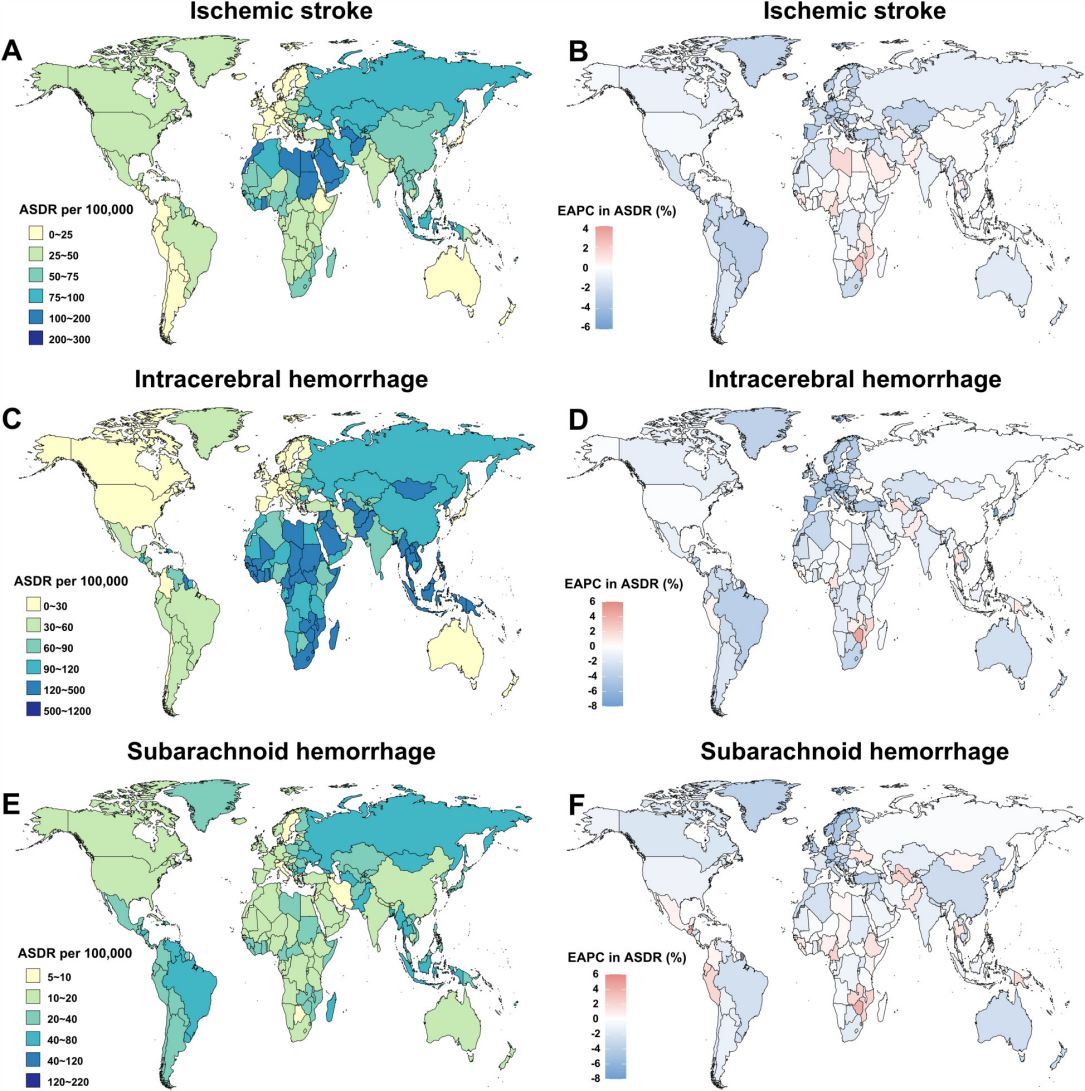
The global age-standardized DALYs rate (ASDR) of stroke in young adults across 204 countries and territories in 2021, and their estimated annual percentage changes (EAPC) from 1990 to 2021, by subtype. The ASDR **(A)** and its EAPC **(B)** for ischemic stroke. ASDR **(C)** and its EAPC **(D)** for intracerebral hemorrhage. ASDR **(E)** and its EAPC **(F)** for subarachnoid hemorrhage.

At the country level, Nauru had the highest ASDR for all three stroke types in 2021: ischemic stroke (221.86 [95% UI: 456.04–90.68), intracerebral hemorrhage (1016.29 [95% UI: 1428.42–700.64), and subarachnoid hemorrhage (217.67 [95% UI: 448.24–9.13) (Figures 9A,C,E, Supplementary Table S9-S11). Between 1990 and 2021, ASDR for ischemic stroke increased in 35 countries and territories, with Zimbabwe experiencing the fastest rise (2.3% per year [95% CI: 1.76–2.85) (Figure 9B, Supplementary Table S9). Similarly, ASDR for intracerebral hemorrhage increased in 22 countries and territories, with the highest rise also seen in Zimbabwe (4.57% per year [95% CI: 3.3–5.85) (Figure 9D, Supplementary Table S10). For subarachnoid hemorrhage, ASDR increased in 51 countries and territories, with Zimbabwe again showing the fastest increase (4.1% per year [95% CI: 3.1–5.1) (Figure 9F, Supplementary Table S11).

## 4 Discussion

This study provides a comprehensive assessment of the global burden and trends of stroke and its subtypes attributable to metabolic risk factors among young adults from 1990 to 2021, using data from the GBD 2021. Our findings reveal that in 2021, metabolic risks accounted for 45.44% of the total stroke-related DALYs in young adults, with high systolic blood pressure emerging as the leading contributor, followed by high LDL cholesterol, high BMI, kidney dysfunction, and high fasting plasma glucose. While the ASDR of stroke attributable to metabolic risks declined globally by an average of 0.78% per year from 1990 to 2021, the absolute number of stroke-related DALYs due to these risk factors increased by 22.23%, highlighting a growing burden despite overall reductions in stroke rates. Regionally, East Asia had the highest absolute number of strokes DALYs attributable to metabolic risks, while Southeast Asia exhibited the highest ASDR. Eastern Europe recorded the highest proportion of stroke-related DALYs due to metabolic risks, particularly high systolic blood pressure and high BMI. Notably, the burden varied by sex, with male young adults generally experiencing a higher proportion of stroke DALYs due to metabolic risks, except for high LDL cholesterol and high BMI, which were more prominent among female young adults. At the national level, Nauru exhibited the highest ASDR for stroke attributable to metabolic risks, while Belarus reported the highest proportion of stroke-related DALYs due to these factors. Additionally, several low- and middle-income countries, including Zimbabwe and Lesotho, experienced the fastest increases in ASDR between 1990 and 2021. These findings underscore the increasing burden of metabolic risks in stroke epidemiology, particularly in regions undergoing rapid urbanization and lifestyle transitions. The rise in metabolic diseases as major stroke risk factors underscores the urgent need for age-specific prevention strategies. Early intervention could mitigate both the immediate and long-term consequences of stroke in this age group (25, 26).

Our results align with prior studies showing that metabolic risk factors, particularly high systolic blood pressure, high LDL cholesterol, and high BMI, play a major role in stroke burden among young adults. For instance, GBD 2019 Stroke Collaborators highlighted the increasing contribution of metabolic risk factors to global stroke burden, with high blood pressure remaining the dominant contributor across all age groups, consistent with our findings that high systolic blood pressure accounted for the highest proportion of stroke DALYs in young adults (27). Additionally, a study by O'Donnell et al. based on the INTERSTROKE study emphasized that metabolic risks contribute significantly to stroke in younger populations, supporting our observations of the increasing absolute burden despite a declining age-standardized rate (28). Furthermore, our findings on the regional disparities in metabolic risk-attributable stroke burden resonate with previous GBD studies, which reported a particularly high burden in Eastern Europe, where hypertension prevalence remains elevated (29, 30). The observed increasing trend in the proportion of stroke DALYs attributable to metabolic risks in high-income regions, despite overall declining ASDRs, is consistent with prior evidence indicating a shift in risk factor patterns and lifestyle changes, such as increasing obesity rates and dietary risk factors (9). Our findings align with previous study indicating that metabolic risk factors play a larger role in ischemic stroke than in hemorrhagic stroke, particularly in high-income settings where dyslipidemia and obesity have become more prevalent (27, 31, 32).

Although the ASDR of stroke attributable to metabolic risks has generally declined over the past three decades, the absolute number of stroke-related DALYs and their proportion of total stroke burden have increased significantly. This highlights a growing public health challenge. Firstly, the growth of the global population, particularly within the young adults age group (https://www.un.org/development/desa/pd/content/youth-population-trends-and-sustainable-development), has contributed to a higher absolute burden of stroke attributable to metabolic risks. As the population size increases, even a stable or declining age-standardized rate can result in a higher total number of cases. Secondly, the rising prevalence of metabolic risk factors such as high systolic blood pressure, high BMI, and high fasting plasma glucose among young adults has exacerbated the stroke burden in this group (8). Unhealthy lifestyle patterns, including poor dietary habits, physical inactivity, and increasing obesity rates, are likely major contributors to this trend (33). This trend presents a significant public health challenge because stroke in young adults can lead to long-term disability, reduced productivity, and increased healthcare costs. Unlike stroke in older populations, stroke in young adults affects individuals in their most economically productive years, leading to profound social and economic consequences. Additionally, a higher burden of metabolic risk factors at younger ages increases the risk of recurrent strokes and other cardiovascular diseases later in life, further straining healthcare systems.

Our findings reveal significant regional disparities in the stroke burden attributable to metabolic risks. East Asia reported the highest absolute number of stroke-related DALYs, while Southeast Asia had the highest ASDR for stroke, indicating a disproportionately high burden in these regions (34). The highest proportion of DALYs attributable to metabolic risks was found in Eastern Europe, which experienced substantial contributions from high systolic blood pressure and high BMI. These findings are consistent with existing literature that reports the rapid rise of metabolic diseases in Eastern European countries due to dietary patterns, urbanization, and limited access to healthcare resources (8, 35). Interestingly, the specific metabolic risk factors contributing to the stroke burden varied across regions. High systolic blood pressure was the predominant risk factor in Eastern Europe, while in regions such as High-income North America and North Africa, high LDL cholesterol and high BMI were more significant contributors. These regional differences reflect not only variations in lifestyle and dietary habits but also disparities in healthcare access and the implementation of preventive strategies (36, 37). The differences between male and female young adults populations in stroke burden further emphasize the need for sex-specific approaches to stroke prevention (38), as male young adults generally had a higher proportion of stroke DALYs attributable to metabolic risks, except for high LDL cholesterol and high BMI, which were more pronounced in female young adults.

At the national level, countries like Nauru, Belarus, and Saudi Arabia exhibited the highest proportions of stroke DALYs attributable to metabolic risks, particularly high systolic blood pressure and high BMI. These nations also report some of the highest rates of metabolic diseases globally, driven by unhealthy dietary habits, high obesity rates, and insufficient public health initiatives focused on prevention and early intervention (35, 39, 40). In contrast, countries such as Zimbabwe and Lesotho have seen some of the fastest increases in ASDR for stroke attributable to metabolic risks, highlighting the emerging burden of metabolic diseases in low- and middle-income countries (LMICs) (41). This rising burden is particularly concerning, given the limited healthcare infrastructure and resources for stroke management and prevention in these regions (42). As these countries continue to urbanize and experience economic growth, it is critical to implement comprehensive public health strategies that address the growing prevalence of metabolic diseases and their role in stroke. The identification of countries with high proportions of stroke DALYs attributable to specific metabolic risk factors—such as high LDL cholesterol in Kuwait or high fasting plasma glucose in Saudi Arabia—provides valuable insights into the public health priorities needed for targeted interventions. For example, public health efforts aimed at improving dietary habits and promoting physical activity could address the rising rates of high cholesterol and BMI, while diabetes management programs could be prioritized in regions with high rates of fasting plasma glucose (43).

The increasing burden of stroke attributable to metabolic risks in young adults may be linked to global shifts in dietary habits, such as the growing consumption of processed and high-fat foods (44), increased sugar intake (45), and reduced physical activity due to urbanization and sedentary lifestyles (46). Additionally, we have highlighted how regional variations in metabolic risk factors may be influenced by socioeconomic transitions, including changes in food accessibility, healthcare infrastructure, and preventive health strategies (9). Moreover, the COVID-19 pandemic may have exacerbated metabolic risk factors through disruptions in healthcare access (47), increased sedentary behavior and weight gain (48), and stress-related metabolic changes (49). While the long-term effects of COVID-19 on metabolic health and stroke risk in young adults remain an area for further research, preliminary evidence suggests that pandemic-related changes in lifestyle and healthcare utilization may have contributed to worsening metabolic risk profiles in certain regions (50).

Notably, male young adults had a higher proportion of stroke DALYs attributable to metabolic risks, except for high LDL cholesterol and high BMI, which were more prominent in female young adults. This gender disparity can be attributed to several physiological, behavioral, and healthcare-related factors. Firstly, sex-specific differences in lipid metabolism and fat distribution may contribute to the higher impact of high LDL cholesterol and high BMI in females (51). Women generally have a higher percentage of body fat, and hormonal influences, particularly estrogen, play a role in lipid regulation and cardiovascular risk (52). However, after puberty, males tend to have higher blood pressure and fasting glucose levels, which may explain their greater burden of stroke attributable to hypertension and high fasting plasma glucose (53). Second, lifestyle factors, such as diet, physical activity, and smoking, differ between males and females, potentially influencing metabolic risk factors (54). For example, higher smoking rates in male young adults may exacerbate the effects of metabolic risks such as hypertension and diabetes on stroke burden (55). In contrast, female young adults may experience unique risk factors related to hormonal changes, pregnancy-related metabolic changes, and polycystic ovary syndrome (PCOS), which can contribute to dyslipidemia and obesity-related stroke risk (56). Finally, gender differences in healthcare access and risk factor management may play a role. Women, especially younger individuals, may be less likely to receive timely cardiovascular risk assessment and intervention compared to men, which could influence the overall burden of metabolic risk-related stroke in this population (57).

This study has important implications for public health policy and stroke prevention efforts. The increasing burden of stroke in young adults, particularly due to metabolic risks, necessitates urgent attention from policymakers to develop tailored prevention and management strategies. Early screening and intervention, particularly for metabolic risk factors, could significantly reduce stroke incidence in this age group. Public health campaigns should focus on raising awareness about the link between metabolic diseases and stroke. The findings also emphasize the need for regional and sex-specific approaches, as the contributors to stroke burden vary across geographical regions and demographic groups. Additionally, integrating metabolic risk factor management into existing stroke prevention programs will be crucial, especially in regions undergoing rapid epidemiological transitions.

Our study has some limitations. Firstly, the reliance on GBD data means that some regions with limited data may have been underrepresented, and the accuracy of the estimates may be influenced by variations in reporting practices across countries. Additionally, the analysis does not account for the impact of emerging risk factors, such as mental health conditions or environmental exposures, which may also contribute to the rising burden of stroke attributable to metabolic risks in young adults. Future research should explore the role of these factors in more detail and investigate the effectiveness of public health interventions tailored to metabolic risks in preventing stroke in younger populations.

## 5 Conclusions

In conclusion, metabolic risks continue to drive the increasing global burden of stroke in young adults, underscoring the need for targeted public health strategies focused on early identification and prevention.

## Data Availability

The original contributions presented in the study are included in the article/Supplementary Material, further inquiries can be directed to the corresponding author.
