# Supplementary material for: Global burden and trend of stroke attributable to metabolic risks among young adults (20–39 years old) from 1990 to 2021
Source: Front Cardiovasc Med. 2025 Jun 2;12:1561052. doi: 10.3389/fcvm.2025.1561052 (PMC12171433; doi:10.3389/fcvm.2025.1561052)
Supplement: Supplementary file 1 [file Datasheet1.docx]

**Supplementary Table S1. Definitions of metabolic risk factors of stroke and theoretical minimum risk exposure level**

| Metabolic risk factors | Exposure definition | Theoretical minimum risk exposure level |
| --- | --- | --- |
| High systolic blood pressure | Systolic blood pressure, measured in mm Hg | 110–115 mm Hg |
| High fasting plasma glucose | Serum fasting plasma glucose measured in mmol/L | 4.8–5.4 mmol/L |
| High low-density lipoprotein cholesterol | Serum low-density lipoprotein, measured in mmol/L | 0.7–1.3 mmol/L |
| High body-mass index | Body-mass index, measured in kg/m² | 20–25 kg/m² |
| kidney dysfunction | Proportion of the population with ACR >30 mg/g or GFR <60 mL/min/1.73 m², excluding end-stage renal disease | GFR >60 mL/min/1.73 m² and ACR <30 mg/g |

Supplementary Table S2. Socio-demographic Index (SDI) quintiles for 204 countries and territories estimated in GBD 2021

| **SDI Quintile** | **Locations included based on SDI values in 2021 from GBD 2021 results** |
| --- | --- |
| High SDI | Andorra, Australia, Austria, Bahamas, Belgium, Bermuda, Brunei Darussalam, Canada, Cyprus, Czechia, Denmark, Estonia, Finland, France, Georgia, Germany, Greenland, Iceland, Ireland, Israel, Italy, Japan, Kuwait, Latvia, Lithuania, Luxembourg, Monaco, Netherlands, New Zealand, Norway, Poland, Puerto Rico, Qatar, Republic of Korea, Russian Federation, San Marino, Saudi Arabia, Singapore, Slovakia, Slovenia, Sweden, Switzerland, Taiwan (Province of China), United Arab Emirates, United Kingdom, United States of America, United States Virgin Islands |
| High-middle SDI | Albania, American Samoa, Antigua and Barbuda, Argentina, Armenia, Azerbaijan, Bahrain, Barbados, Belarus, Bosnia and Herzegovina, Bulgaria, Chile, China, Cook Islands, Costa Rica, Croatia, Dominica, Georgia, Greece, Guam, Hungary, Iran (Islamic Republic of), Jordan, Kazakhstan, Lebanon, Libya, Malaysia, Malta, Mauritius, Montenegro, Niue, North Macedonia, Northern Mariana Islands, Oman, Palau, Panama, Portugal, Republic of Moldova, Romania, Saint Kitts and Nevis, Serbia, Seychelles, Spain, Sri Lanka, Trinidad and Tobago, Turkey, Ukraine, Uruguay |
| Middle SDI | Algeria, Belize, Botswana, Brazil, Colombia, Cuba, Dominican Republic, Ecuador, Equatorial Guinea, Fiji, Gabon, Grenada, Guyana, Indonesia, Iraq, Jamaica, Maldives, Mexico, Mongolia, Namibia, Nauru, Palestine, Paraguay, Peru, Philippines, Saint Lucia, Saint Vincent and the Grenadines, South Africa, Suriname, Syrian Arab Republic, Thailand, Tokelau, Tonga, Tunisia, Turkmenistan, Uzbekistan, Viet Nam |
| Low-middle SDI | Bangladesh, Bhutan, Bolivia (Plurinational State of), Cabo Verde, Cambodia, Cameroon, Comoros, Congo, Democratic People's Republic of Korea, Djibouti, Egypt, El Salvador, Eswatini, Ghana, Guatemala, Honduras, India, Kenya, Kiribati, Kyrgyzstan, Lao People's Democratic Republic, Lesotho, Marshall Islands, Mauritania, Micronesia (Federated States of), Morocco, Myanmar  Nicaragua, Nigeria, Pakistan, Samoa, Sao Tome and Principe, Sudan, Tajikistan, Tuvalu, Vanuatu, Venezuela (Bolivarian Republic of), Zambia, Zimbabwe |
| Low SDI | Afghanistan, Angola, Benin, Burkina Faso, Burundi, Central African Republic, Chad, Côte d'Ivoire, Democratic Republic of the Congo, Eritrea, Ethiopia, Gambia, Guinea, Guinea-Bissau, Haiti, Liberia, Madagascar, Malawi, Mali, Mozambique, Nepal, Niger  Papua New Guinea, Rwanda, Senegal, Sierra Leone, Solomon Islands, Somalia, South Sudan, Timor-Leste, Togo, Uganda, United Republic of Tanzania, Yemen |

Supplementary Table S3. 21 GBD world regions and 204 countries and territories within each region.

| **GBD World Region (n=21)** | **Countries and territories (n=204)** |
| --- | --- |
| Central Asia | Armenia, Azerbaijan, Georgia, Kazakhstan, Kyrgyzstan, Mongolia, Tajikistan, Turkmenistan, Uzbekistan |
| Central Europe | Albania, Bosnia and Herzegovina, Bulgaria, Croatia, Czech Republic, Hungary, Montenegro, North Macedonia, Poland (subnational), Romania, Serbia, Slovakia, Slovenia |
| Eastern Europe | Belarus, Estonia, Latvia, Lithuania, Moldova, Russia (subnational), Ukraine |
| Australasia | Australia, New Zealand (subnational Māori + non-Māori) |
| High-income Asia Pacific | Brunei, Japan (subnational), Singapore, South Korea |
| High-income North America | Canada, Greenland, United States (subnational) |
| Southern Latin America | Argentina, Chile, Uruguay |
| Western Europe | Andorra, Austria, Belgium, Cyprus, Denmark, Finland, France, Germany, Greece, Iceland, Ireland, Israel, Italy (subnational), Luxembourg, Malta, Monaco, Netherlands, Norway (subnational), Portugal, San Marino, Spain, Sweden (subnational), Switzerland, United Kingdom (subnational) |
| Andean Latin America | Bolivia, Ecuador, Peru |
| Caribbean | Antigua and Barbuda, Bahamas, Barbados, Belize, Bermuda, Cuba, Dominica, Dominican Republic, Grenada, Guyana, Haiti, Jamaica, Puerto Rico, Saint Kitts and Nevis, Saint Lucia, Saint Vincent and the Grenadines, Suriname, Trinidad and Tobago, US Virgin Islands |
| Central Latin America | Colombia, Costa Rica, El Salvador, Guatemala, Honduras, Mexico (subnational), Nicaragua, Panama, Venezuela |
| Tropical Latin America | Brazil (subnational), Paraguay |
| North Africa and Middle East | Afghanistan, Algeria, Bahrain, Egypt, Iran (subnational), Iraq, Jordan, Kuwait, Lebanon, Libya, Morocco, Oman, Palestine, Qatar, Saudi Arabia, Sudan, Syria, Tunisia, Türkiye, United Arab Emirates, Yemen |
| South Asia | Bangladesh, Bhutan, India (subnational), Nepal, Pakistan (subnational) |
| East Asia | China, North Korea, Taiwan (province of China) |
| Oceania | American Samoa, Cook Islands, Federated States of Micronesia, Fiji, Guam, Kiribati, Marshall Islands, Nauru, Niue, Northern Mariana Islands, Palau, Papua New Guinea, Samoa, Solomon Islands, Tokelau, Tonga, Tuvalu, Vanuatu |
| Southeast Asia | Cambodia, Indonesia (subnational), Laos, Malaysia, Maldives, Mauritius, Myanmar, Philippines (subnational), Seychelles, Sri Lanka, Thailand, Timor-Leste, Vietnam |
| Central sub-Saharan Africa | Angola, Central African Republic, Congo (Brazzaville), Democratic Republic of the Congo, Equatorial Guinea, Gabon |
| Eastern sub-Saharan Africa | Burundi, Comoros, Djibouti, Eritrea, Ethiopia (subnational), Kenya (subnational), Madagascar, Malawi, Mozambique, Rwanda, Somalia, South Sudan, Tanzania, Uganda, Zambia |
| Southern sub-Saharan Africa | Botswana, eSwatini, Lesotho, Namibia, South Africa (subnational), Zimbabwe |
| Western sub-Saharan Africa | Benin, Burkina Faso, Cape Verde, Cameroon, Chad, Côte d’Ivoire, Gambia, Ghana, Guinea, Guinea-Bissau, Liberia, Mali, Mauritania, Niger, Nigeria (subnational), São Tomé and Príncipe, Senegal, Sierra Leone, Togo |

**Supplementary Table S4. Proportion of stroke-related disability-adjusted life years (DALYs) attributable to metabolic risk factors among young adults in 1990 and 2021, by sex.**

| Characteristics | 1990 | | |  | 2021 | | |
| --- | --- | --- | --- | --- | --- | --- | --- |
|  | Total (%) | Female (%) | Male (%) |  | Total (%) | Female (%) | Male (%) |
| Overall metabolic risk |  |  |  |  |  |  |  |
| Global | 35.15 | 32.02 | 37.72 |  | 45.44 | 40.69 | 48.66 |
| Socio-demographic index |  |  |  |  |  |  |  |
| High | 42.15 | 35.57 | 47.45 |  | 48.42 | 41.95 | 53.17 |
| High-middle | 36.92 | 33.45 | 39.28 |  | 52.9 | 47.95 | 55.44 |
| Middle | 33.24 | 30.8 | 35.25 |  | 46.38 | 41.92 | 49.09 |
| Low-middle | 35.07 | 32.38 | 37.6 |  | 43.92 | 40.08 | 46.85 |
| Low | 31.61 | 29.67 | 33.53 |  | 37.93 | 34.1 | 41.27 |
| GBD regions |  |  |  |  |  |  |  |
| High-income Asia Pacific | 40.89 | 32.84 | 46.22 |  | 40.11 | 31.07 | 46.26 |
| Central Asia | 45.51 | 39.79 | 50.92 |  | 56.39 | 51.82 | 59.55 |
| East Asia | 28.21 | 26.5 | 29.38 |  | 48.21 | 43.38 | 50.21 |
| South Asia | 36.64 | 32.76 | 39.94 |  | 43.2 | 38.05 | 47.21 |
| Southeast Asia | 37.08 | 33.06 | 40.96 |  | 45.72 | 41.2 | 48.63 |
| Australasia | 39.19 | 30.43 | 47.19 |  | 47.12 | 41.7 | 51.99 |
| Caribbean | 32.3 | 29.53 | 36.14 |  | 43.21 | 41.26 | 45.88 |
| Central Europe | 48.76 | 41.85 | 53.92 |  | 55.58 | 49.3 | 60.19 |
| Eastern Europe | 55.86 | 51.34 | 58.73 |  | 61.54 | 56.53 | 64.25 |
| Western Europe | 44.83 | 38.95 | 49.56 |  | 46.39 | 39.73 | 52.51 |
| Andean Latin America | 17.43 | 14.92 | 19.84 |  | 31.83 | 24.68 | 37.54 |
| Central Latin America | 32.67 | 28.51 | 37.73 |  | 40.59 | 35.06 | 45.52 |
| Southern Latin America | 26.97 | 20.31 | 33.19 |  | 42.52 | 38.48 | 46.11 |
| Tropical Latin America | 40.49 | 34.03 | 46.65 |  | 44.87 | 39.03 | 50.61 |
| North Africa and Middle East | 38.33 | 39.01 | 37.67 |  | 49.61 | 48.82 | 50.29 |
| High-income North America | 39.76 | 32.63 | 47.15 |  | 45.63 | 40.92 | 50.24 |
| Oceania | 27.18 | 22.37 | 31.62 |  | 36.47 | 31.7 | 40.9 |
| Central Sub-Saharan Africa | 38.95 | 36.29 | 41.31 |  | 38.59 | 32.95 | 43.3 |
| Eastern Sub-Saharan Africa | 26.57 | 24.53 | 28.26 |  | 36.05 | 32.49 | 38.42 |
| Southern Sub-Saharan Africa | 49.27 | 45.57 | 53.09 |  | 49.95 | 50.49 | 49.56 |
| Western Sub-Saharan Africa | 34.75 | 30.94 | 38.28 |  | 42.07 | 35.83 | 47.46 |
| High systolic blood pressure |  |  |  |  |  |  |  |
| Global | 27.08 | 22.24 | 31.07 |  | 35.43 | 28.07 | 40.42 |
| Socio-demographic index |  |  |  |  |  |  |  |
| High | 31.92 | 22.79 | 39.28 |  | 32.17 | 21.5 | 40.01 |
| High-middle | 27.75 | 21.91 | 31.71 |  | 41.12 | 32.05 | 45.76 |
| Middle | 25.36 | 21.29 | 28.71 |  | 35.71 | 28.06 | 40.35 |
| Low-middle | 27.91 | 23.79 | 31.77 |  | 35.25 | 29.27 | 39.82 |
| Low | 25.7 | 22.46 | 28.91 |  | 30.99 | 25.61 | 35.67 |
| GBD regions |  |  |  |  |  |  |  |
| High-income Asia Pacific | 34 | 23.45 | 40.98 |  | 29.21 | 16.51 | 37.86 |
| Central Asia | 34.2 | 25.86 | 42.1 |  | 44.03 | 35.33 | 50.06 |
| East Asia | 20.34 | 16.74 | 22.77 |  | 37.17 | 28.33 | 40.85 |
| South Asia | 30.05 | 25.06 | 34.27 |  | 34.84 | 27.77 | 40.33 |
| Southeast Asia | 29.35 | 24.05 | 34.47 |  | 36.94 | 29.71 | 41.57 |
| Australasia | 28.45 | 16.57 | 39.31 |  | 31.4 | 23.09 | 38.89 |
| Caribbean | 24.75 | 21.43 | 29.36 |  | 34.97 | 32.56 | 38.27 |
| Central Europe | 39.86 | 31.05 | 46.42 |  | 44.2 | 34.21 | 51.52 |
| Eastern Europe | 44.21 | 36.12 | 49.35 |  | 49.59 | 40.35 | 54.61 |
| Western Europe | 36.77 | 29.03 | 43 |  | 34.01 | 24.02 | 43.2 |
| Andean Latin America | 10.72 | 6.8 | 14.48 |  | 23.05 | 13.64 | 30.56 |
| Central Latin America | 23.44 | 17.43 | 30.73 |  | 29.17 | 21.12 | 36.34 |
| Southern Latin America | 19.78 | 11.48 | 27.51 |  | 32.53 | 26.28 | 38.1 |
| Tropical Latin America | 33.66 | 26.44 | 40.57 |  | 35.6 | 28.26 | 42.81 |
| North Africa and Middle East | 27.62 | 25.92 | 29.25 |  | 33.7 | 30.03 | 36.83 |
| High-income North America | 24.8 | 15.31 | 34.63 |  | 27.37 | 19.42 | 35.15 |
| Oceania | 17.88 | 11.32 | 23.83 |  | 27.14 | 20.74 | 32.99 |
| Central Sub-Saharan Africa | 33.65 | 29.61 | 37.25 |  | 31.14 | 23.35 | 37.61 |
| Eastern Sub-Saharan Africa | 21.88 | 18.11 | 25 |  | 30.94 | 25.33 | 34.67 |
| Southern Sub-Saharan Africa | 40.95 | 34.37 | 47.76 |  | 40.79 | 37.28 | 43.33 |
| Western Sub-Saharan Africa | 27.3 | 22.7 | 31.57 |  | 34.04 | 26.66 | 40.39 |
| High fasting plasma glucose |  |  |  |  |  |  |  |
| Global | 1.47 | 1.34 | 1.57 |  | 2.42 | 2.32 | 2.48 |
| Socio-demographic index |  |  |  |  |  |  |  |
| High | 1.69 | 1.42 | 1.91 |  | 3.78 | 3.41 | 4.05 |
| High-middle | 1.65 | 1.51 | 1.75 |  | 3.14 | 2.85 | 3.28 |
| Middle | 1.47 | 1.34 | 1.57 |  | 2.48 | 2.38 | 2.55 |
| Low-middle | 1.3 | 1.25 | 1.35 |  | 2.15 | 2.22 | 2.1 |
| Low | 1.16 | 1.2 | 1.11 |  | 1.56 | 1.63 | 1.49 |
| GBD regions |  |  |  |  |  |  |  |
| High-income Asia Pacific | 1.8 | 1.62 | 1.91 |  | 2.38 | 2.1 | 2.57 |
| Central Asia | 1.12 | 1.08 | 1.16 |  | 2.04 | 2.15 | 1.96 |
| East Asia | 1.74 | 1.57 | 1.86 |  | 3.54 | 3.13 | 3.72 |
| South Asia | 1.59 | 1.51 | 1.65 |  | 2.55 | 2.54 | 2.56 |
| Southeast Asia | 1.03 | 0.97 | 1.1 |  | 1.46 | 1.5 | 1.43 |
| Australasia | 1.22 | 1 | 1.42 |  | 2.49 | 2.23 | 2.71 |
| Caribbean | 1.21 | 1.15 | 1.31 |  | 1.73 | 1.65 | 1.85 |
| Central Europe | 1.48 | 1.25 | 1.66 |  | 2.26 | 1.96 | 2.48 |
| Eastern Europe | 1.28 | 1.34 | 1.24 |  | 1.81 | 1.84 | 1.79 |
| Western Europe | 1.25 | 1.11 | 1.36 |  | 1.8 | 1.73 | 1.87 |
| Andean Latin America | 0.63 | 0.64 | 0.63 |  | 0.91 | 0.93 | 0.89 |
| Central Latin America | 1.58 | 1.58 | 1.57 |  | 2.13 | 2.21 | 2.05 |
| Southern Latin America | 0.94 | 0.89 | 0.99 |  | 1.72 | 1.76 | 1.68 |
| Tropical Latin America | 1.56 | 1.29 | 1.81 |  | 2.09 | 1.91 | 2.27 |
| North Africa and Middle East | 1.58 | 1.54 | 1.61 |  | 3.57 | 3.7 | 3.46 |
| High-income North America | 1.71 | 1.25 | 2.19 |  | 3.59 | 3.17 | 4.02 |
| Oceania | 2.22 | 2.05 | 2.37 |  | 2.58 | 2.5 | 2.67 |
| Central Sub-Saharan Africa | 2.27 | 2.32 | 2.22 |  | 2.54 | 2.47 | 2.59 |
| Eastern Sub-Saharan Africa | 0.65 | 0.71 | 0.6 |  | 0.86 | 0.93 | 0.81 |
| Southern Sub-Saharan Africa | 1.17 | 1.23 | 1.11 |  | 1.6 | 1.97 | 1.34 |
| Western Sub-Saharan Africa | 1.2 | 1.17 | 1.22 |  | 1.74 | 1.72 | 1.75 |
| High LDL cholesterol |  |  |  |  |  |  |  |
| Global | 7 | 8.33 | 5.92 |  | 9.13 | 11.26 | 7.69 |
| Socio-demographic index |  |  |  |  |  |  |  |
| High | 10.99 | 12.3 | 9.94 |  | 15.26 | 17.63 | 13.52 |
| High-middle | 9.18 | 11.24 | 7.79 |  | 12.66 | 16.93 | 10.47 |
| Middle | 6.23 | 7.63 | 5.09 |  | 9.31 | 12.18 | 7.58 |
| Low-middle | 5.46 | 6.68 | 4.33 |  | 7.5 | 9.21 | 6.2 |
| Low | 4.44 | 5.33 | 3.57 |  | 6.06 | 7.04 | 5.22 |
| GBD regions |  |  |  |  |  |  |  |
| High-income Asia Pacific | 7.72 | 9.94 | 6.26 |  | 11.08 | 14.25 | 8.91 |
| Central Asia | 12.08 | 13.53 | 10.7 |  | 15.68 | 19.66 | 12.92 |
| East Asia | 6.21 | 8.11 | 4.93 |  | 10.61 | 15.46 | 8.59 |
| South Asia | 4.93 | 6.13 | 3.95 |  | 6.61 | 8.35 | 5.29 |
| Southeast Asia | 5.49 | 6.37 | 4.65 |  | 6.97 | 9.04 | 5.66 |
| Australasia | 11.57 | 12.3 | 10.9 |  | 16.04 | 16.49 | 15.62 |
| Caribbean | 5.63 | 5.65 | 5.6 |  | 6.25 | 6.34 | 6.13 |
| Central Europe | 11.32 | 12.42 | 10.5 |  | 15.18 | 18.46 | 12.77 |
| Eastern Europe | 17.12 | 20.29 | 15.11 |  | 15.79 | 19.9 | 13.55 |
| Western Europe | 10.82 | 11.83 | 10.01 |  | 14 | 15.52 | 12.59 |
| Andean Latin America | 3.96 | 4.69 | 3.26 |  | 4.96 | 5.99 | 4.15 |
| Central Latin America | 7.2 | 7.95 | 6.29 |  | 7.43 | 8.85 | 6.16 |
| Southern Latin America | 4.44 | 5.08 | 3.84 |  | 7.17 | 8.39 | 6.09 |
| Tropical Latin America | 5.4 | 5.34 | 5.46 |  | 6.68 | 7.28 | 6.1 |
| North Africa and Middle East | 10.63 | 12.97 | 8.38 |  | 16.52 | 18.92 | 14.46 |
| High-income North America | 13.79 | 13.77 | 13.81 |  | 15.31 | 16.51 | 14.13 |
| Oceania | 4.16 | 4.83 | 3.62 |  | 4.41 | 5.15 | 3.79 |
| Central Sub-Saharan Africa | 4.74 | 6.11 | 3.54 |  | 5.54 | 7.07 | 4.31 |
| Eastern Sub-Saharan Africa | 2.99 | 4.23 | 1.98 |  | 4.36 | 6.1 | 3.2 |
| Southern Sub-Saharan Africa | 6.92 | 8.46 | 5.32 |  | 7.78 | 10.82 | 5.59 |
| Western Sub-Saharan Africa | 6.4 | 6.07 | 6.72 |  | 8.38 | 7.8 | 8.89 |
| High body-mass index |  |  |  |  |  |  |  |
| Global | 2.21 | 2.91 | 1.63 |  | 7.26 | 8.52 | 6.4 |
| Socio-demographic index |  |  |  |  |  |  |  |
| High | 6.13 | 6.61 | 5.75 |  | 15.3 | 16.05 | 14.74 |
| High-middle | 2.79 | 3.32 | 2.44 |  | 9.87 | 10.89 | 9.35 |
| Middle | 1.68 | 2.38 | 1.11 |  | 7.59 | 9.05 | 6.72 |
| Low-middle | 1.5 | 2.59 | 0.48 |  | 5.79 | 7.67 | 4.36 |
| Low | 0.57 | 1.38 | 0 |  | 3.63 | 4.73 | 2.69 |
| GBD regions |  |  |  |  |  |  |  |
| High-income Asia Pacific | 0.31 | 0 | 0.58 |  | 3.51 | 2.09 | 4.48 |
| Central Asia | 5.32 | 5.69 | 4.98 |  | 10.14 | 11.57 | 9.14 |
| East Asia | 0 | 0 | 0 |  | 6.68 | 6.23 | 6.87 |
| South Asia | 0.19 | 0.54 | 0 |  | 3.67 | 4.51 | 3.01 |
| Southeast Asia | 0.54 | 0.99 | 0.1 |  | 4.49 | 6.31 | 3.33 |
| Australasia | 8.64 | 9.61 | 7.77 |  | 16.33 | 17.04 | 15.68 |
| Caribbean | 4.63 | 5.3 | 3.71 |  | 9.42 | 10.57 | 7.86 |
| Central Europe | 6.75 | 5.5 | 7.68 |  | 12.07 | 12.44 | 11.8 |
| Eastern Europe | 7.24 | 8.86 | 6.21 |  | 12.81 | 14.69 | 11.79 |
| Western Europe | 5.68 | 5.31 | 5.97 |  | 11.43 | 12.1 | 10.82 |
| Andean Latin America | 4.41 | 5.23 | 3.63 |  | 11.43 | 12.34 | 10.7 |
| Central Latin America | 6.89 | 7.95 | 5.61 |  | 14.49 | 15.03 | 14 |
| Southern Latin America | 6.62 | 7.24 | 6.03 |  | 13.62 | 14.33 | 12.99 |
| Tropical Latin America | 5.57 | 6.16 | 5.02 |  | 13.06 | 13.57 | 12.56 |
| North Africa and Middle East | 6.79 | 9.29 | 4.39 |  | 16.18 | 17.97 | 14.64 |
| High-income North America | 12.63 | 13.43 | 11.81 |  | 19.43 | 20.11 | 18.76 |
| Oceania | 5.38 | 6.41 | 4.56 |  | 8.56 | 9.25 | 8.02 |
| Central Sub-Saharan Africa | -0.08 | 0.45 | 0 |  | 3.58 | 4.62 | 2.72 |
| Eastern Sub-Saharan Africa | 0.09 | 0.87 | 0 |  | 2.78 | 4.28 | 1.79 |
| Southern Sub-Saharan Africa | 7.31 | 11.47 | 3 |  | 11.4 | 17.21 | 7.21 |
| Western Sub-Saharan Africa | 1.98 | 3.07 | 0.98 |  | 5.82 | 6.97 | 4.84 |
| Kidney dysfunction |  |  |  |  |  |  |  |
| Global | 4.77 | 4.9 | 4.65 |  | 5.47 | 5.43 | 5.49 |
| Socio-demographic index |  |  |  |  |  |  |  |
| High | 3.14 | 3.2 | 3.09 |  | 3.91 | 3.88 | 3.92 |
| High-middle | 4.54 | 4.72 | 4.41 |  | 5.41 | 5.48 | 5.37 |
| Middle | 4.97 | 5.07 | 4.89 |  | 5.96 | 5.92 | 5.98 |
| Low-middle | 5.36 | 5.44 | 5.28 |  | 5.65 | 5.64 | 5.66 |
| Low | 4.67 | 4.91 | 4.43 |  | 4.68 | 4.72 | 4.66 |
| GBD regions |  |  |  |  |  |  |  |
| High-income Asia Pacific | 3.3 | 3.51 | 3.16 |  | 3.2 | 3.32 | 3.12 |
| Central Asia | 7.05 | 7.41 | 6.72 |  | 7.4 | 7.7 | 7.19 |
| East Asia | 4.04 | 4.27 | 3.88 |  | 4.93 | 5.01 | 4.9 |
| South Asia | 5.46 | 5.41 | 5.5 |  | 5.75 | 5.68 | 5.8 |
| Southeast Asia | 6.87 | 6.9 | 6.83 |  | 7.42 | 7.43 | 7.42 |
| Australasia | 1.83 | 2.09 | 1.6 |  | 2.17 | 2.48 | 1.88 |
| Caribbean | 3.6 | 3.4 | 3.89 |  | 3.89 | 3.53 | 4.38 |
| Central Europe | 3.65 | 3.78 | 3.55 |  | 3.38 | 3.6 | 3.22 |
| Eastern Europe | 6.09 | 6.46 | 5.85 |  | 6.53 | 6.75 | 6.42 |
| Western Europe | 2.3 | 2.29 | 2.31 |  | 2.29 | 2.36 | 2.23 |
| Andean Latin America | 2.4 | 2.49 | 2.31 |  | 2.26 | 2.12 | 2.37 |
| Central Latin America | 3.84 | 3.85 | 3.84 |  | 3.84 | 3.69 | 3.98 |
| Southern Latin America | 2.58 | 2.66 | 2.5 |  | 2.61 | 2.72 | 2.51 |
| Tropical Latin America | 3.72 | 3.36 | 4.06 |  | 3.09 | 2.69 | 3.49 |
| North Africa and Middle East | 5.05 | 4.97 | 5.13 |  | 5.64 | 5.46 | 5.79 |
| High-income North America | 2.99 | 3.09 | 2.88 |  | 3.35 | 3.63 | 3.07 |
| Oceania | 4.9 | 5.05 | 4.76 |  | 5.2 | 5.26 | 5.15 |
| Central Sub-Saharan Africa | 5.22 | 5.39 | 5.06 |  | 5.34 | 5.27 | 5.4 |
| Eastern Sub-Saharan Africa | 4.02 | 4.45 | 3.66 |  | 4.04 | 4.27 | 3.89 |
| Southern Sub-Saharan Africa | 6.98 | 7.4 | 6.55 |  | 6.5 | 7.2 | 5.99 |
| Western Sub-Saharan Africa | 5.13 | 5.29 | 4.96 |  | 5.11 | 5.06 | 5.16 |

**Supplementary Table S5. DALYs of stroke attributable to metabolic risk factors among young adults in 1990 and 2021, and estimated annual percentage changes from 1990 to 2021, by country and territories.**

| **Countries** | Number of cases in 1990 | Age-standardized rate per 100000 population (95% UI) in 1990 | Number of cases in 2021 | Age-standardized rate per 100000 population (95% UI) in 2021 | Estimated annual percentage changes (95% CI) from 1990 to 2021 |
| --- | --- | --- | --- | --- | --- |
| Afghanistan | 7505 | 511.18 (499.31 to 523.29) | 28403 | 408.49 (403.59 to 413.43) | -0.78 (-0.92 to -0.63) |
| Albania | 1968 | 194.98 (186.38 to 203.9) | 1059 | 142.09 (133.65 to 150.94) | -1.03 (-1.35 to -0.71) |
| Algeria | 18005 | 292.32 (288.01 to 296.68) | 23829 | 163.32 (161.25 to 165.41) | -2.24 (-2.44 to -2.05) |
| American Samoa | 70 | 510.94 (397.04 to 648.51) | 61 | 499.05 (382.06 to 641.07) | -0.26 (-0.38 to -0.14) |
| Andorra | 15 | 68.25 (37.9 to 114.69) | 9 | 37.73 (16.87 to 74.84) | -1.96 (-2.06 to -1.87) |
| Angola | 5892 | 235.07 (229.07 to 241.18) | 12723 | 166.76 (163.86 to 169.71) | -1.12 (-1.27 to -0.97) |
| Antigua and Barbuda | 32 | 173.36 (118.17 to 246.09) | 18 | 65.49 (39.01 to 103.32) | -2.14 (-2.53 to -1.74) |
| Argentina | 15710 | 173.77 (171.06 to 176.51) | 13693 | 98.58 (96.94 to 100.25) | -1.66 (-1.82 to -1.5) |
| Armenia | 1911 | 170.39 (162.78 to 178.27) | 859 | 85.36 (79.72 to 91.33) | -2.77 (-3.16 to -2.37) |
| Australia | 3533 | 65.09 (62.96 to 67.27) | 2934 | 39.01 (37.61 to 40.45) | -2.07 (-2.26 to -1.88) |
| Austria | 2561 | 109.24 (105.03 to 113.58) | 1019 | 41.18 (38.69 to 43.8) | -3.3 (-3.57 to -3.03) |
| Azerbaijan | 5113 | 231.82 (225.4 to 238.39) | 4225 | 111.77 (108.42 to 115.2) | -3.09 (-3.37 to -2.82) |
| Bahamas | 159 | 196 (166.42 to 229.51) | 171 | 143.24 (122.57 to 166.43) | -1.6 (-1.82 to -1.38) |
| Bahrain | 374 | 165.66 (149.17 to 183.59) | 815 | 131.07 (122.2 to 140.45) | -1.36 (-1.75 to -0.98) |
| Bangladesh | 92346 | 350.82 (348.54 to 353.11) | 147311 | 289.07 (287.6 to 290.56) | -0.5 (-0.87 to -0.13) |
| Barbados | 124 | 148.4 (123.4 to 177.05) | 84 | 103.62 (82.65 to 128.37) | -1.27 (-1.54 to -1.01) |
| Belarus | 7133 | 216.92 (211.91 to 222.02) | 5377 | 181.68 (176.81 to 186.67) | -1.21 (-1.83 to -0.58) |
| Belgium | 3312 | 107.38 (103.75 to 111.11) | 1247 | 41.73 (39.44 to 44.11) | -2.71 (-2.93 to -2.48) |
| Belize | 56 | 121.39 (91.29 to 158.81) | 131 | 98.29 (82.11 to 116.79) | -0.88 (-1.29 to -0.47) |
| Benin | 1976 | 182.79 (174.74 to 191.12) | 6077 | 187.57 (182.83 to 192.4) | 0.07 (-0.17 to 0.32) |
| Bermuda | 16 | 71.93 (41.23 to 117.97) | 6 | 36.67 (13.01 to 85.54) | -2.32 (-2.57 to -2.07) |
| Bhutan | 205 | 134.04 (116.11 to 154.05) | 237 | 87.7 (76.88 to 99.64) | -1.73 (-1.86 to -1.6) |
| Bolivia (Plurinational State of) | 3304 | 202.18 (195.31 to 209.23) | 4066 | 110.04 (106.67 to 113.48) | -2.26 (-2.63 to -1.89) |
| Bosnia and Herzegovina | 3067 | 206.37 (199.13 to 213.81) | 1127 | 128.72 (121.29 to 136.51) | -1.79 (-1.96 to -1.63) |
| Botswana | 673 | 220.17 (203.71 to 237.65) | 1087 | 126.14 (118.75 to 133.88) | -2.04 (-2.29 to -1.78) |
| Brazil | 136562 | 320.33 (318.63 to 322.04) | 89204 | 125.51 (124.69 to 126.34) | -3.29 (-3.52 to -3.06) |
| Brunei Darussalam | 249 | 266.07 (233.87 to 301.59) | 262 | 149.86 (132.27 to 169.21) | -2.28 (-2.73 to -1.84) |
| Bulgaria | 8702 | 356.75 (349.27 to 364.34) | 4256 | 236.04 (228.92 to 243.34) | -1.96 (-2.17 to -1.75) |
| Burkina Faso | 3017 | 157.82 (152.2 to 163.59) | 7444 | 136.16 (133.07 to 139.3) | -0.5 (-0.65 to -0.35) |
| Burundi | 5307 | 393.83 (383.2 to 404.69) | 7497 | 214.17 (209.34 to 219.09) | -2.28 (-2.58 to -1.98) |
| Cabo Verde | 285 | 380.06 (335.78 to 428.96) | 352 | 174.95 (157.14 to 194.25) | -2.25 (-2.6 to -1.89) |
| Cambodia | 6304 | 265.04 (258.48 to 271.73) | 8475 | 152.22 (148.99 to 155.5) | -2.13 (-2.28 to -1.98) |
| Cameroon | 4337 | 186.21 (180.65 to 191.89) | 23757 | 280.57 (277 to 284.19) | 1.4 (0.65 to 2.16) |
| Canada | 6768 | 70.9 (69.22 to 72.61) | 5211 | 51.13 (49.74 to 52.54) | -1.29 (-1.67 to -0.9) |
| Central African Republic | 1823 | 284.79 (271.71 to 298.37) | 3439 | 251.9 (243.52 to 260.49) | -0.57 (-0.68 to -0.47) |
| Chad | 2778 | 213.6 (205.69 to 221.74) | 8085 | 223.83 (218.93 to 228.82) | 0.13 (-0.03 to 0.3) |
| Chile | 5647 | 138.23 (134.63 to 141.91) | 4318 | 73.91 (71.72 to 76.15) | -1.88 (-1.97 to -1.78) |
| China | 764863 | 201.41 (200.96 to 201.87) | 806452 | 185.65 (185.24 to 186.05) | -0.34 (-0.48 to -0.19) |
| Colombia | 11147 | 118.75 (116.54 to 121) | 10320 | 66.27 (64.99 to 67.56) | -1.48 (-1.95 to -1.01) |
| Comoros | 301 | 300.94 (267.59 to 337.43) | 403 | 184.33 (166.72 to 203.32) | -2.13 (-2.63 to -1.62) |
| Congo | 1706 | 308.22 (293.63 to 323.38) | 3204 | 203.92 (196.91 to 211.11) | -1.78 (-2.04 to -1.53) |
| Cook Islands | 19 | 360.15 (215.75 to 567.52) | 12 | 282.94 (147.15 to 494.19) | -0.49 (-0.77 to -0.21) |
| Costa Rica | 796 | 89.07 (82.94 to 95.55) | 1066 | 68.25 (64.21 to 72.47) | -1.19 (-1.54 to -0.83) |
| Côte d'Ivoire | 8803 | 295.5 (289.26 to 301.84) | 21201 | 272.22 (268.56 to 275.92) | -0.2 (-0.38 to -0.01) |
| Croatia | 2837 | 183.7 (176.99 to 190.6) | 703 | 63.41 (58.79 to 68.31) | -3.52 (-3.65 to -3.39) |
| Cuba | 4337 | 134.16 (130.15 to 138.26) | 2070 | 68.77 (65.84 to 71.81) | -1.98 (-2.25 to -1.72) |
| Cyprus | 208 | 86.53 (75.14 to 99.17) | 187 | 38.46 (33.11 to 44.57) | -3.36 (-3.8 to -2.93) |
| Czechia | 5142 | 167.33 (162.75 to 172) | 1842 | 66.83 (63.79 to 70) | -2.67 (-2.91 to -2.44) |
| Democratic People's Republic of Korea | 18627 | 321.51 (316.88 to 326.2) | 26243 | 312.19 (308.42 to 315.99) | -0.56 (-0.75 to -0.37) |
| Democratic Republic of the Congo | 17883 | 203.24 (200.24 to 206.27) | 31853 | 140.29 (138.74 to 141.85) | -1.41 (-1.5 to -1.32) |
| Denmark | 1677 | 110.2 (104.98 to 115.61) | 583 | 40.04 (36.85 to 43.44) | -3.76 (-4.02 to -3.5) |
| Djibouti | 223 | 217.79 (189.9 to 248.75) | 719 | 171.75 (159.42 to 184.78) | -0.92 (-1.1 to -0.75) |
| Dominica | 20 | 106.7 (64.57 to 166.74) | 22 | 110.72 (69.46 to 167.57) | 0.13 (-0.11 to 0.37) |
| Dominican Republic | 3653 | 189.56 (183.4 to 195.88) | 6169 | 178.72 (174.28 to 183.24) | 0.53 (0.22 to 0.83) |
| Ecuador | 4357 | 162.07 (157.26 to 167) | 5378 | 97.52 (94.92 to 100.17) | -1.41 (-1.7 to -1.11) |
| Egypt | 51951 | 352.27 (349.23 to 355.33) | 83424 | 264.07 (262.28 to 265.87) | -0.42 (-0.73 to -0.11) |
| El Salvador | 2567 | 197.3 (189.66 to 205.18) | 1964 | 106.06 (101.38 to 110.91) | -1.94 (-2.5 to -1.36) |
| Equatorial Guinea | 262 | 280.96 (247.87 to 317.29) | 632 | 143.03 (131.97 to 154.79) | -2.64 (-2.97 to -2.32) |
| Eritrea | 2305 | 299.39 (287.2 to 311.97) | 4210 | 222.35 (215.65 to 229.2) | -0.99 (-1.11 to -0.87) |
| Estonia | 755 | 158.66 (147.53 to 170.43) | 227 | 60.9 (53.18 to 69.59) | -4.2 (-4.58 to -3.81) |
| Eswatini | 281 | 163.36 (144.61 to 183.93) | 740 | 208.19 (193.4 to 223.84) | 1.26 (0.46 to 2.06) |
| Ethiopia | 14477 | 129.26 (127.15 to 131.39) | 31232 | 109.54 (108.32 to 110.77) | -0.65 (-0.9 to -0.4) |
| Fiji | 1100 | 483.47 (455.14 to 513.16) | 1150 | 414.89 (391.24 to 439.62) | -0.64 (-0.78 to -0.5) |
| Finland | 3061 | 191.29 (184.56 to 198.22) | 864 | 59.95 (56.01 to 64.1) | -3.55 (-3.87 to -3.24) |
| France | 16473 | 92.48 (91.07 to 93.9) | 6548 | 40.58 (39.6 to 41.58) | -2.71 (-2.91 to -2.52) |
| Gabon | 490 | 200.06 (182.62 to 218.76) | 726 | 143.52 (133.22 to 154.42) | -1.29 (-1.44 to -1.15) |
| Gambia | 656 | 281.87 (260.31 to 304.82) | 1918 | 303.66 (290.12 to 317.7) | 0.01 (-0.24 to 0.25) |
| Georgia | 6109 | 362.7 (353.64 to 371.93) | 2252 | 219.87 (210.86 to 229.2) | -2.59 (-3.57 to -1.6) |
| Germany | 39326 | 158.07 (156.51 to 159.64) | 10488 | 46.68 (45.79 to 47.58) | -3.95 (-4.32 to -3.59) |
| Ghana | 18008 | 489.63 (482.45 to 496.89) | 32518 | 328.26 (324.68 to 331.86) | -1.26 (-1.45 to -1.07) |
| Greece | 4024 | 137.59 (133.37 to 141.91) | 1473 | 59.93 (56.88 to 63.11) | -2.96 (-3.19 to -2.72) |
| Greenland | 58 | 276 (208.75 to 358.73) | 18 | 103.04 (60.6 to 164.9) | -3.47 (-3.66 to -3.29) |
| Grenada | 68 | 308.85 (239.57 to 392.55) | 38 | 130.74 (92.17 to 180.38) | -2.71 (-3.01 to -2.41) |
| Guam | 92 | 192 (154.65 to 235.88) | 120 | 287.25 (238.13 to 343.62) | 1.43 (1.18 to 1.68) |
| Guatemala | 2669 | 142.72 (137.32 to 148.27) | 6942 | 152.24 (148.65 to 155.89) | -0.15 (-0.64 to 0.34) |
| Guinea | 3492 | 250.97 (242.68 to 259.48) | 9459 | 284.76 (279.01 to 290.61) | 0.76 (0.61 to 0.9) |
| Guinea-Bissau | 1113 | 488.64 (460.12 to 518.5) | 2385 | 427.92 (410.81 to 445.59) | -0.36 (-0.42 to -0.29) |
| Guyana | 750 | 342.12 (317.87 to 367.79) | 581 | 269.1 (247.43 to 292.21) | -0.74 (-1 to -0.49) |
| Haiti | 8100 | 496.43 (485.64 to 507.39) | 16870 | 413.01 (406.8 to 419.3) | -0.22 (-0.34 to -0.09) |
| Honduras | 2996 | 277.31 (267.41 to 287.5) | 3999 | 135.38 (131.19 to 139.68) | -2.97 (-3.31 to -2.63) |
| Hungary | 10593 | 327.05 (320.8 to 333.4) | 2243 | 92.47 (88.68 to 96.4) | -4.71 (-5.06 to -4.36) |
| Iceland | 57 | 69.32 (52.42 to 89.99) | 39 | 38.74 (27.51 to 53.16) | -1.54 (-1.79 to -1.3) |
| India | 363956 | 156.41 (155.9 to 156.92) | 492689 | 108.71 (108.4 to 109.01) | -1.2 (-1.49 to -0.92) |
| Indonesia | 260333 | 503.48 (501.54 to 505.43) | 343829 | 380.85 (379.58 to 382.13) | -0.78 (-0.95 to -0.61) |
| Iran (Islamic Republic of) | 25886 | 191.69 (189.34 to 194.07) | 41989 | 130.07 (128.81 to 131.34) | -1.14 (-1.24 to -1.05) |
| Iraq | 20942 | 476.22 (469.72 to 482.8) | 34411 | 278.19 (275.25 to 281.17) | -1.87 (-2.12 to -1.61) |
| Ireland | 1072 | 106.42 (100.15 to 112.99) | 496 | 37.82 (34.54 to 41.35) | -3.55 (-3.8 to -3.3) |
| Israel | 1219 | 86.49 (81.7 to 91.49) | 785 | 30.83 (28.71 to 33.07) | -3.52 (-3.75 to -3.3) |
| Italy | 16827 | 103.25 (101.69 to 104.82) | 5267 | 38.66 (37.62 to 39.72) | -3.33 (-3.51 to -3.14) |
| Jamaica | 786 | 125.49 (116.76 to 134.72) | 1075 | 117.75 (110.8 to 125.03) | -0.6 (-1.11 to -0.09) |
| Japan | 44361 | 125.3 (124.13 to 126.48) | 19707 | 68.88 (67.91 to 69.85) | -2.16 (-2.34 to -1.98) |
| Jordan | 2355 | 276.03 (264.71 to 287.74) | 5241 | 135.38 (131.73 to 139.11) | -2.77 (-2.98 to -2.55) |
| Kazakhstan | 17524 | 336.36 (331.39 to 341.4) | 12537 | 206.23 (202.63 to 209.88) | -2.25 (-3.06 to -1.42) |
| Kenya | 7001 | 140.18 (136.86 to 143.56) | 18363 | 131.1 (129.2 to 133.02) | -0.07 (-0.35 to 0.22) |
| Kiribati | 157 | 760.04 (644.53 to 890.97) | 334 | 910.42 (815.08 to 1014.03) | 0.42 (0.28 to 0.57) |
| Kuwait | 908 | 126.42 (118.31 to 134.95) | 2182 | 102.55 (98.19 to 107.09) | -0.89 (-1.5 to -0.27) |
| Kyrgyzstan | 3969 | 315.63 (305.8 to 325.72) | 3903 | 186 (180.19 to 191.95) | -3.24 (-3.75 to -2.73) |
| Lao People's Democratic Republic | 5569 | 570.61 (555.62 to 585.91) | 8698 | 366.62 (358.93 to 374.43) | -1.58 (-1.66 to -1.5) |
| Latvia | 1523 | 195.69 (185.98 to 205.78) | 518 | 101.08 (92.52 to 110.35) | -3.16 (-3.6 to -2.72) |
| Lebanon | 2480 | 317.42 (304.96 to 330.29) | 2982 | 145.44 (140.24 to 150.79) | -2.02 (-2.59 to -1.45) |
| Lesotho | 380 | 109.55 (98.79 to 121.17) | 1329 | 239.68 (226.91 to 252.98) | 3.69 (3.05 to 4.34) |
| Liberia | 1735 | 279.69 (266.6 to 293.27) | 4245 | 281.21 (272.79 to 289.83) | 0.22 (0.06 to 0.39) |
| Libya | 2723 | 266.14 (256.14 to 276.45) | 7610 | 307.08 (300.2 to 314.08) | 0.7 (0.48 to 0.92) |
| Lithuania | 2035 | 187.02 (178.97 to 195.35) | 677 | 96.26 (89.15 to 103.82) | -2.04 (-2.46 to -1.62) |
| Luxembourg | 184 | 143.85 (123.83 to 166.28) | 62 | 31.13 (23.88 to 40.08) | -5.58 (-5.83 to -5.33) |
| Madagascar | 14812 | 527.51 (518.96 to 536.17) | 32712 | 444.28 (439.45 to 449.16) | -0.48 (-0.56 to -0.41) |
| Malawi | 5517 | 249.47 (242.85 to 256.24) | 11132 | 229.34 (225.08 to 233.67) | -0.57 (-0.91 to -0.22) |
| Malaysia | 14821 | 284.42 (279.84 to 289.06) | 27178 | 244.92 (242.01 to 247.85) | -0.14 (-0.48 to 0.2) |
| Maldives | 229 | 511.58 (446.12 to 584.37) | 433 | 162.63 (147.58 to 179.17) | -3.81 (-4.09 to -3.53) |
| Mali | 4778 | 250.55 (243.46 to 257.79) | 10034 | 193.93 (190.11 to 197.81) | -0.79 (-0.99 to -0.58) |
| Malta | 126 | 108.26 (90.18 to 129.13) | 53 | 43.14 (32.33 to 56.79) | -2.77 (-2.95 to -2.6) |
| Marshall Islands | 66 | 587.97 (454.42 to 749.59) | 133 | 754.67 (631.88 to 894.64) | 0.76 (0.59 to 0.92) |
| Mauritania | 1599 | 323.68 (307.91 to 340.06) | 1941 | 178.79 (170.86 to 187) | -1.97 (-2.1 to -1.84) |
| Mauritius | 1414 | 381.94 (362.24 to 402.45) | 1189 | 328.2 (309.8 to 347.42) | 0.45 (0.11 to 0.79) |
| Mexico | 28972 | 128.75 (127.26 to 130.25) | 39165 | 98.88 (97.9 to 99.86) | -0.9 (-1.24 to -0.57) |
| Micronesia (Federated States of) | 196 | 744.8 (643.85 to 857.43) | 215 | 736.45 (640.72 to 842.72) | -0.03 (-0.09 to 0.02) |
| Monaco | 11 | 141.41 (71.68 to 252.54) | 6 | 82.04 (31.28 to 176.93) | -1.79 (-1.85 to -1.72) |
| Mongolia | 1590 | 301.74 (286.73 to 317.37) | 2683 | 247.23 (237.96 to 256.8) | -0.83 (-1.35 to -0.31) |
| Montenegro | 558 | 286.34 (263.07 to 311.12) | 280 | 161.48 (143.07 to 181.67) | -2.1 (-2.49 to -1.7) |
| Morocco | 27304 | 399.66 (394.9 to 404.47) | 24697 | 214.95 (212.28 to 217.65) | -2.26 (-2.5 to -2.01) |
| Mozambique | 7713 | 254.23 (248.57 to 259.98) | 26506 | 380.3 (375.69 to 384.96) | 1.9 (1.66 to 2.14) |
| Myanmar | 58281 | 520.52 (516.27 to 524.79) | 63528 | 386.02 (383.02 to 389.04) | -0.71 (-1 to -0.43) |
| Namibia | 547 | 166.65 (152.82 to 181.43) | 1053 | 143.73 (135.14 to 152.74) | -0.91 (-1.39 to -0.42) |
| Nauru | 36 | 1272.47 (892.23 to 1763.61) | 48 | 1455.81 (1072.28 to 1934.8) | 0.23 (-0.28 to 0.73) |
| Nepal | 8954 | 188.28 (184.39 to 192.25) | 8336 | 92.48 (90.5 to 94.5) | -2.52 (-2.66 to -2.39) |
| Netherlands | 4441 | 91.63 (88.96 to 94.37) | 1619 | 37.77 (35.96 to 39.66) | -2.99 (-3.16 to -2.83) |
| New Zealand | 869 | 81.36 (76.04 to 86.96) | 653 | 43.99 (40.67 to 47.5) | -2.25 (-2.39 to -2.12) |
| Nicaragua | 1454 | 164.41 (155.99 to 173.19) | 2063 | 96.91 (92.77 to 101.2) | -1.61 (-1.8 to -1.42) |
| Niger | 3377 | 198.08 (191.39 to 204.97) | 8499 | 173.15 (169.43 to 176.92) | -0.38 (-0.47 to -0.28) |
| Nigeria | 30527 | 145.21 (143.57 to 146.86) | 75266 | 138.26 (137.27 to 139.26) | -0.05 (-0.21 to 0.11) |
| Niue | 3 | 528.91 (105.94 to 1578.61) | 2 | 526.41 (73.6 to 1838.89) | -0.8 (-1.02 to -0.57) |
| North Macedonia | 1926 | 315.71 (301.76 to 330.14) | 1049 | 150.27 (141.3 to 159.72) | -2.37 (-2.58 to -2.16) |
| Northern Mariana Islands | 76 | 397.06 (312.53 to 497.65) | 40 | 314.01 (224.56 to 428.53) | -1.02 (-1.29 to -0.75) |
| Norway | 1278 | 100.5 (95.06 to 106.17) | 503 | 33.54 (30.67 to 36.61) | -3.73 (-3.85 to -3.61) |
| Oman | 1252 | 186.45 (176.22 to 197.14) | 3488 | 152.02 (146.95 to 157.25) | -0.03 (-0.27 to 0.22) |
| Pakistan | 38154 | 155.4 (153.83 to 156.98) | 145023 | 211.3 (210.2 to 212.39) | 0.9 (0.76 to 1.04) |
| Palau | 31 | 611.87 (415.23 to 871.35) | 42 | 849.16 (610.34 to 1153.36) | 1.38 (1.2 to 1.55) |
| Palestine | 1090 | 244.75 (230.05 to 260.21) | 2179 | 144.55 (138.46 to 150.86) | -1.73 (-2 to -1.47) |
| Panama | 838 | 124.32 (115.99 to 133.11) | 1172 | 95.34 (89.95 to 100.97) | -0.93 (-1.23 to -0.62) |
| Papua New Guinea | 2010 | 190.43 (182.11 to 199.03) | 7847 | 257.99 (252.29 to 263.79) | 1.05 (0.71 to 1.38) |
| Paraguay | 2085 | 198.18 (189.72 to 206.92) | 2464 | 107.17 (102.97 to 111.49) | -1.9 (-2.02 to -1.78) |
| Peru | 6807 | 117.04 (114.25 to 119.88) | 14103 | 121.39 (119.39 to 123.41) | 0.6 (0.15 to 1.04) |
| Philippines | 47003 | 282.77 (280.2 to 285.35) | 101600 | 300.87 (299.01 to 302.73) | 0.79 (0.51 to 1.07) |
| Poland | 28167 | 219.33 (216.77 to 221.92) | 11728 | 98.95 (97.14 to 100.78) | -2.54 (-2.66 to -2.42) |
| Portugal | 6428 | 226.53 (221.02 to 232.14) | 1410 | 55.96 (53.06 to 58.98) | -5.36 (-5.73 to -4.98) |
| Puerto Rico | 1050 | 101.08 (95.05 to 107.39) | 483 | 58.7 (53.58 to 64.18) | -2.14 (-2.41 to -1.87) |
| Qatar | 407 | 179.15 (162.09 to 197.8) | 1497 | 84.34 (79.99 to 88.94) | -2.99 (-3.33 to -2.66) |
| Republic of Korea | 37442 | 245.47 (242.97 to 247.98) | 8348 | 57.69 (56.45 to 58.94) | -5.78 (-6.3 to -5.26) |
| Republic of Moldova | 3208 | 216.69 (209.26 to 224.34) | 1575 | 126.57 (120.34 to 133.1) | -2.12 (-2.45 to -1.79) |
| Romania | 15902 | 233.86 (230.22 to 237.55) | 6640 | 140.45 (137.07 to 143.9) | -2.34 (-2.71 to -1.96) |
| Russian Federation | 121713 | 239.3 (237.96 to 240.65) | 109770 | 235.54 (234.13 to 236.96) | -0.54 (-1.08 to 0) |
| Rwanda | 8273 | 469.63 (459.5 to 479.93) | 5649 | 147.38 (143.55 to 151.28) | -4.99 (-5.55 to -4.44) |
| Saint Kitts and Nevis | 59 | 500.25 (379.65 to 648.27) | 21 | 110.34 (68.34 to 169.29) | -5.43 (-6.13 to -4.72) |
| Saint Lucia | 83 | 245.7 (195.04 to 305.92) | 66 | 120.82 (93.36 to 153.94) | -2.46 (-2.73 to -2.18) |
| Saint Vincent and the Grenadines | 62 | 220.58 (168.23 to 284.84) | 46 | 144.01 (105.42 to 192.42) | -1.98 (-2.26 to -1.7) |
| Samoa | 170 | 431.2 (367.68 to 502.98) | 299 | 545.64 (485.24 to 611.7) | 0.79 (0.66 to 0.91) |
| San Marino | 5 | 76.52 (26.29 to 173.99) | 3 | 38.41 (7.15 to 119.84) | -2.09 (-2.28 to -1.9) |
| Sao Tome and Principe | 80 | 336.97 (266.02 to 421.77) | 185 | 297.57 (256.15 to 343.87) | -0.59 (-1.05 to -0.13) |
| Saudi Arabia | 13029 | 288.95 (283.96 to 294) | 44294 | 253.19 (250.83 to 255.58) | -0.13 (-0.32 to 0.06) |
| Senegal | 5592 | 334.12 (325.32 to 343.11) | 9041 | 220.69 (216.13 to 225.33) | -1.17 (-1.35 to -0.98) |
| Serbia | 8579 | 294.44 (288.24 to 300.74) | 3174 | 122.51 (118.28 to 126.87) | -2.88 (-2.97 to -2.78) |
| Seychelles | 78 | 393.84 (310.28 to 493.83) | 71 | 215.43 (168.07 to 272.65) | -1.89 (-2.13 to -1.64) |
| Sierra Leone | 3244 | 307.99 (297.39 to 318.88) | 7781 | 325.78 (318.5 to 333.18) | 0.55 (0.31 to 0.79) |
| Singapore | 1092 | 92.44 (87.02 to 98.1) | 754 | 37.36 (34.7 to 40.21) | -3.51 (-3.77 to -3.26) |
| Slovakia | 3391 | 198.06 (191.44 to 204.86) | 1499 | 92.37 (87.72 to 97.23) | -2.31 (-2.44 to -2.18) |
| Slovenia | 782 | 120.72 (112.39 to 129.51) | 212 | 40.08 (34.81 to 46.01) | -4.03 (-4.2 to -3.85) |
| Solomon Islands | 212 | 294.27 (255.61 to 337.27) | 655 | 356.29 (329.46 to 384.75) | 0.58 (0.5 to 0.66) |
| Somalia | 5161 | 268.43 (261.15 to 275.86) | 10282 | 211.19 (207.1 to 215.34) | -0.76 (-0.92 to -0.59) |
| South Africa | 45483 | 440.4 (436.33 to 444.5) | 40447 | 201.57 (199.61 to 203.54) | -2.97 (-3.9 to -2.03) |
| South Sudan | 2550 | 191.99 (184.5 to 199.71) | 3533 | 168.25 (162.75 to 173.89) | -0.56 (-0.92 to -0.21) |
| Spain | 11733 | 107.17 (105.23 to 109.13) | 4580 | 41.94 (40.72 to 43.19) | -3.73 (-3.98 to -3.47) |
| Sri Lanka | 13382 | 250.47 (246.23 to 254.76) | 10959 | 174.42 (171.16 to 177.72) | -1.44 (-1.7 to -1.18) |
| Sudan | 25810 | 544.15 (537.48 to 550.89) | 41115 | 331.3 (328.08 to 334.54) | -1.63 (-1.73 to -1.54) |
| Suriname | 244 | 243.51 (213.46 to 276.75) | 298 | 180.16 (160.28 to 201.85) | -1.2 (-1.52 to -0.88) |
| Sweden | 1812 | 75.96 (72.5 to 79.54) | 963 | 34.54 (32.39 to 36.8) | -2.51 (-2.67 to -2.36) |
| Switzerland | 1651 | 73.98 (70.45 to 77.64) | 555 | 22.13 (20.32 to 24.06) | -4.56 (-4.86 to -4.25) |
| Syrian Arab Republic | 16250 | 562.46 (553.71 to 571.33) | 8742 | 287.84 (281.43 to 294.38) | -2.23 (-2.54 to -1.91) |
| Taiwan (Province of China) | 12617 | 173.55 (170.53 to 176.61) | 6385 | 92.76 (90.49 to 95.08) | -1.86 (-2.2 to -1.52) |
| Tajikistan | 3917 | 296.49 (287.09 to 306.14) | 5527 | 175 (170.4 to 179.7) | -2.59 (-2.97 to -2.21) |
| Thailand | 31165 | 173.54 (171.61 to 175.48) | 53288 | 294.56 (292.05 to 297.09) | 1.02 (0.41 to 1.63) |
| Timor-Leste | 641 | 300.81 (277.76 to 325.34) | 1042 | 317.64 (298.36 to 337.89) | 0.33 (-0.11 to 0.78) |
| Togo | 2737 | 331.37 (318.92 to 344.19) | 5613 | 245.86 (239.45 to 252.4) | -1.13 (-1.34 to -0.91) |
| Tokelau | 2 | 450.66 (44.69 to 1807.2) | 2 | 559.05 (68.33 to 2058.4) | 0.11 (-0.09 to 0.31) |
| Tonga | 49 | 233.29 (171.87 to 310.44) | 69 | 262.39 (203.77 to 332.93) | 0.43 (0.37 to 0.5) |
| Trinidad and Tobago | 666 | 182.47 (168.8 to 196.97) | 827 | 184.5 (172.04 to 197.71) | -0.21 (-0.48 to 0.06) |
| Tunisia | 4064 | 181.65 (176.04 to 187.39) | 5379 | 143.32 (139.5 to 147.22) | -0.86 (-0.92 to -0.8) |
| Turkey | 50071 | 313.65 (310.89 to 316.42) | 29677 | 115.19 (113.88 to 116.5) | -3.53 (-3.68 to -3.39) |
| Turkmenistan | 2857 | 284.97 (274.48 to 295.78) | 6579 | 411.18 (401.29 to 421.26) | 1.31 (0.85 to 1.76) |
| Tuvalu | 17 | 636.18 (370.7 to 1020.92) | 25 | 709.61 (459.32 to 1050.31) | 0.36 (0.25 to 0.47) |
| Uganda | 7772 | 214.31 (209.49 to 219.21) | 15603 | 156.02 (153.55 to 158.52) | -2.1 (-2.52 to -1.67) |
| Ukraine | 31364 | 195.66 (193.5 to 197.84) | 34294 | 246.11 (243.48 to 248.76) | 0.18 (-0.22 to 0.59) |
| United Arab Emirates | 1749 | 194.59 (185.55 to 204.01) | 5240 | 123.27 (118.74 to 127.98) | -1.54 (-1.78 to -1.3) |
| United Kingdom | 18576 | 113.03 (111.4 to 114.67) | 8211 | 44.26 (43.31 to 45.23) | -3.28 (-3.44 to -3.12) |
| United Republic of Tanzania | 11578 | 202.42 (198.71 to 206.17) | 25978 | 176.28 (174.13 to 178.45) | -0.71 (-0.86 to -0.56) |
| United States of America | 81015 | 94.31 (93.66 to 94.96) | 73475 | 80.97 (80.39 to 81.56) | -0.46 (-0.63 to -0.29) |
| United States Virgin Islands | 47 | 154.71 (113.5 to 206.17) | 24 | 123.4 (78.67 to 185.68) | -0.62 (-0.89 to -0.35) |
| Uruguay | 1998 | 234.84 (224.65 to 245.38) | 1019 | 107.97 (101.44 to 114.81) | -2.63 (-2.92 to -2.33) |
| Uzbekistan | 14439 | 254.27 (250.09 to 258.5) | 22663 | 198.66 (196.08 to 201.26) | -0.51 (-1.14 to 0.13) |
| Vanuatu | 291 | 750.23 (665.94 to 842.51) | 707 | 826.63 (766.49 to 890.34) | 0.11 (0.02 to 0.21) |
| Venezuela (Bolivarian Republic of) | 11850 | 215.74 (211.86 to 219.68) | 10880 | 139.92 (137.29 to 142.59) | -2.02 (-2.61 to -1.44) |
| Viet Nam | 50192 | 273.87 (271.46 to 276.31) | 77140 | 226.48 (224.88 to 228.08) | -0.12 (-0.41 to 0.17) |
| Yemen | 9049 | 309.34 (302.97 to 315.81) | 22397 | 236.32 (233.23 to 239.44) | -1.04 (-1.21 to -0.87) |
| Zambia | 2864 | 176.24 (169.74 to 182.93) | 11094 | 217.02 (212.97 to 221.14) | 0.85 (0.72 to 0.97) |
| Zimbabwe | 1886 | 82.19 (78.49 to 86.03) | 8633 | 206.23 (201.89 to 210.64) | 3.87 (2.83 to 4.91) |

**Supplementary Table S6. Global disability-adjusted life years (DALYs) of ischemic stroke attributable to metabolic risk factors among young adults in 1990 and 2021, and estimated annual percentage changes from 1990 to 2021**

| Characteristics | 1990 | |  | 2021 | |  | 1990−2021 | |
| --- | --- | --- | --- | --- | --- | --- | --- | --- |
|  | Number of DALYs | Age-standardized rate per 100000 population (95% UI) |  | Number of DALYs | Age-standardized rate per 100000 population (95% UI) |  | Relative change (%) | Estimated annual percentage changes (95% CI) |
| Global | 877865 | 56.74 (56.86 to 56.62) |  | 1147521 | 48.81 (48.9 to 48.72) |  | 30.72 | -0.55 (-0.59 to -0.51) |
| Socio-demographic index |  |  |  |  |  |  |  |  |
| High | 123664 | 43.56 (43.8 to 43.32) |  | 111255 | 36 (36.21 to 35.79) |  | -10.03 | -0.58 (-0.72 to -0.43) |
| High-middle | 245498 | 71.79 (72.08 to 71.51) |  | 225999 | 56.05 (56.29 to 55.82) |  | -7.94 | -1.02 (-1.12 to -0.91) |
| Middle | 298392 | 59.26 (59.48 to 59.05) |  | 395986 | 51.75 (51.91 to 51.58) |  | 32.71 | -0.45 (-0.5 to -0.39) |
| Low-middle | 158741 | 53.21 (53.48 to 52.95) |  | 289033 | 49.5 (49.68 to 49.31) |  | 82.08 | -0.2 (-0.23 to -0.18) |
| Low | 50631 | 43.12 (43.5 to 42.74) |  | 124295 | 43.37 (43.61 to 43.13) |  | 145.49 | -0.01 (-0.08 to 0.07) |
| GBD regions |  |  |  |  |  |  |  |  |
| High-income Asia Pacific | 21023 | 40.58 (41.13 to 40.03) |  | 10229 | 22.85 (23.3 to 22.41) |  | -51.34 | -2.29 (-2.48 to -2.11) |
| Central Asia | 21594 | 106.92 (108.36 to 105.48) |  | 24811 | 79.46 (80.46 to 78.47) |  | 14.9 | -1.47 (-1.69 to -1.26) |
| East Asia | 227483 | 57.55 (57.79 to 57.31) |  | 257014 | 57.53 (57.75 to 57.3) |  | 12.98 | -0.04 (-0.12 to 0.04) |
| South Asia | 99971 | 34.37 (34.58 to 34.16) |  | 181990 | 31.12 (31.27 to 30.98) |  | 82.04 | -0.41 (-0.49 to -0.34) |
| Southeast Asia | 100500 | 75.58 (76.05 to 75.11) |  | 153117 | 69.76 (70.11 to 69.41) |  | 52.36 | -0.22 (-0.3 to -0.15) |
| Australasia | 1650 | 25.41 (26.66 to 24.2) |  | 1607 | 17.91 (18.81 to 17.04) |  | -2.61 | -1.32 (-1.42 to -1.23) |
| Caribbean | 4928 | 49.39 (50.8 to 48.01) |  | 6259 | 44.12 (45.23 to 43.04) |  | 27.01 | 0.01 (-0.16 to 0.19) |
| Central Europe | 28451 | 73.21 (74.06 to 72.35) |  | 13397 | 41.74 (42.46 to 41.03) |  | -52.91 | -1.98 (-2.09 to -1.87) |
| Eastern Europe | 69240 | 93.67 (94.37 to 92.97) |  | 53164 | 81.02 (81.72 to 80.32) |  | -23.22 | -0.93 (-1.27 to -0.58) |
| Western Europe | 43023 | 37.5 (37.85 to 37.14) |  | 18609 | 16.82 (17.06 to 16.58) |  | -56.75 | -2.75 (-2.97 to -2.54) |
| Andean Latin America | 4027 | 39.61 (40.86 to 38.38) |  | 4940 | 23.79 (24.46 to 23.13) |  | 22.67 | -1.76 (-2.01 to -1.52) |
| Central Latin America | 18859 | 42.31 (42.93 to 41.71) |  | 19718 | 25.48 (25.84 to 25.13) |  | 4.55 | -1.71 (-2 to -1.43) |
| Southern Latin America | 4823 | 34.33 (35.32 to 33.37) |  | 4407 | 21.29 (21.92 to 20.66) |  | -8.63 | -1.42 (-1.57 to -1.26) |
| Tropical Latin America | 24427 | 55.53 (56.23 to 54.83) |  | 18465 | 25.21 (25.58 to 24.85) |  | -24.41 | -2.83 (-3.38 to -2.27) |
| North Africa and Middle East | 109621 | 126.99 (127.75 to 126.23) |  | 203037 | 99.52 (99.95 to 99.09) |  | 85.22 | -0.72 (-0.76 to -0.68) |
| High-income North America | 38519 | 40.34 (40.75 to 39.94) |  | 37072 | 36.77 (37.15 to 36.4) |  | -3.76 | -0.4 (-0.5 to -0.3) |
| Oceania | 978 | 56.13 (59.81 to 52.64) |  | 2048 | 50.69 (52.95 to 48.51) |  | 109.41 | -0.42 (-0.49 to -0.35) |
| Central Sub-Saharan Africa | 5383 | 41.31 (42.44 to 40.2) |  | 11638 | 33.56 (34.18 to 32.95) |  | 116.2 | -0.77 (-0.81 to -0.72) |
| Eastern Sub-Saharan Africa | 16040 | 37.01 (37.6 to 36.44) |  | 38973 | 35.52 (35.88 to 35.16) |  | 142.97 | -0.21 (-0.25 to -0.18) |
| Southern Sub-Saharan Africa | 10674 | 76.64 (78.12 to 75.18) |  | 13075 | 48.82 (49.67 to 47.99) |  | 22.49 | -1.53 (-2.18 to -0.87) |
| Western Sub-Saharan Africa | 26651 | 58.98 (59.7 to 58.27) |  | 73951 | 61.51 (61.96 to 61.07) |  | 177.48 | 0.25 (0.17 to 0.33) |

**Supplementary Table S7. Global disability-adjusted life years (DALYs) of intracerebral hemorrhage attributable to metabolic risk factors among young adults in 1990 and 2021, and estimated annual percentage changes from 1990 to 2021**

| Characteristics | 1990 | |  | 2021 | |  | 1990−2021 | |
| --- | --- | --- | --- | --- | --- | --- | --- | --- |
|  | Number of DALYs | Age-standardized rate per 100000 population (95% UI) |  | Number of DALYs | Age-standardized rate per 100000 population (95% UI) |  | Relative change (%) | Estimated annual percentage changes (95% CI) |
| Global | 1836282 | 119.92 (120.09 to 119.75) |  | 2267874 | 96.47 (96.6 to 96.34) |  | 23.5 | -0.74 (-0.89 to -0.59) |
| Socio-demographic index |  |  |  |  |  |  |  |  |
| High | 142768 | 50.32 (50.58 to 50.06) |  | 98242 | 31.5 (31.69 to 31.3) |  | -31.19 | -1.45 (-1.73 to -1.17) |
| High-middle | 384363 | 112.97 (113.33 to 112.62) |  | 367755 | 90.22 (90.52 to 89.93) |  | -4.32 | -0.96 (-1.22 to -0.69) |
| Middle | 692767 | 139.87 (140.2 to 139.54) |  | 810238 | 105.58 (105.81 to 105.35) |  | 16.96 | -0.87 (-1.03 to -0.71) |
| Low-middle | 447104 | 151.82 (152.26 to 151.37) |  | 660379 | 113.96 (114.24 to 113.69) |  | 47.7 | -0.9 (-1.03 to -0.78) |
| Low | 167302 | 144.71 (145.41 to 144.01) |  | 329152 | 116.4 (116.8 to 116) |  | 96.74 | -0.8 (-0.88 to -0.72) |
| GBD regions |  |  |  |  |  |  |  |  |
| High-income Asia Pacific | 38684 | 74.51 (75.26 to 73.77) |  | 10896 | 23.9 (24.36 to 23.45) |  | -71.83 | -4.55 (-5 to -4.1) |
| Central Asia | 29825 | 149.7 (151.43 to 148) |  | 28265 | 90.65 (91.71 to 89.6) |  | -5.23 | -2.16 (-2.57 to -1.75) |
| East Asia | 447953 | 114.4 (114.74 to 114.06) |  | 511996 | 113.4 (113.71 to 113.09) |  | 14.3 | -0.03 (-0.27 to 0.21) |
| South Asia | 308561 | 107.25 (107.63 to 106.87) |  | 461359 | 79.44 (79.67 to 79.21) |  | 49.52 | -0.95 (-1.13 to -0.78) |
| Southeast Asia | 334778 | 257.74 (258.62 to 256.86) |  | 466282 | 212.78 (213.39 to 212.17) |  | 39.28 | -0.45 (-0.61 to -0.29) |
| Australasia | 1136 | 17.49 (18.54 to 16.49) |  | 835 | 9.26 (9.92 to 8.64) |  | -26.5 | -2.54 (-2.76 to -2.32) |
| Caribbean | 11781 | 120.57 (122.79 to 118.39) |  | 16677 | 117.94 (119.74 to 116.15) |  | 41.56 | 0.5 (0.21 to 0.79) |
| Central Europe | 44276 | 112.91 (113.97 to 111.86) |  | 14040 | 43.03 (43.75 to 42.31) |  | -68.29 | -3.63 (-3.83 to -3.43) |
| Eastern Europe | 66632 | 89.93 (90.61 to 89.24) |  | 68441 | 102.01 (102.78 to 101.23) |  | 2.71 | -0.23 (-0.83 to 0.37) |
| Western Europe | 52961 | 46.33 (46.72 to 45.93) |  | 14313 | 12.85 (13.06 to 12.64) |  | -72.97 | -4.41 (-4.61 to -4.22) |
| Andean Latin America | 7240 | 71.88 (73.57 to 70.22) |  | 10555 | 50.74 (51.72 to 49.77) |  | 45.79 | -0.96 (-1.33 to -0.58) |
| Central Latin America | 29950 | 68.48 (69.27 to 67.7) |  | 33181 | 43.02 (43.48 to 42.56) |  | 10.79 | -1.69 (-2.13 to -1.25) |
| Southern Latin America | 11807 | 84.87 (86.41 to 83.34) |  | 8666 | 41.9 (42.79 to 41.02) |  | -26.6 | -2.12 (-2.3 to -1.95) |
| Tropical Latin America | 79168 | 181.89 (183.17 to 180.62) |  | 43774 | 59.59 (60.15 to 59.03) |  | -44.71 | -3.84 (-4.08 to -3.6) |
| North Africa and Middle East | 149420 | 172.92 (173.8 to 172.04) |  | 186413 | 91.52 (91.94 to 91.11) |  | 24.76 | -2.14 (-2.18 to -2.09) |
| High-income North America | 25572 | 26.79 (27.12 to 26.47) |  | 23586 | 23.31 (23.61 to 23.02) |  | -7.77 | -0.26 (-0.52 to 0) |
| Oceania | 3218 | 187.23 (193.88 to 180.76) |  | 8328 | 207.74 (212.27 to 203.29) |  | 158.79 | 0.37 (0.19 to 0.56) |
| Central Sub-Saharan Africa | 20431 | 159.92 (162.15 to 157.72) |  | 35955 | 105.57 (106.68 to 104.48) |  | 75.98 | -1.54 (-1.63 to -1.45) |
| Eastern Sub-Saharan Africa | 72248 | 170.47 (171.73 to 169.22) |  | 145783 | 135.02 (135.72 to 134.32) |  | 101.78 | -0.89 (-0.96 to -0.82) |
| Southern Sub-Saharan Africa | 36143 | 263.14 (265.88 to 260.41) |  | 35975 | 134.51 (135.91 to 133.13) |  | -0.46 | -2.45 (-3.37 to -1.52) |
| Western Sub-Saharan Africa | 64497 | 145.25 (146.38 to 144.12) |  | 142554 | 120.34 (120.97 to 119.71) |  | 121.02 | -0.51 (-0.66 to -0.36) |

**Supplementary Table S8. Global disability-adjusted life years (DALYs) of subarachnoid hemorrhage attributable to metabolic risk factors among young adults in 1990 and 2021, and estimated annual percentage changes from 1990 to 2021**

| Characteristics | 1990 | |  | 2021 | |  | 1990−2021 | |
| --- | --- | --- | --- | --- | --- | --- | --- | --- |
|  | Number of DALYs | Age-standardized rate per 100000 population (95% UI) |  | Number of DALYs | Age-standardized rate per 100000 population (95% UI) |  | Relative change (%) | Estimated annual percentage changes (95% CI) |
| Global | 525957 | 34.25 (34.34 to 34.15) |  | 544954 | 23.18 (23.24 to 23.12) |  | 3.61 | -1.37 (-1.43 to -1.32) |
| Socio-demographic index |  |  |  |  |  |  |  |  |
| High | 96214 | 33.91 (34.12 to 33.69) |  | 47843 | 15.4 (15.54 to 15.26) |  | -50.27 | -2.7 (-2.87 to -2.53) |
| High-middle | 115381 | 33.83 (34.03 to 33.64) |  | 84355 | 20.88 (21.02 to 20.74) |  | -26.89 | -1.77 (-1.91 to -1.63) |
| Middle | 183944 | 36.99 (37.16 to 36.82) |  | 183098 | 23.93 (24.04 to 23.82) |  | -0.46 | -1.55 (-1.64 to -1.46) |
| Low-middle | 104975 | 35.46 (35.67 to 35.24) |  | 171074 | 29.42 (29.56 to 29.28) |  | 62.97 | -0.57 (-0.67 to -0.48) |
| Low | 24793 | 21.35 (21.62 to 21.08) |  | 57957 | 20.37 (20.54 to 20.2) |  | 133.76 | -0.17 (-0.27 to -0.07) |
| GBD regions |  |  |  |  |  |  |  |  |
| High-income Asia Pacific | 23437 | 45.12 (45.7 to 44.54) |  | 7945 | 17.44 (17.83 to 17.06) |  | -66.1 | -3.67 (-3.97 to -3.37) |
| Central Asia | 6011 | 30 (30.78 to 29.24) |  | 8153 | 26.11 (26.69 to 25.55) |  | 35.63 | -0.48 (-0.73 to -0.23) |
| East Asia | 120671 | 30.74 (30.91 to 30.56) |  | 70069 | 15.69 (15.81 to 15.58) |  | -41.93 | -2.79 (-3.15 to -2.42) |
| South Asia | 95084 | 32.88 (33.09 to 32.67) |  | 150247 | 25.79 (25.92 to 25.66) |  | 58.02 | -0.79 (-0.94 to -0.63) |
| Southeast Asia | 54844 | 41.74 (42.1 to 41.39) |  | 79006 | 35.99 (36.25 to 35.74) |  | 44.06 | -0.27 (-0.43 to -0.12) |
| Australasia | 1616 | 24.87 (26.11 to 23.67) |  | 1144 | 12.68 (13.44 to 11.96) |  | -29.21 | -2.71 (-3.01 to -2.42) |
| Caribbean | 4313 | 43.6 (44.93 to 42.3) |  | 7079 | 49.92 (51.1 to 48.76) |  | 64.13 | 0.99 (0.73 to 1.26) |
| Central Europe | 20376 | 52.18 (52.9 to 51.46) |  | 8904 | 27.5 (28.08 to 26.93) |  | -56.3 | -1.99 (-2.13 to -1.85) |
| Eastern Europe | 31859 | 43.03 (43.51 to 42.56) |  | 30833 | 46.72 (47.26 to 46.2) |  | -3.22 | -0.07 (-0.61 to 0.48) |
| Western Europe | 40205 | 35.11 (35.46 to 34.77) |  | 14088 | 12.64 (12.85 to 12.43) |  | -64.96 | -3.42 (-3.53 to -3.3) |
| Andean Latin America | 3200 | 31.48 (32.6 to 30.39) |  | 8053 | 38.51 (39.36 to 37.67) |  | 151.66 | 1.29 (0.99 to 1.6) |
| Central Latin America | 14480 | 32.8 (33.34 to 32.26) |  | 24671 | 31.85 (32.25 to 31.46) |  | 70.38 | 0.07 (-0.2 to 0.34) |
| Southern Latin America | 6727 | 48.12 (49.29 to 46.98) |  | 5958 | 28.78 (29.52 to 28.06) |  | -11.43 | -1.33 (-1.5 to -1.17) |
| Tropical Latin America | 35051 | 79.99 (80.84 to 79.15) |  | 29430 | 40.12 (40.58 to 39.67) |  | -16.04 | -2.5 (-2.62 to -2.39) |
| North Africa and Middle East | 24319 | 28.47 (28.84 to 28.11) |  | 30533 | 14.96 (15.13 to 14.79) |  | 25.55 | -2.23 (-2.29 to -2.16) |
| High-income North America | 23751 | 24.88 (25.2 to 24.57) |  | 18047 | 17.87 (18.14 to 17.61) |  | -24.02 | -1.02 (-1.32 to -0.72) |
| Oceania | 715 | 40.46 (43.58 to 37.51) |  | 1939 | 47.57 (49.75 to 45.47) |  | 171.19 | 0.57 (0.36 to 0.77) |
| Central Sub-Saharan Africa | 2242 | 17.37 (18.11 to 16.65) |  | 4984 | 14.51 (14.92 to 14.11) |  | 122.3 | -0.75 (-0.87 to -0.63) |
| Eastern Sub-Saharan Africa | 7635 | 17.84 (18.25 to 17.44) |  | 20333 | 18.63 (18.89 to 18.37) |  | 166.31 | 0.1 (0.04 to 0.17) |
| Southern Sub-Saharan Africa | 2433 | 17.7 (18.42 to 17) |  | 4239 | 15.83 (16.32 to 15.36) |  | 74.23 | -0.21 (-0.9 to 0.49) |
| Western Sub-Saharan Africa | 6988 | 15.57 (15.95 to 15.21) |  | 19299 | 16.1 (16.33 to 15.87) |  | 176.17 | 0.27 (0.1 to 0.43) |

**Supplementary Table S9. Disability-adjusted life years (DALYs) of ischemic stroke attributable to metabolic risk factors among young adults in 1990 and 2021, and estimated annual percentage changes from 1990 to 2021, by country and territories.**

| **Countries** | Number of cases in 1990 | Age-standardized rate per 100000 population (95% UI) in 1990 | Number of cases in 2021 | Age-standardized rate per 100000 population (95% UI) in 2021 | Estimated annual percentage changes (95% CI) from 1990 to 2021 |
| --- | --- | --- | --- | --- | --- |
| Afghanistan | 1267 | 148.59 (155.2 to 142.21) | 2115 | 150.49 (153.51 to 147.52) | 0.11 (0.01 to 0.22) |
| Albania | 218866 | 39.16 (43.26 to 35.37) | 247522 | 35.91 (40.48 to 31.75) | -0.19 (-0.3 to -0.09) |
| Algeria | 49098 | 113.89 (116.63 to 111.21) | 73509 | 81.35 (82.82 to 79.89) | -1.34 (-1.47 to -1.21) |
| American Samoa | 4547 | 81.12 (147.53 to 40.11) | 6753 | 75.9 (143.1 to 35.32) | -0.41 (-0.49 to -0.32) |
| Andorra | 4069 | 22.06 (54.46 to 6.91) | 2739 | 15.67 (44.95 to 3.82) | -1.21 (-1.27 to -1.15) |
| Angola | 11017 | 42.85 (45.5 to 40.33) | 11557 | 39.2 (40.63 to 37.8) | -0.24 (-0.32 to -0.16) |
| Antigua and Barbuda | 1028 | 36.79 (77.76 to 14.49) | 1759 | 18.19 (42.46 to 5.98) | -1.59 (-1.82 to -1.36) |
| Argentina | 10275 | 33.09 (34.3 to 31.91) | 22983 | 20.37 (21.13 to 19.62) | -1.41 (-1.58 to -1.23) |
| Armenia | 4596 | 75.26 (80.55 to 70.24) | 4069 | 47.85 (52.4 to 43.65) | -1.84 (-2.05 to -1.62) |
| Australia | 3466 | 24.44 (25.79 to 23.14) | 6898 | 17.55 (18.53 to 16.61) | -1.26 (-1.35 to -1.17) |
| Austria | 8585 | 39.45 (42.08 to 36.94) | 11314 | 18.47 (20.26 to 16.81) | -2.56 (-2.82 to -2.3) |
| Azerbaijan | 46 | 65.51 (69.03 to 62.13) | 115 | 42.24 (44.37 to 40.19) | -1.92 (-2.09 to -1.74) |
| Bahamas | 199 | 41.56 (58.45 to 28.7) | 225 | 32.09 (44.03 to 22.74) | -1.26 (-1.42 to -1.09) |
| Bahrain | 109 | 59 (70.14 to 49.31) | 190 | 52.78 (58.86 to 47.21) | -0.68 (-0.93 to -0.43) |
| Bangladesh | 25 | 48.33 (49.19 to 47.48) | 52 | 44.87 (45.45 to 44.29) | -0.17 (-0.53 to 0.2) |
| Barbados | 454 | 42.22 (58.71 to 29.45) | 1276 | 27.82 (42.01 to 17.53) | -1.54 (-1.74 to -1.34) |
| Belarus | 10 | 75 (78.02 to 72.07) | 19 | 56.98 (59.86 to 54.23) | -1.41 (-1.87 to -0.96) |
| Belgium | 10390 | 35.6 (37.78 to 33.53) | 18086 | 15.47 (16.95 to 14.08) | -2.52 (-2.72 to -2.31) |
| Belize | 27 | 24.46 (44.5 to 12.22) | 44 | 23.46 (33.35 to 15.95) | -0.17 (-0.51 to 0.17) |
| Benin | 29 | 49.14 (53.51 to 45.04) | 32 | 52.62 (55.19 to 50.14) | 0.19 (0.03 to 0.35) |
| Bermuda | 44 | 27.81 (61.34 to 10.47) | 121 | 16.51 (57.3 to 2.79) | -1.63 (-1.93 to -1.33) |
| Bhutan | 12 | 31.28 (41.66 to 23.02) | 16 | 26.32 (33.21 to 20.56) | -0.83 (-0.91 to -0.76) |
| Bolivia (Plurinational State of) | 44 | 46.09 (49.52 to 42.85) | 103 | 22.82 (24.43 to 21.3) | -2.59 (-2.91 to -2.27) |
| Bosnia and Herzegovina | 847 | 87.82 (92.72 to 83.12) | 481 | 73.25 (79.19 to 67.66) | -0.72 (-0.81 to -0.64) |
| Botswana | 1521 | 41.58 (49.53 to 34.66) | 1795 | 34.78 (38.97 to 30.95) | -0.63 (-0.77 to -0.49) |
| Brazil | 1456 | 56.18 (56.9 to 55.47) | 1595 | 25.44 (25.81 to 25.07) | -2.85 (-3.41 to -2.28) |
| Brunei Darussalam | 299 | 59.74 (77.75 to 45.11) | 595 | 31.89 (41.53 to 24.06) | -2.23 (-2.48 to -1.98) |
| Bulgaria | 1374 | 90.61 (94.5 to 86.86) | 788 | 81.07 (85.42 to 76.92) | -0.53 (-0.69 to -0.38) |
| Burkina Faso | 7569 | 35.45 (38.22 to 32.83) | 5064 | 34.33 (35.92 to 32.8) | -0.15 (-0.23 to -0.07) |
| Burundi | 1485 | 59.69 (64 to 55.62) | 2132 | 36.73 (38.79 to 34.75) | -1.95 (-2.14 to -1.76) |
| Cabo Verde | 399 | 95.31 (121.49 to 73.86) | 270 | 63.14 (75.15 to 52.64) | -1.06 (-1.34 to -0.77) |
| Cambodia | 1214 | 51.85 (54.82 to 49) | 2286 | 37.69 (39.33 to 36.1) | -1.26 (-1.36 to -1.16) |
| Cameroon | 1306 | 41.26 (43.97 to 38.69) | 640 | 61.7 (63.4 to 60.03) | 1.47 (0.89 to 2.04) |
| Canada | 5829 | 30.97 (32.11 to 29.87) | 10074 | 25.58 (26.58 to 24.6) | -0.82 (-1.04 to -0.59) |
| Central African Republic | 1974 | 44.42 (49.94 to 39.39) | 860 | 40.93 (44.47 to 37.61) | -0.39 (-0.46 to -0.33) |
| Chad | 2197 | 54.75 (58.93 to 50.8) | 1460 | 60.18 (62.78 to 57.66) | 0.32 (0.21 to 0.42) |
| Chile | 3586 | 33.71 (35.54 to 31.95) | 1025 | 22.82 (24.08 to 21.61) | -1.16 (-1.29 to -1.04) |
| China | 564 | 57.27 (57.52 to 57.03) | 371 | 57.35 (57.58 to 57.12) | -0.03 (-0.11 to 0.05) |
| Colombia | 803 | 38.45 (39.72 to 37.21) | 301 | 18.51 (19.2 to 17.84) | -2.26 (-2.7 to -1.82) |
| Comoros | 70 | 53.58 (70.22 to 40.15) | 55 | 40.04 (49.4 to 32.09) | -1.27 (-1.55 to -0.99) |
| Congo | 2539 | 53.99 (60.52 to 48.02) | 1217 | 47.93 (51.48 to 44.57) | -0.65 (-0.8 to -0.49) |
| Cook Islands | 8038 | 71.35 (193.52 to 18.04) | 4034 | 65.75 (200.68 to 12.7) | -0.1 (-0.25 to 0.06) |
| Costa Rica | 1141 | 27.36 (31.04 to 24.02) | 656 | 19.57 (21.89 to 17.44) | -1.31 (-1.53 to -1.09) |
| Côte d'Ivoire | 306 | 80.33 (83.65 to 77.11) | 120 | 81.91 (83.94 to 79.91) | 0.1 (-0.06 to 0.25) |
| Croatia | 268 | 52.22 (55.97 to 48.67) | 109 | 27.35 (30.66 to 24.33) | -2.18 (-2.28 to -2.07) |
| Cuba | 5111 | 37.15 (39.33 to 35.07) | 2205 | 22.66 (24.43 to 21) | -1.25 (-1.42 to -1.08) |
| Cyprus | 590 | 20.68 (27.31 to 15.34) | 215 | 13.22 (17.07 to 10.16) | -2 (-2.29 to -1.71) |
| Czechia | 13607 | 64.7 (67.64 to 61.86) | 12472 | 31.43 (33.64 to 29.35) | -2.27 (-2.41 to -2.13) |
| Democratic People's Republic of Korea | 850 | 77.42 (79.73 to 75.17) | 324 | 80.39 (82.33 to 78.48) | -0.18 (-0.3 to -0.05) |
| Democratic Republic of the Congo | 2476 | 39.83 (41.18 to 38.52) | 1645 | 30.01 (30.73 to 29.3) | -1.06 (-1.14 to -0.99) |
| Denmark | 793 | 32.87 (35.88 to 30.05) | 500 | 14.64 (16.75 to 12.74) | -2.94 (-3.12 to -2.75) |
| Djibouti | 50617 | 37.74 (51.89 to 26.77) | 37888 | 39.31 (45.81 to 33.54) | 0.08 (-0.02 to 0.17) |
| Dominica | 56 | 25.7 (62.57 to 7.99) | 56 | 27.57 (62.09 to 9.57) | 0.19 (0.02 to 0.37) |
| Dominican Republic | 464 | 43.23 (46.29 to 40.33) | 351 | 44.33 (46.61 to 42.14) | 0.48 (0.24 to 0.72) |
| Ecuador | 11684 | 55.31 (58.21 to 52.52) | 6230 | 23.63 (24.95 to 22.36) | -2.73 (-3.04 to -2.41) |
| Egypt | 1326 | 147.63 (149.62 to 145.66) | 1314 | 141.71 (143.04 to 140.4) | 0.41 (0.15 to 0.68) |
| El Salvador | 5 | 41.66 (45.35 to 38.2) | 4 | 25.52 (27.96 to 23.26) | -1.59 (-1.98 to -1.2) |
| Equatorial Guinea | 8818 | 46.36 (62.46 to 33.6) | 3592 | 40.52 (46.99 to 34.77) | -0.59 (-0.69 to -0.48) |
| Eritrea | 324 | 38.18 (42.83 to 33.93) | 293 | 35.73 (38.54 to 33.09) | -0.22 (-0.29 to -0.15) |
| Estonia | 931 | 64.5 (72.18 to 57.48) | 456 | 32.38 (38.94 to 26.79) | -3.09 (-3.42 to -2.76) |
| Eswatini | 841 | 30.97 (40.59 to 23.21) | 297 | 38.18 (45.19 to 32.03) | 0.97 (0.53 to 1.41) |
| Ethiopia | 1098 | 25.4 (26.34 to 24.48) | 460 | 21.53 (22.08 to 20.99) | -0.66 (-0.84 to -0.47) |
| Fiji | 50 | 88.15 (101.43 to 76.24) | 64 | 81.13 (92.49 to 70.86) | -0.35 (-0.44 to -0.26) |
| Finland | 4388 | 52.76 (56.47 to 49.25) | 2162 | 20.72 (23.24 to 18.43) | -2.82 (-3.19 to -2.45) |
| France | 501 | 24.65 (25.39 to 23.92) | 215 | 13.38 (13.96 to 12.83) | -2.04 (-2.16 to -1.92) |
| Gabon | 13463 | 39.64 (48.4 to 32.15) | 4976 | 38.55 (44.37 to 33.34) | -0.24 (-0.35 to -0.12) |
| Gambia | 358 | 78.74 (91.29 to 67.6) | 341 | 84.67 (92.22 to 77.6) | 0.08 (-0.12 to 0.27) |
| Georgia | 1215 | 81.26 (85.69 to 77.01) | 474 | 77.29 (82.93 to 71.98) | -0.71 (-1.08 to -0.33) |
| Germany | 5349 | 54.01 (54.93 to 53.1) | 2015 | 22.22 (22.85 to 21.6) | -2.76 (-3.09 to -2.44) |
| Ghana | 23 | 122.81 (126.46 to 119.23) | 16 | 113.67 (115.79 to 111.58) | -0.19 (-0.29 to -0.09) |
| Greece | 302 | 41.55 (43.95 to 39.24) | 171 | 19.45 (21.31 to 17.72) | -2.67 (-2.78 to -2.56) |
| Greenland | 56 | 54.74 (98.36 to 27.53) | 27 | 27.33 (67.08 to 8.43) | -2.53 (-2.66 to -2.41) |
| Grenada | 442 | 93.06 (143.77 to 57.23) | 241 | 34.93 (64.68 to 16.77) | -2.97 (-3.32 to -2.62) |
| Guam | 1982 | 58.73 (85.29 to 38.92) | 437 | 72.94 (104 to 49.35) | 0.82 (0.65 to 0.98) |
| Guatemala | 41 | 34.56 (37.35 to 31.94) | 19 | 24.29 (25.77 to 22.88) | -1.54 (-1.84 to -1.23) |
| Guinea | 3700 | 60.18 (64.41 to 56.16) | 1634 | 74.25 (77.26 to 71.33) | 0.9 (0.81 to 1) |
| Guinea-Bissau | 737 | 97.9 (111.75 to 85.43) | 625 | 101.64 (110.42 to 93.41) | 0.2 (0.16 to 0.24) |
| Guyana | 1570 | 77.12 (89.76 to 65.9) | 738 | 64.48 (76.29 to 54.13) | -0.43 (-0.78 to -0.08) |
| Haiti | 5329 | 92 (96.77 to 87.42) | 2937 | 68.92 (71.51 to 66.4) | -0.65 (-0.77 to -0.52) |
| Honduras | 600 | 66.71 (71.79 to 61.93) | 283 | 31.19 (33.28 to 29.2) | -3.04 (-3.35 to -2.73) |
| Hungary | 3000 | 111.39 (115.14 to 107.74) | 2829 | 42.4 (45.1 to 39.84) | -3.43 (-3.73 to -3.13) |
| Iceland | 1395 | 28.41 (42.65 to 18.04) | 1338 | 15.76 (25.85 to 8.96) | -1.8 (-2.01 to -1.59) |
| India | 2957 | 32.35 (32.58 to 32.12) | 2603 | 26.98 (27.13 to 26.83) | -0.71 (-0.87 to -0.56) |
| Indonesia | 428 | 93.27 (94.11 to 92.45) | 240 | 81.38 (81.97 to 80.79) | -0.43 (-0.55 to -0.31) |
| Iran (Islamic Republic of) | 35549 | 131.1 (133.07 to 129.16) | 34464 | 85.32 (86.34 to 84.3) | -1.34 (-1.39 to -1.29) |
| Iraq | 34 | 165.38 (169.28 to 161.56) | 38 | 111.73 (113.62 to 109.86) | -1.38 (-1.59 to -1.18) |
| Ireland | 35 | 29.96 (33.54 to 26.68) | 23 | 13.18 (15.35 to 11.26) | -2.91 (-3.04 to -2.79) |
| Israel | 7 | 25.39 (28.17 to 22.83) | 5 | 13.42 (14.93 to 12.03) | -2.31 (-2.58 to -2.03) |
| Italy | 844 | 32.7 (33.59 to 31.82) | 1535 | 14.93 (15.6 to 14.28) | -2.78 (-3.01 to -2.55) |
| Jamaica | 11 | 36.29 (41.39 to 31.7) | 31 | 30.54 (34.35 to 27.07) | -0.95 (-1.34 to -0.55) |
| Japan | 21 | 33.33 (33.94 to 32.73) | 10 | 22.09 (22.65 to 21.55) | -1.46 (-1.55 to -1.36) |
| Jordan | 5 | 135.14 (143.43 to 127.24) | 6 | 77.93 (80.77 to 75.16) | -2.08 (-2.24 to -1.92) |
| Kazakhstan | 1219 | 144.91 (148.23 to 141.65) | 684 | 83.46 (85.8 to 81.17) | -2.54 (-3.33 to -1.74) |
| Kenya | 171 | 32.29 (33.93 to 30.72) | 140 | 30.65 (31.58 to 29.74) | -0.14 (-0.26 to -0.03) |
| Kiribati | 16 | 121.91 (181.69 to 78.37) | 11 | 142.28 (187.12 to 106.02) | 0.42 (0.35 to 0.5) |
| Kuwait | 1517 | 66.05 (72.3 to 60.21) | 2823 | 61.83 (65.37 to 58.47) | -0.21 (-0.51 to 0.1) |
| Kyrgyzstan | 55 | 119.34 (125.56 to 113.36) | 70 | 85.13 (89.18 to 81.22) | -2.37 (-2.76 to -1.98) |
| Lao People's Democratic Republic | 231 | 103.53 (110.12 to 97.25) | 281 | 73.52 (77.05 to 70.11) | -1.25 (-1.3 to -1.19) |
| Latvia | 22 | 75.74 (82.12 to 69.75) | 17 | 42.21 (48.43 to 36.72) | -2.72 (-3.06 to -2.39) |
| Lebanon | 244 | 96.07 (103.28 to 89.26) | 219 | 61.15 (64.67 to 57.8) | -1.09 (-1.48 to -0.7) |
| Lesotho | 1499 | 22.62 (28.2 to 17.91) | 1299 | 35.68 (41.01 to 30.9) | 2.11 (1.83 to 2.39) |
| Liberia | 1771 | 67.03 (73.83 to 60.72) | 2799 | 71.19 (75.59 to 66.99) | 0.45 (0.34 to 0.57) |
| Libya | 546 | 105.19 (111.76 to 98.93) | 474 | 148.97 (153.87 to 144.2) | 1.46 (1.26 to 1.66) |
| Lithuania | 757 | 77.78 (83.2 to 72.63) | 842 | 46.02 (51.34 to 41.14) | -1.81 (-2.02 to -1.61) |
| Luxembourg | 3688 | 44.06 (57.28 to 33.33) | 2890 | 13.64 (20.06 to 8.99) | -4.32 (-4.62 to -4.03) |
| Madagascar | 651 | 70.84 (74.06 to 67.74) | 1118 | 63.56 (65.41 to 61.74) | -0.33 (-0.39 to -0.27) |
| Malawi | 247 | 44.04 (46.92 to 41.31) | 306 | 45.94 (47.89 to 44.05) | 0.01 (-0.17 to 0.19) |
| Malaysia | 729 | 65.9 (68.14 to 63.72) | 930 | 61.94 (63.42 to 60.49) | 0.02 (-0.19 to 0.23) |
| Maldives | 10349 | 100.01 (134.83 to 72.69) | 11097 | 43.58 (52.8 to 35.92) | -2.85 (-3.03 to -2.67) |
| Mali | 2058 | 55.45 (58.9 to 52.16) | 2121 | 52.09 (54.11 to 50.13) | -0.17 (-0.29 to -0.05) |
| Malta | 341 | 35.57 (48.46 to 25.55) | 505 | 15.28 (24.4 to 9.15) | -2.82 (-2.97 to -2.68) |
| Marshall Islands | 252 | 85.96 (161.85 to 40.3) | 278 | 105 (165.31 to 62.73) | 0.48 (0.31 to 0.65) |
| Mauritania | 7001 | 88.79 (97.56 to 80.64) | 11884 | 61.18 (66.06 to 56.59) | -1.27 (-1.37 to -1.17) |
| Mauritius | 24121 | 122.78 (134.61 to 111.76) | 18042 | 78.92 (88.62 to 70.04) | -0.79 (-1.04 to -0.54) |
| Mexico | 7288 | 45.7 (46.6 to 44.81) | 13787 | 28.01 (28.54 to 27.49) | -1.63 (-1.93 to -1.34) |
| Micronesia (Federated States of) | 133 | 111.11 (160.08 to 74.35) | 328 | 110.18 (156.31 to 75.08) | -0.08 (-0.11 to -0.04) |
| Monaco | 306 | 44.45 (122.28 to 10.98) | 423 | 28.41 (100.19 to 3.99) | -1.5 (-1.54 to -1.46) |
| Mongolia | 21676 | 53.55 (60.21 to 47.49) | 44726 | 54.94 (59.56 to 50.61) | 0.12 (-0.06 to 0.29) |
| Montenegro | 1149 | 35.86 (45.32 to 27.96) | 3011 | 32 (41.73 to 24.09) | -0.4 (-0.53 to -0.27) |
| Morocco | 17737 | 144.2 (147.1 to 141.34) | 27679 | 106.43 (108.33 to 104.55) | -1.09 (-1.37 to -0.82) |
| Mozambique | 474 | 43.67 (46.08 to 41.36) | 1322 | 61.4 (63.28 to 59.57) | 1.5 (1.34 to 1.65) |
| Myanmar | 749 | 96.64 (98.48 to 94.83) | 1254 | 69.91 (71.2 to 68.64) | -0.95 (-1.1 to -0.8) |
| Namibia | 475 | 36.1 (43.29 to 29.88) | 1047 | 32.41 (36.82 to 28.43) | -0.58 (-0.82 to -0.34) |
| Nauru | 569 | 205.98 (458.1 to 74.1) | 1865 | 221.86 (456.04 to 90.68) | 0.06 (-0.26 to 0.37) |
| Nepal | 1075 | 34.65 (36.37 to 32.99) | 3700 | 23.33 (24.35 to 22.35) | -1.5 (-1.6 to -1.4) |
| Netherlands | 9838 | 32.31 (33.95 to 30.73) | 12227 | 17.21 (18.5 to 15.99) | -2.26 (-2.38 to -2.14) |
| New Zealand | 127 | 30.3 (33.79 to 27.09) | 700 | 19.66 (22.05 to 17.47) | -1.6 (-1.77 to -1.43) |
| Nicaragua | 1637 | 38.07 (42.4 to 34.09) | 2669 | 23.67 (25.83 to 21.64) | -1.55 (-1.71 to -1.4) |
| Niger | 4956 | 44.86 (48.18 to 41.73) | 21066 | 41.76 (43.62 to 39.96) | -0.22 (-0.28 to -0.16) |
| Nigeria | 16503 | 45.16 (46.08 to 44.26) | 12686 | 51.28 (51.89 to 50.68) | 0.59 (0.5 to 0.68) |
| Niue | 780 | 92.01 (867.37 to 0.1) | 2775 | 94.96 (1119.13 to 0.02) | -0.35 (-0.48 to -0.22) |
| North Macedonia | 5324 | 92.24 (100.18 to 84.78) | 3380 | 53.38 (59.16 to 48.07) | -1.85 (-1.97 to -1.73) |
| Northern Mariana Islands | 2187 | 74.68 (125.23 to 41) | 10408 | 58.93 (120.05 to 24.72) | -1.01 (-1.19 to -0.83) |
| Norway | 12739 | 34.75 (38.15 to 31.58) | 22913 | 16.09 (18.27 to 14.12) | -2.68 (-2.82 to -2.54) |
| Oman | 2951 | 84.96 (92.29 to 78.1) | 9646 | 81.12 (84.97 to 77.44) | 0.4 (0.18 to 0.62) |
| Pakistan | 49 | 38.65 (39.44 to 37.88) | 71 | 49.3 (49.83 to 48.77) | 0.7 (0.6 to 0.79) |
| Palau | 9629 | 119.04 (262.49 to 43.37) | 34048 | 148.99 (305.64 to 61.92) | 0.99 (0.85 to 1.13) |
| Palestine | 75892 | 106.91 (117.32 to 97.26) | 122831 | 70.3 (74.76 to 66.04) | -1.39 (-1.62 to -1.16) |
| Panama | 304 | 36.87 (41.77 to 32.42) | 756 | 22.49 (25.3 to 19.92) | -1.66 (-1.9 to -1.41) |
| Papua New Guinea | 1661 | 42.01 (46.11 to 38.19) | 2127 | 41.6 (43.96 to 39.34) | -0.13 (-0.26 to 0) |
| Paraguay | 3559 | 28.81 (32.25 to 25.66) | 6911 | 18.31 (20.14 to 16.6) | -1.48 (-1.57 to -1.38) |
| Peru | 1088 | 30.35 (31.82 to 28.94) | 3027 | 24.17 (25.08 to 23.28) | -0.82 (-1.14 to -0.5) |
| Philippines | 291 | 60.69 (61.89 to 59.52) | 566 | 67.33 (68.21 to 66.46) | 0.7 (0.52 to 0.89) |
| Poland | 44 | 63.12 (64.52 to 61.74) | 181 | 34.5 (35.6 to 33.43) | -1.98 (-2.2 to -1.77) |
| Portugal | 813 | 69.7 (72.84 to 66.66) | 1295 | 17.5 (19.23 to 15.89) | -5.01 (-5.37 to -4.65) |
| Puerto Rico | 99 | 28.61 (32.05 to 25.45) | 197 | 17.01 (20.07 to 14.3) | -1.78 (-2.01 to -1.55) |
| Qatar | 54 | 55.68 (66.62 to 46.38) | 88 | 39.11 (42.28 to 36.19) | -1.46 (-1.65 to -1.27) |
| Republic of Korea | 39 | 56.81 (58.01 to 55.62) | 165 | 24.97 (25.8 to 24.15) | -3.42 (-3.71 to -3.13) |
| Republic of Moldova | 2000 | 53.68 (57.57 to 50.01) | 4703 | 40.83 (44.67 to 37.29) | -0.94 (-1.17 to -0.71) |
| Romania | 997 | 75.66 (77.78 to 73.59) | 2254 | 47.06 (49.08 to 45.1) | -1.98 (-2.17 to -1.8) |
| Russian Federation | 298 | 99.66 (100.53 to 98.79) | 681 | 82.13 (82.98 to 81.29) | -1.05 (-1.42 to -0.68) |
| Rwanda | 2911 | 54.91 (58.5 to 51.5) | 6231 | 26.1 (27.76 to 24.51) | -3.17 (-3.52 to -2.81) |
| Saint Kitts and Nevis | 1650 | 119.27 (202.07 to 65.12) | 4340 | 29.02 (66.06 to 10.09) | -4.64 (-5.22 to -4.05) |
| Saint Lucia | 20 | 63.3 (97.14 to 39.29) | 22 | 31.22 (50.17 to 18.16) | -2.26 (-2.55 to -1.97) |
| Saint Vincent and the Grenadines | 458 | 54.5 (90.91 to 30.52) | 287 | 35.21 (63.04 to 17.72) | -1.98 (-2.25 to -1.72) |
| Samoa | 694 | 69.84 (102.53 to 45.81) | 1554 | 81.44 (109.6 to 59.16) | 0.5 (0.44 to 0.56) |
| San Marino | 1950 | 27.75 (102.34 to 3.29) | 6582 | 17.56 (87.11 to 0.83) | -1.47 (-1.59 to -1.34) |
| Sao Tome and Principe | 1339 | 86.68 (134.68 to 53.02) | 4345 | 92.29 (119.6 to 69.94) | 0.14 (-0.17 to 0.46) |
| Saudi Arabia | 977 | 109.91 (113.04 to 106.84) | 1008 | 120.22 (121.86 to 118.59) | 0.69 (0.49 to 0.89) |
| Senegal | 1322 | 88.17 (92.84 to 83.69) | 3154 | 66.85 (69.41 to 64.36) | -0.88 (-1.02 to -0.73) |
| Serbia | 129 | 87.2 (90.67 to 83.85) | 299 | 47.22 (49.96 to 44.59) | -2.08 (-2.15 to -2.01) |
| Seychelles | 80 | 95.45 (150.29 to 57.49) | 201 | 66.24 (101.46 to 41.29) | -1.2 (-1.36 to -1.04) |
| Sierra Leone | 540 | 83.63 (89.38 to 78.18) | 1862 | 90.1 (94.03 to 86.3) | 0.52 (0.37 to 0.66) |
| Singapore | 121 | 38.93 (42.65 to 35.46) | 240 | 17.72 (19.76 to 15.89) | -2.75 (-2.92 to -2.59) |
| Slovakia | 9646 | 66.93 (70.94 to 63.09) | 10186 | 40.67 (43.96 to 37.6) | -1.5 (-1.6 to -1.41) |
| Slovenia | 644 | 41.5 (46.81 to 36.67) | 2011 | 20.73 (25.15 to 16.98) | -2.55 (-2.69 to -2.4) |
| Solomon Islands | 55 | 58.92 (79.72 to 42.55) | 137 | 65.04 (77.8 to 53.92) | 0.28 (0.2 to 0.36) |
| Somalia | 72 | 35.89 (38.66 to 33.26) | 127 | 31.33 (32.94 to 29.78) | -0.49 (-0.57 to -0.41) |
| South Africa | 541 | 92.46 (94.35 to 90.61) | 1729 | 50.81 (51.81 to 49.83) | -2.13 (-2.86 to -1.39) |
| South Sudan | 691 | 32.77 (36.03 to 29.75) | 1898 | 32.09 (34.61 to 29.72) | -0.11 (-0.28 to 0.06) |
| Spain | 719 | 33.71 (34.81 to 32.63) | 2200 | 15.04 (15.79 to 14.31) | -2.94 (-3.23 to -2.64) |
| Sri Lanka | 979 | 85.51 (88.02 to 83.05) | 5263 | 64.88 (66.91 to 62.9) | -1.07 (-1.25 to -0.89) |
| Sudan | 840 | 146.16 (149.67 to 142.71) | 2475 | 134.8 (136.88 to 132.75) | -0.1 (-0.25 to 0.06) |
| Suriname | 2423 | 53.8 (70.53 to 40.32) | 6404 | 42.35 (53.54 to 33) | -0.97 (-1.25 to -0.7) |
| Sweden | 225 | 30.96 (33.29 to 28.77) | 570 | 22.41 (24.25 to 20.68) | -1.01 (-1.17 to -0.84) |
| Switzerland | 185 | 26.88 (29.13 to 24.77) | 538 | 11.3 (12.71 to 10.02) | -3.19 (-3.47 to -2.9) |
| Syrian Arab Republic | 418 | 184.74 (189.87 to 179.73) | 1077 | 111.48 (115.58 to 107.51) | -1.72 (-1.97 to -1.47) |
| Taiwan (Province of China) | 4537 | 55.69 (57.43 to 53.99) | 11330 | 40.25 (41.8 to 38.75) | -0.65 (-0.86 to -0.44) |
| Tajikistan | 9662 | 111.25 (117.19 to 105.54) | 28239 | 67.25 (70.19 to 64.42) | -2.36 (-2.72 to -2) |
| Thailand | 1069 | 47.25 (48.26 to 46.25) | 2738 | 62.92 (64.1 to 61.76) | 0.4 (-0.05 to 0.86) |
| Timor-Leste | 21 | 49.93 (60.45 to 40.89) | 57 | 56.77 (65.65 to 48.86) | 0.55 (0.23 to 0.86) |
| Togo | 440 | 80.8 (87.24 to 74.74) | 668 | 70.86 (74.4 to 67.44) | -0.59 (-0.75 to -0.43) |
| Tokelau | 777 | 76.19 (1174.98 to 0) | 2086 | 89.77 (1267.66 to 0) | 0.16 (0.04 to 0.28) |
| Tonga | 1486 | 55.57 (99.51 to 28.21) | 2761 | 59.37 (97.74 to 33.49) | 0.17 (0.15 to 0.2) |
| Trinidad and Tobago | 6 | 66.45 (75.42 to 58.32) | 3 | 49.27 (56.39 to 42.91) | -1.25 (-1.5 to -0.99) |
| Tunisia | 674 | 73.25 (76.94 to 69.71) | 1627 | 70.98 (73.74 to 68.3) | -0.12 (-0.23 to -0.01) |
| Turkey | 11 | 102.88 (104.47 to 101.31) | 9 | 49.3 (50.16 to 48.44) | -2.58 (-2.68 to -2.48) |
| Turkmenistan | 890 | 119.51 (126.54 to 112.79) | 2162 | 142.61 (148.59 to 136.81) | 0.63 (0.23 to 1.04) |
| Tuvalu | 4 | 95.91 (307.12 to 16.42) | 2 | 104.07 (281.84 to 26.01) | 0.24 (0.18 to 0.3) |
| Uganda | 4 | 35.45 (37.46 to 33.53) | 3 | 31 (32.12 to 29.91) | -1.01 (-1.28 to -0.74) |
| Ukraine | 6 | 85.01 (86.46 to 83.59) | 7 | 90.47 (92.1 to 88.87) | -0.39 (-0.75 to -0.02) |
| United Arab Emirates | 11 | 86.78 (93.16 to 80.78) | 5 | 61.07 (64.31 to 57.99) | -1.32 (-1.49 to -1.14) |
| United Kingdom | 28 | 32.27 (33.15 to 31.4) | 31 | 15.88 (16.47 to 15.31) | -2.56 (-2.67 to -2.44) |
| United Republic of Tanzania | 6 | 33.51 (35.05 to 32.02) | 8 | 44.26 (45.35 to 43.19) | 0.76 (0.61 to 0.91) |
| United States of America | 298 | 41.38 (41.81 to 40.95) | 140 | 38.03 (38.43 to 37.62) | -0.37 (-0.47 to -0.27) |
| United States Virgin Islands | 14 | 34.52 (63.04 to 16.81) | 6 | 28.18 (65.79 to 9.61) | -0.59 (-0.77 to -0.42) |
| Uruguay | 1 | 50.13 (55.12 to 45.49) | 0 | 25.37 (28.8 to 22.26) | -2.23 (-2.42 to -2.04) |
| Uzbekistan | 2 | 101.24 (103.91 to 98.63) | 1 | 88.34 (90.08 to 86.62) | -0.55 (-1.03 to -0.08) |
| Vanuatu | 14 | 114.63 (154.43 to 83.13) | 8 | 121.88 (148.02 to 99.41) | 0.07 (0 to 0.13) |
| Venezuela (Bolivarian Republic of) | 0 | 37.05 (38.7 to 35.46) | 0 | 27.61 (28.82 to 26.44) | -1.27 (-1.66 to -0.89) |
| Viet Nam | 10 | 55.24 (56.33 to 54.17) | 5 | 53.33 (54.12 to 52.56) | 0.17 (-0.04 to 0.38) |
| Yemen | 3 | 100.58 (104.29 to 96.96) | 4 | 101.77 (103.83 to 99.75) | 0.01 (-0.22 to 0.23) |
| Zambia | 445 | 32.23 (35.14 to 29.52) | 677 | 35.95 (37.64 to 34.32) | 0.43 (0.32 to 0.54) |
| Zimbabwe | 6931 | 27.64 (29.89 to 25.52) | 16686 | 47.8 (49.95 to 45.73) | 2.3 (1.76 to 2.85) |

**Supplementary Table S10. Disability-adjusted life years (DALYs) of intracerebral hemorrhage attributable to metabolic risk factors among young adults in 1990 and 2021, and estimated annual percentage changes from 1990 to 2021, by country and territories.**

| **Countries** | Number of cases in 1990 | Age-standardized rate per 100000 population (95% UI) in 1990 | Number of cases in 2021 | Age-standardized rate per 100000 population (95% UI) in 2021 | Estimated annual percentage changes (95% CI) from 1990 to 2021 |
| --- | --- | --- | --- | --- | --- |
| Afghanistan | 4400 | 331.96 (341.75 to 322.42) | 5528 | 230.5 (234.21 to 226.83) | -1.29 (-1.48 to -1.11) |
| Albania | 428533 | 129.51 (136.83 to 122.51) | 491717 | 85.23 (92.16 to 78.7) | -1.4 (-1.77 to -1.02) |
| Algeria | 182194 | 156.73 (159.92 to 153.58) | 232893 | 69.97 (71.35 to 68.62) | -3.08 (-3.33 to -2.82) |
| American Samoa | 11899 | 355.07 (472.91 to 261.17) | 17325 | 339.26 (459.61 to 244.18) | -0.32 (-0.48 to -0.16) |
| Andorra | 7521 | 19.52 (50.96 to 5.53) | 2954 | 10.05 (36.65 to 1.49) | -2.15 (-2.27 to -2.02) |
| Angola | 165 | 174.88 (180.18 to 169.71) | 276 | 112.32 (114.74 to 109.93) | -1.45 (-1.62 to -1.29) |
| Antigua and Barbuda | 41482 | 98.88 (157.32 to 58.42) | 45165 | 28.09 (55.92 to 12.02) | -2.82 (-3.33 to -2.3) |
| Argentina | 32186 | 92.59 (94.6 to 90.62) | 67636 | 47.89 (49.06 to 46.75) | -2.07 (-2.23 to -1.9) |
| Armenia | 3917 | 71.87 (77.06 to 66.95) | 6046 | 22.26 (25.43 to 19.44) | -4.62 (-5.29 to -3.95) |
| Australia | 7665 | 17.27 (18.41 to 16.18) | 5901 | 9.27 (9.99 to 8.6) | -2.54 (-2.78 to -2.29) |
| Austria | 9810 | 38.58 (41.19 to 36.09) | 17334 | 10.82 (12.2 to 9.56) | -4.51 (-4.9 to -4.12) |
| Azerbaijan | 450 | 153.19 (158.56 to 147.97) | 715 | 61.34 (63.9 to 58.87) | -3.79 (-4.12 to -3.45) |
| Bahamas | 17395 | 118.07 (144.86 to 95.27) | 33088 | 79.47 (97.2 to 64.27) | -2.03 (-2.3 to -1.75) |
| Bahrain | 33796 | 91.34 (104.89 to 79.23) | 50323 | 67.54 (74.39 to 61.22) | -1.77 (-2.26 to -1.27) |
| Bangladesh | 136 | 228.79 (230.65 to 226.95) | 147 | 179.58 (180.75 to 178.41) | -0.66 (-1.03 to -0.29) |
| Barbados | 712 | 69.17 (89.61 to 52.44) | 717 | 42.15 (58.91 to 29.23) | -1.82 (-2.15 to -1.48) |
| Belarus | 112 | 109.48 (113.13 to 105.93) | 234 | 88.62 (92.1 to 85.27) | -1.56 (-2.39 to -0.72) |
| Belgium | 46 | 48.93 (51.47 to 46.49) | 93 | 13.01 (14.38 to 11.75) | -3.87 (-4.19 to -3.56) |
| Belize | 1314 | 68.97 (98.65 to 46.77) | 5366 | 54.29 (68.49 to 42.42) | -1.13 (-1.64 to -0.61) |
| Benin | 112 | 120.52 (127.34 to 113.99) | 203 | 119.08 (122.96 to 115.31) | -0.05 (-0.33 to 0.23) |
| Bermuda | 198 | 24.83 (57.32 to 8.69) | 487 | 10.21 (47.6 to 0.85) | -3.12 (-3.37 to -2.87) |
| Bhutan | 140 | 78.1 (93.78 to 64.53) | 445 | 43.98 (52.67 to 36.41) | -2.28 (-2.44 to -2.12) |
| Bolivia (Plurinational State of) | 28 | 102.64 (107.7 to 97.75) | 40 | 47.31 (49.6 to 45.11) | -2.88 (-3.3 to -2.45) |
| Bosnia and Herzegovina | 3921 | 61.92 (66.06 to 57.97) | 957 | 28.34 (32.12 to 24.92) | -2.86 (-3.08 to -2.63) |
| Botswana | 7531 | 164.86 (180.11 to 150.63) | 5609 | 82.04 (88.33 to 76.11) | -2.53 (-2.83 to -2.22) |
| Brazil | 804 | 183.67 (184.97 to 182.38) | 225 | 59.82 (60.39 to 59.25) | -3.87 (-4.11 to -3.63) |
| Brunei Darussalam | 1981 | 134.47 (160.42 to 111.86) | 1415 | 73.75 (87.71 to 61.58) | -2.48 (-3.02 to -1.93) |
| Bulgaria | 3368 | 217.84 (223.78 to 212.01) | 2320 | 110.87 (115.9 to 106.03) | -3.14 (-3.44 to -2.84) |
| Burkina Faso | 1079 | 110.42 (115.28 to 105.72) | 1580 | 90.53 (93.1 to 88.01) | -0.66 (-0.84 to -0.49) |
| Burundi | 7844 | 302.16 (311.7 to 292.84) | 10259 | 156.23 (160.45 to 152.11) | -2.45 (-2.78 to -2.11) |
| Cabo Verde | 2044 | 254.48 (295.18 to 218.43) | 2654 | 94.93 (109.43 to 81.93) | -2.9 (-3.31 to -2.5) |
| Cambodia | 1303 | 186.53 (192.18 to 181.01) | 633 | 99.64 (102.3 to 97.03) | -2.39 (-2.55 to -2.23) |
| Cameroon | 1329 | 132.65 (137.48 to 127.96) | 209 | 194.65 (197.67 to 191.67) | 1.29 (0.49 to 2.09) |
| Canada | 1253 | 16.76 (17.6 to 15.94) | 3245 | 12.09 (12.79 to 11.43) | -1.39 (-1.91 to -0.87) |
| Central African Republic | 918 | 218.56 (230.52 to 207.09) | 250 | 189.57 (197.06 to 182.31) | -0.65 (-0.77 to -0.53) |
| Chad | 1796 | 142.14 (148.83 to 135.69) | 445 | 144.77 (148.8 to 140.82) | 0.03 (-0.16 to 0.22) |
| Chile | 5328 | 65.78 (68.34 to 63.29) | 2010 | 28.46 (29.87 to 27.11) | -2.5 (-2.71 to -2.29) |
| China | 12714 | 113.19 (113.53 to 112.85) | 4518 | 112.71 (113.03 to 112.4) | 0 (-0.25 to 0.25) |
| Colombia | 5000 | 50.27 (51.74 to 48.83) | 744 | 19.63 (20.34 to 18.94) | -2.73 (-3.29 to -2.18) |
| Comoros | 8093 | 224.22 (256.07 to 195.52) | 2803 | 125.47 (141.32 to 111) | -2.48 (-3.06 to -1.9) |
| Congo | 888 | 233.77 (247.04 to 221.06) | 378 | 139.16 (145.13 to 133.37) | -2.17 (-2.46 to -1.89) |
| Cook Islands | 443 | 221.97 (395.87 to 112.58) | 198 | 172.86 (351.22 to 71.85) | -0.43 (-0.78 to -0.08) |
| Costa Rica | 3839 | 35.5 (39.7 to 31.65) | 1081 | 21.49 (23.92 to 19.26) | -2.17 (-2.62 to -1.72) |
| Côte d'Ivoire | 3589 | 193.62 (198.78 to 188.57) | 2668 | 169.28 (172.21 to 166.4) | -0.35 (-0.55 to -0.16) |
| Croatia | 276 | 85.78 (90.53 to 81.23) | 66 | 18.7 (21.44 to 16.24) | -5.27 (-5.5 to -5.04) |
| Cuba | 1590 | 68.25 (71.21 to 65.39) | 517 | 32.04 (34.13 to 30.04) | -2.35 (-2.73 to -1.98) |
| Cyprus | 327 | 32.35 (40.43 to 25.54) | 51 | 12.8 (16.57 to 9.8) | -3.84 (-4.39 to -3.3) |
| Czechia | 665 | 58.04 (60.82 to 55.37) | 206 | 16 (17.59 to 14.54) | -3.93 (-4.3 to -3.56) |
| Democratic People's Republic of Korea | 46837 | 206.68 (210.46 to 202.96) | 49134 | 206.08 (209.17 to 203.02) | -0.5 (-0.7 to -0.29) |
| Democratic Republic of the Congo | 742 | 146.46 (149.04 to 143.92) | 210 | 96.47 (97.77 to 95.19) | -1.58 (-1.68 to -1.48) |
| Denmark | 12391 | 39.67 (42.97 to 36.57) | 15267 | 11.95 (13.87 to 10.23) | -4.47 (-4.81 to -4.13) |
| Djibouti | 17690 | 160.51 (187.46 to 136.67) | 7375 | 113.43 (124.12 to 103.45) | -1.3 (-1.49 to -1.11) |
| Dominica | 2131 | 55.87 (103.41 to 26.84) | 891 | 55.26 (98.91 to 27.6) | -0.01 (-0.29 to 0.27) |
| Dominican Republic | 20395 | 111.05 (115.94 to 106.33) | 3095 | 100.7 (104.11 to 97.37) | 0.5 (0.16 to 0.85) |
| Ecuador | 125 | 78.97 (82.44 to 75.61) | 129 | 35.51 (37.12 to 33.95) | -2.53 (-2.85 to -2.21) |
| Egypt | 473 | 180.1 (182.29 to 177.94) | 296 | 105.13 (106.27 to 104) | -1.28 (-1.64 to -0.91) |
| El Salvador | 937 | 106.96 (112.82 to 101.34) | 699 | 50.48 (53.87 to 47.26) | -2.39 (-3.01 to -1.75) |
| Equatorial Guinea | 4 | 214.37 (246.43 to 185.57) | 2 | 91.08 (100.6 to 82.28) | -3.33 (-3.7 to -2.96) |
| Eritrea | 604 | 237.51 (248.76 to 226.66) | 173 | 165.17 (171.09 to 159.4) | -1.22 (-1.35 to -1.09) |
| Estonia | 198 | 57.95 (65.24 to 51.31) | 136 | 17.39 (22.35 to 13.42) | -5.44 (-5.98 to -4.89) |
| Eswatini | 1508 | 121.99 (140 to 105.83) | 389 | 156.22 (169.87 to 143.42) | 1.32 (0.43 to 2.22) |
| Ethiopia | 1001 | 96.47 (98.32 to 94.65) | 219 | 77.14 (78.17 to 76.11) | -0.86 (-1.14 to -0.58) |
| Fiji | 902 | 313.53 (337.64 to 290.78) | 268 | 258.7 (278.39 to 240.1) | -0.78 (-0.94 to -0.61) |
| Finland | 77 | 62.53 (66.55 to 58.71) | 62 | 15.19 (17.36 to 13.24) | -4.25 (-4.62 to -3.87) |
| France | 7652 | 42.93 (43.91 to 41.98) | 1893 | 11.75 (12.29 to 11.23) | -4.32 (-4.79 to -3.85) |
| Gabon | 17 | 147.09 (163.28 to 132.15) | 10 | 92.98 (101.86 to 84.71) | -1.73 (-1.89 to -1.57) |
| Gambia | 314 | 182.91 (201.63 to 165.59) | 120 | 192.84 (204.12 to 182.06) | -0.1 (-0.37 to 0.17) |
| Georgia | 13643 | 233.14 (240.58 to 225.89) | 2693 | 92.96 (99.09 to 87.16) | -4.31 (-5.67 to -2.93) |
| Germany | 2218 | 55.06 (55.99 to 54.14) | 661 | 11.96 (12.42 to 11.51) | -5.09 (-5.61 to -4.56) |
| Ghana | 577 | 331.69 (337.68 to 325.78) | 260 | 190.12 (192.87 to 187.4) | -1.76 (-2 to -1.51) |
| Greece | 7845 | 75.83 (79.06 to 72.71) | 2092 | 26.84 (29 to 24.81) | -3.72 (-4.13 to -3.3) |
| Greenland | 88 | 116.94 (174.99 to 74.81) | 20 | 39.2 (83.51 to 15.33) | -3.73 (-3.96 to -3.5) |
| Grenada | 1316 | 163.52 (227.53 to 114.23) | 430 | 64.17 (101.61 to 38.12) | -3.06 (-3.38 to -2.74) |
| Guam | 61 | 95.95 (128.41 to 70.13) | 21 | 162.58 (206.36 to 126.14) | 1.86 (1.54 to 2.18) |
| Guatemala | 268 | 95.76 (100.33 to 91.34) | 102 | 95.22 (98.12 to 92.38) | -0.62 (-1.15 to -0.08) |
| Guinea | 561 | 172.73 (179.82 to 165.86) | 128 | 187.38 (192.14 to 182.71) | 0.65 (0.49 to 0.82) |
| Guinea-Bissau | 3202 | 357.89 (383.62 to 333.51) | 586 | 292.19 (306.87 to 278.06) | -0.59 (-0.66 to -0.51) |
| Guyana | 5921 | 224.22 (245.25 to 204.61) | 1624 | 154.25 (172.03 to 137.91) | -1.18 (-1.42 to -0.94) |
| Haiti | 4610 | 304.64 (313.27 to 296.19) | 2348 | 239.51 (244.31 to 234.78) | -0.4 (-0.53 to -0.27) |
| Honduras | 520 | 143.93 (151.34 to 136.81) | 195 | 61.31 (64.23 to 58.49) | -3.48 (-3.85 to -3.12) |
| Hungary | 782 | 153.63 (157.99 to 149.37) | 356 | 30.57 (32.87 to 28.41) | -6.3 (-6.84 to -5.76) |
| Iceland | 8360 | 21.2 (33.89 to 12.4) | 6650 | 10.19 (18.82 to 4.93) | -2.11 (-2.49 to -1.73) |
| India | 2664 | 95.18 (95.58 to 94.79) | 1660 | 62.34 (62.57 to 62.11) | -1.36 (-1.68 to -1.03) |
| Indonesia | 1599 | 354.34 (355.98 to 352.71) | 1237 | 258.04 (259.09 to 257) | -0.88 (-1.07 to -0.7) |
| Iran (Islamic Republic of) | 18 | 51.35 (52.59 to 50.13) | 8 | 37.68 (38.37 to 37) | -0.73 (-0.94 to -0.51) |
| Iraq | 23948 | 281.04 (286.1 to 276.05) | 22341 | 149.97 (152.15 to 147.81) | -2.12 (-2.41 to -1.84) |
| Ireland | 95 | 31.14 (34.79 to 27.79) | 95 | 9.13 (10.95 to 7.55) | -4.19 (-4.48 to -3.9) |
| Israel | 10 | 40.93 (44.41 to 37.65) | 11 | 10.22 (11.54 to 9.01) | -4.75 (-5 to -4.51) |
| Italy | 57 | 48.24 (49.33 to 47.18) | 34 | 15.26 (15.93 to 14.61) | -3.9 (-4.16 to -3.65) |
| Jamaica | 2123 | 56.51 (62.86 to 50.67) | 3467 | 58.32 (63.52 to 53.45) | -0.53 (-1.17 to 0.12) |
| Japan | 32 | 49.76 (50.51 to 49.03) | 72 | 25.59 (26.18 to 25) | -2.36 (-2.61 to -2.11) |
| Jordan | 349 | 131.76 (139.91 to 123.99) | 530 | 51.39 (53.7 to 49.15) | -3.71 (-4.01 to -3.41) |
| Kazakhstan | 488 | 144.97 (148.29 to 141.7) | 331 | 92.16 (94.61 to 89.76) | -2.24 (-3.16 to -1.32) |
| Kenya | 36 | 96.12 (98.94 to 93.37) | 18 | 87.7 (89.28 to 86.15) | -0.11 (-0.45 to 0.23) |
| Kiribati | 2181 | 542.09 (654.52 to 445.18) | 964 | 639.46 (727.28 to 559.97) | 0.35 (0.19 to 0.52) |
| Kuwait | 46 | 49.16 (54.58 to 44.17) | 34 | 34.7 (37.42 to 32.17) | -1.57 (-2.67 to -0.46) |
| Kyrgyzstan | 4953 | 158.78 (166 to 151.8) | 9766 | 67.85 (71.5 to 64.35) | -4.56 (-5.26 to -3.86) |
| Lao People's Democratic Republic | 38 | 403.66 (416.59 to 391.04) | 26 | 255.74 (262.29 to 249.32) | -1.62 (-1.71 to -1.53) |
| Latvia | 1672 | 85.5 (92.26 to 79.12) | 1745 | 39.83 (45.82 to 34.55) | -3.87 (-4.43 to -3.31) |
| Lebanon | 152 | 186.29 (196.2 to 176.78) | 178 | 67.87 (71.57 to 64.33) | -2.67 (-3.34 to -2) |
| Lesotho | 278 | 78.74 (88.7 to 69.65) | 356 | 187.05 (198.87 to 175.78) | 4.1 (3.36 to 4.84) |
| Liberia | 2108 | 191.35 (202.65 to 180.53) | 1951 | 185.13 (192.15 to 178.31) | 0.07 (-0.11 to 0.25) |
| Libya | 3460 | 142.03 (149.6 to 134.76) | 6859 | 137.11 (141.82 to 132.51) | 0.04 (-0.25 to 0.33) |
| Lithuania | 314 | 68.7 (73.84 to 63.84) | 336 | 29.78 (34.13 to 25.88) | -2.61 (-3.15 to -2.06) |
| Luxembourg | 1384 | 68.95 (85.03 to 55.33) | 929 | 10.14 (15.84 to 6.23) | -6.96 (-7.26 to -6.66) |
| Madagascar | 1787 | 414.11 (421.8 to 406.54) | 4321 | 336.3 (340.55 to 332.09) | -0.59 (-0.67 to -0.51) |
| Malawi | 4668 | 184.35 (190.2 to 178.65) | 3053 | 161.37 (165.01 to 157.79) | -0.77 (-1.15 to -0.38) |
| Malaysia | 784 | 188.93 (192.72 to 185.19) | 971 | 156.49 (158.84 to 154.17) | -0.26 (-0.64 to 0.12) |
| Maldives | 398 | 372.3 (435.44 to 316.56) | 485 | 103.15 (116.52 to 91.27) | -4.25 (-4.57 to -3.94) |
| Mali | 1546 | 178.99 (185.14 to 172.99) | 1798 | 127.38 (130.54 to 124.28) | -1.05 (-1.28 to -0.83) |
| Malta | 6414 | 52.26 (67.4 to 39.96) | 5340 | 17.24 (26.7 to 10.72) | -3.35 (-3.58 to -3.11) |
| Marshall Islands | 12655 | 404.47 (542.22 to 295.1) | 15949 | 528.12 (647.3 to 426.25) | 0.82 (0.64 to 1.01) |
| Mauritania | 78021 | 211.7 (225.05 to 198.97) | 42566 | 101.96 (108.23 to 95.99) | -2.41 (-2.55 to -2.27) |
| Mauritius | 9682 | 212.79 (228.27 to 198.12) | 10196 | 188.87 (203.59 to 174.96) | 0.51 (0.14 to 0.89) |
| Mexico | 6915 | 56.68 (57.69 to 55.69) | 12028 | 40.33 (40.96 to 39.71) | -1.27 (-1.72 to -0.81) |
| Micronesia (Federated States of) | 1147 | 515.88 (611.11 to 432.41) | 1208 | 506.17 (595.71 to 427.25) | -0.04 (-0.11 to 0.03) |
| Monaco | 12361 | 55.93 (138.63 to 16.86) | 18596 | 32.7 (106.63 to 5.57) | -1.77 (-1.85 to -1.68) |
| Mongolia | 26662 | 208.34 (221.5 to 195.81) | 33252 | 145.61 (152.99 to 138.51) | -1.42 (-2.07 to -0.77) |
| Montenegro | 1457 | 227.2 (249.4 to 206.53) | 3389 | 114.13 (131.28 to 98.76) | -2.49 (-2.93 to -2.05) |
| Morocco | 207 | 221.53 (225.11 to 217.99) | 419 | 90.84 (92.6 to 89.11) | -3.24 (-3.49 to -2.99) |
| Mozambique | 1129 | 187.38 (192.34 to 182.53) | 1996 | 281.11 (285.13 to 277.14) | 1.96 (1.7 to 2.22) |
| Myanmar | 355 | 372.58 (376.21 to 368.98) | 731 | 274.88 (277.42 to 272.35) | -0.71 (-1.03 to -0.39) |
| Namibia | 1459 | 120.33 (133.03 to 108.59) | 1392 | 100.67 (108.26 to 93.49) | -1.06 (-1.62 to -0.5) |
| Nauru | 576 | 885.99 (1309.8 to 574.39) | 1037 | 1016.29 (1428.42 to 700.64) | 0.24 (-0.29 to 0.76) |
| Nepal | 15168 | 118.39 (121.54 to 115.3) | 10439 | 51.85 (53.37 to 50.37) | -2.89 (-3.03 to -2.75) |
| Netherlands | 10100 | 27.19 (28.7 to 25.74) | 4803 | 10.05 (11.04 to 9.12) | -3.49 (-3.74 to -3.24) |
| New Zealand | 603 | 18.61 (21.39 to 16.11) | 1378 | 9.18 (10.87 to 7.7) | -2.57 (-2.77 to -2.37) |
| Nicaragua | 220 | 89.25 (95.8 to 83.05) | 594 | 45.8 (48.78 to 42.96) | -2.12 (-2.32 to -1.91) |
| Niger | 2072 | 137.88 (143.65 to 132.28) | 2276 | 116.51 (119.63 to 113.46) | -0.47 (-0.59 to -0.36) |
| Nigeria | 24806 | 90.09 (91.39 to 88.79) | 12056 | 75.59 (76.33 to 74.85) | -0.51 (-0.7 to -0.31) |
| Niue | 7438 | 343.4 (1293.1 to 38.02) | 21300 | 340.59 (1543.59 to 21.85) | -0.89 (-1.14 to -0.63) |
| North Macedonia | 788 | 145.84 (155.77 to 136.41) | 1916 | 53.9 (59.68 to 48.59) | -3.25 (-3.5 to -3) |
| Northern Mariana Islands | 4867 | 255.23 (338.23 to 188.47) | 16099 | 188.88 (281.89 to 121.33) | -1.26 (-1.6 to -0.92) |
| Norway | 220835 | 21.12 (23.8 to 18.66) | 281934 | 6.77 (8.23 to 5.52) | -4.03 (-4.25 to -3.82) |
| Oman | 5495 | 89.61 (97.11 to 82.57) | 11085 | 60.22 (63.56 to 57.04) | -0.55 (-0.84 to -0.25) |
| Pakistan | 5613 | 90.04 (91.25 to 88.85) | 4646 | 121.71 (122.54 to 120.88) | 0.85 (0.68 to 1.01) |
| Palau | 60019 | 407.96 (628.58 to 251.05) | 91389 | 582.85 (843.73 to 388.23) | 1.51 (1.32 to 1.71) |
| Palestine | 118 | 128.98 (140.33 to 118.39) | 119 | 67.89 (72.23 to 63.75) | -2.1 (-2.4 to -1.8) |
| Panama | 1392 | 59.67 (65.88 to 53.92) | 2580 | 39.62 (43.32 to 36.17) | -1.59 (-1.92 to -1.27) |
| Papua New Guinea | 21976 | 125.79 (132.85 to 119.02) | 83273 | 177.21 (182.04 to 172.48) | 1.19 (0.83 to 1.56) |
| Paraguay | 1288 | 109.44 (116 to 103.17) | 2183 | 52.66 (55.72 to 49.73) | -2.35 (-2.5 to -2.21) |
| Peru | 4368 | 59.74 (61.79 to 57.75) | 8527 | 59.08 (60.5 to 57.69) | 0.48 (-0.03 to 1) |
| Philippines | 12826 | 195 (197.15 to 192.86) | 21797 | 201.31 (202.84 to 199.79) | 0.76 (0.45 to 1.07) |
| Poland | 4059 | 98.48 (100.22 to 96.77) | 5457 | 37.65 (38.78 to 36.55) | -3.22 (-3.39 to -3.05) |
| Portugal | 200 | 113 (116.99 to 109.12) | 400 | 23.16 (25.13 to 21.31) | -6.36 (-6.84 to -5.87) |
| Puerto Rico | 358 | 44.49 (48.75 to 40.51) | 467 | 23.95 (27.55 to 20.73) | -2.6 (-2.97 to -2.23) |
| Qatar | 224 | 96.79 (110.85 to 84.35) | 274 | 33.84 (36.85 to 31.08) | -4.04 (-4.48 to -3.6) |
| Republic of Korea | 164 | 134.53 (136.4 to 132.68) | 474 | 21.3 (22.06 to 20.55) | -7.33 (-7.98 to -6.68) |
| Republic of Moldova | 10741 | 143.78 (150.04 to 137.74) | 21883 | 70.87 (75.78 to 66.25) | -2.82 (-3.2 to -2.44) |
| Romania | 4761 | 118.37 (121 to 115.8) | 12228 | 58.82 (61.06 to 56.65) | -3.22 (-3.69 to -2.74) |
| Russian Federation | 11608 | 91.96 (92.8 to 91.13) | 24705 | 104.32 (105.26 to 103.39) | -0.18 (-0.84 to 0.48) |
| Rwanda | 1823 | 380.32 (389.61 to 371.21) | 3119 | 106.51 (109.84 to 103.26) | -5.46 (-6.06 to -4.86) |
| Saint Kitts and Nevis | 4053 | 310.88 (432.3 to 217.35) | 7803 | 52.46 (97.19 to 25.2) | -6.3 (-7.06 to -5.55) |
| Saint Lucia | 784 | 138.12 (185.33 to 100.67) | 683 | 63.18 (88.29 to 43.84) | -2.79 (-3.09 to -2.49) |
| Saint Vincent and the Grenadines | 6687 | 138.46 (191.47 to 97.59) | 4073 | 82.4 (120.81 to 53.97) | -2.3 (-2.59 to -2.01) |
| Samoa | 50 | 286.11 (345.91 to 234.67) | 42 | 371.24 (426.51 to 321.66) | 0.9 (0.76 to 1.04) |
| San Marino | 5670 | 32.3 (109.64 to 4.75) | 19497 | 13.23 (79.79 to 0.28) | -2.64 (-2.88 to -2.39) |
| Sao Tome and Principe | 2119 | 224.63 (295.95 to 167.33) | 8167 | 176.8 (213.31 to 145.2) | -1.02 (-1.55 to -0.49) |
| Saudi Arabia | 502 | 164.84 (168.67 to 161.08) | 707 | 121.95 (123.61 to 120.3) | -0.76 (-0.95 to -0.56) |
| Senegal | 5757 | 221.74 (229.09 to 214.57) | 10929 | 134.72 (138.36 to 131.16) | -1.37 (-1.57 to -1.18) |
| Serbia | 4023 | 131.72 (135.95 to 127.58) | 7658 | 41.5 (44.07 to 39.06) | -3.83 (-3.95 to -3.7) |
| Seychelles | 33797 | 256.83 (340.47 to 189.92) | 27685 | 127.04 (172.66 to 91.43) | -2.19 (-2.49 to -1.9) |
| Sierra Leone | 8599 | 202.74 (211.64 to 194.14) | 16847 | 209.65 (215.61 to 203.81) | 0.52 (0.24 to 0.79) |
| Singapore | 209 | 40.36 (44.18 to 36.8) | 553 | 14.44 (16.27 to 12.83) | -4.18 (-4.55 to -3.8) |
| Slovakia | 272 | 92.47 (97.14 to 87.97) | 1034 | 31.56 (34.46 to 28.88) | -3.36 (-3.54 to -3.17) |
| Slovenia | 971 | 50.19 (55.96 to 44.89) | 5260 | 9.58 (12.73 to 7.1) | -6.18 (-6.47 to -5.9) |
| Solomon Islands | 392 | 196.66 (232.54 to 165.18) | 735 | 243.25 (267.01 to 221.13) | 0.64 (0.57 to 0.72) |
| Somalia | 3070 | 209.43 (216.01 to 203) | 16428 | 157.95 (161.55 to 154.41) | -0.89 (-1.07 to -0.71) |
| South Africa | 1293 | 328.13 (331.68 to 324.62) | 3831 | 137.92 (139.55 to 136.3) | -3.36 (-4.38 to -2.34) |
| South Sudan | 190 | 144.72 (151.47 to 138.22) | 191 | 121.14 (125.94 to 116.47) | -0.74 (-1.15 to -0.33) |
| Spain | 1841 | 54.16 (55.57 to 52.79) | 5196 | 14.85 (15.6 to 14.13) | -5.15 (-5.48 to -4.82) |
| Sri Lanka | 2097 | 143.98 (147.25 to 140.77) | 4926 | 93.82 (96.25 to 91.44) | -1.76 (-2.09 to -1.42) |
| Sudan | 5733 | 357.54 (363.01 to 352.13) | 13153 | 170.83 (173.16 to 168.53) | -2.49 (-2.6 to -2.38) |
| Suriname | 12169 | 153.48 (180.45 to 129.73) | 18752 | 107.77 (124.83 to 92.53) | -1.37 (-1.72 to -1.03) |
| Sweden | 2400 | 21.79 (23.75 to 19.95) | 6210 | 6.99 (8.05 to 6.04) | -3.61 (-3.8 to -3.43) |
| Switzerland | 423 | 25.12 (27.29 to 23.08) | 1213 | 5.09 (6.07 to 4.24) | -6.07 (-6.47 to -5.68) |
| Syrian Arab Republic | 1043 | 348.72 (355.7 to 341.84) | 1102 | 158.26 (163.13 to 153.51) | -2.6 (-2.95 to -2.25) |
| Taiwan (Province of China) | 812 | 103.75 (106.12 to 101.41) | 1623 | 42.47 (44.04 to 40.94) | -2.7 (-3.09 to -2.31) |
| Tajikistan | 1184 | 156.06 (163.14 to 149.24) | 2791 | 84.32 (87.61 to 81.13) | -3.04 (-3.47 to -2.61) |
| Thailand | 2337 | 97.44 (98.91 to 95.99) | 5679 | 182.44 (184.43 to 180.47) | 1.19 (0.49 to 1.9) |
| Timor-Leste | 3402 | 212.73 (233.57 to 193.35) | 6546 | 219.95 (236.98 to 203.9) | 0.26 (-0.22 to 0.74) |
| Togo | 18765 | 226.2 (236.86 to 215.92) | 40789 | 154.97 (160.19 to 149.89) | -1.38 (-1.61 to -1.15) |
| Tokelau | 1860 | 289.84 (1547.9 to 10.7) | 3527 | 365.08 (1747.6 to 19.46) | 0.12 (-0.1 to 0.34) |
| Tonga | 53 | 136.2 (198.29 to 90.2) | 110 | 155.06 (211.45 to 110.79) | 0.49 (0.41 to 0.58) |
| Trinidad and Tobago | 3698 | 76.62 (86.26 to 67.83) | 5491 | 78.45 (87.19 to 70.45) | -0.27 (-0.58 to 0.05) |
| Tunisia | 48 | 92.41 (96.53 to 88.43) | 42 | 60.8 (63.36 to 58.32) | -1.52 (-1.58 to -1.47) |
| Turkey | 2126 | 155.47 (157.43 to 153.53) | 4994 | 46.77 (47.62 to 45.94) | -4.26 (-4.47 to -4.06) |
| Turkmenistan | 6 | 126.48 (133.79 to 119.48) | 2 | 203.14 (210.26 to 196.2) | 1.64 (1.11 to 2.17) |
| Tuvalu | 12 | 431.32 (764.59 to 219.31) | 7 | 476.52 (768.52 to 276.38) | 0.33 (0.21 to 0.45) |
| Uganda | 5 | 159.9 (164.16 to 155.73) | 3 | 109.97 (112.08 to 107.89) | -2.4 (-2.87 to -1.93) |
| Ukraine | 24 | 77.16 (78.53 to 75.81) | 7 | 108.27 (110.03 to 106.54) | 0.3 (-0.25 to 0.84) |
| United Arab Emirates | 49 | 87.71 (94.13 to 81.68) | 24 | 49.48 (52.63 to 46.51) | -1.81 (-2.12 to -1.5) |
| United Kingdom | 46 | 28.11 (28.93 to 27.3) | 68 | 12.63 (13.15 to 12.12) | -2.77 (-2.97 to -2.57) |
| United Republic of Tanzania | 21 | 151.03 (154.29 to 147.83) | 29 | 114.81 (116.57 to 113.07) | -1.19 (-1.35 to -1.03) |
| United States of America | 25 | 27.88 (28.24 to 27.53) | 33 | 24.58 (24.9 to 24.26) | -0.2 (-0.45 to 0.05) |
| United States Virgin Islands | 461 | 76.17 (114.53 to 48.27) | 197 | 62.41 (110.77 to 32.17) | -0.53 (-0.83 to -0.23) |
| Uruguay | 36 | 92.27 (98.97 to 85.92) | 10 | 37.71 (41.84 to 33.89) | -3.09 (-3.4 to -2.77) |
| Uzbekistan | 2 | 139.51 (142.67 to 136.41) | 1 | 89.92 (91.67 to 88.18) | -0.88 (-1.66 to -0.09) |
| Vanuatu | 12 | 513.83 (591.3 to 444.39) | 17 | 571.16 (624.62 to 521.28) | 0.13 (0.03 to 0.24) |
| Venezuela (Bolivarian Republic of) | 1 | 117.54 (120.46 to 114.67) | 1 | 67.96 (69.82 to 66.14) | -2.56 (-3.23 to -1.88) |
| Viet Nam | 23 | 186.24 (188.25 to 184.23) | 12 | 147.47 (148.77 to 146.19) | -0.21 (-0.52 to 0.11) |
| Yemen | 2 | 188.02 (193.09 to 183.06) | 1 | 116.95 (119.15 to 114.78) | -1.79 (-1.95 to -1.64) |
| Zambia | 1910 | 131.49 (137.3 to 125.87) | 2542 | 160.41 (163.96 to 156.93) | 0.81 (0.68 to 0.95) |
| Zimbabwe | 16977 | 42.69 (45.49 to 40.03) | 21256 | 125.92 (129.38 to 122.53) | 4.57 (3.3 to 5.85) |

**Supplementary Table S11. Disability-adjusted life years (DALYs) of subarachnoid hemorrhage attributable to metabolic risk factors among young adults in 1990 and 2021, and estimated annual percentage changes from 1990 to 2021, by country and territories.**

| **Countries** | Number of cases in 1990 | Age-standardized rate per 100000 population (95% UI) in 1990 | Number of cases in 2021 | Age-standardized rate per 100000 population (95% UI) in 2021 | Estimated annual percentage changes (95% CI) from 1990 to 2021 |
| --- | --- | --- | --- | --- | --- |
| Afghanistan | 1027 | 30.63 (33.72 to 27.78) | 692 | 27.49 (28.81 to 26.23) | -0.34 (-0.47 to -0.2) |
| Albania | 638 | 26.31 (29.72 to 23.21) | 831 | 20.95 (24.53 to 17.79) | -0.66 (-1.05 to -0.27) |
| Algeria | 117464 | 21.7 (22.91 to 20.53) | 67213 | 12 (12.58 to 11.44) | -2.33 (-2.55 to -2.12) |
| American Samoa | 29041 | 74.76 (138.21 to 36.32) | 37427 | 83.9 (153.49 to 40.8) | 0.16 (0.02 to 0.3) |
| Andorra | 2180 | 26.67 (60.9 to 9.49) | 2164 | 12.01 (39.52 to 2.25) | -2.58 (-2.71 to -2.44) |
| Angola | 1545 | 17.33 (19.05 to 15.73) | 2945 | 15.24 (16.15 to 14.38) | -0.43 (-0.59 to -0.27) |
| Antigua and Barbuda | 18 | 37.69 (78.97 to 15.05) | 42 | 19.21 (43.86 to 6.56) | -1.23 (-1.61 to -0.86) |
| Argentina | 5185 | 48.09 (49.54 to 46.67) | 8887 | 30.32 (31.25 to 29.41) | -1.13 (-1.37 to -0.88) |
| Armenia | 5782 | 23.26 (26.31 to 20.5) | 6807 | 15.24 (17.93 to 12.92) | -1.49 (-2 to -0.98) |
| Australia | 624 | 23.38 (24.71 to 22.11) | 892 | 12.19 (13.01 to 11.41) | -2.69 (-3.03 to -2.36) |
| Austria | 4542 | 31.21 (33.58 to 28.98) | 10982 | 11.89 (13.34 to 10.58) | -3.05 (-3.25 to -2.84) |
| Azerbaijan | 82 | 13.12 (14.76 to 11.62) | 137 | 8.19 (9.16 to 7.3) | -2.09 (-2.31 to -1.88) |
| Bahamas | 1121 | 36.37 (52.35 to 24.43) | 989 | 31.68 (43.54 to 22.4) | -0.75 (-0.94 to -0.56) |
| Bahrain | 11 | 15.32 (21.56 to 10.59) | 22 | 10.75 (13.71 to 8.32) | -1.76 (-2.05 to -1.47) |
| Bangladesh | 6007 | 73.7 (74.75 to 72.66) | 8731 | 64.63 (65.33 to 63.93) | -0.25 (-0.64 to 0.13) |
| Barbados | 189 | 37.01 (52.63 to 25.14) | 209 | 33.66 (48.95 to 22.23) | -0.15 (-0.41 to 0.12) |
| Belarus | 32 | 32.43 (34.44 to 30.51) | 36 | 36.08 (38.36 to 33.93) | 0.3 (0.02 to 0.58) |
| Belgium | 21 | 22.84 (24.6 to 21.19) | 48 | 13.24 (14.62 to 11.97) | -1.35 (-1.65 to -1.04) |
| Belize | 9 | 27.96 (48.93 to 14.73) | 13 | 20.55 (29.91 to 13.58) | -0.88 (-1.11 to -0.64) |
| Benin | 242 | 13.13 (15.51 to 11.04) | 1205 | 15.87 (17.32 to 14.51) | 0.69 (0.43 to 0.95) |
| Bermuda | 31 | 19.3 (49.63 to 5.61) | 52 | 9.94 (47.36 to 0.76) | -2.42 (-2.67 to -2.18) |
| Bhutan | 48 | 24.66 (34.13 to 17.35) | 117 | 17.41 (23.17 to 12.79) | -1.4 (-1.54 to -1.25) |
| Bolivia (Plurinational State of) | 28 | 53.45 (57.13 to 49.95) | 89 | 39.9 (41.99 to 37.88) | -1.12 (-1.41 to -0.82) |
| Bosnia and Herzegovina | 289 | 56.64 (60.6 to 52.87) | 310 | 27.14 (30.85 to 23.78) | -2.7 (-2.92 to -2.47) |
| Botswana | 815 | 13.73 (18.66 to 9.86) | 506 | 9.32 (11.6 to 7.39) | -1.41 (-1.62 to -1.19) |
| Brazil | 2424 | 80.48 (81.34 to 79.63) | 1863 | 40.26 (40.73 to 39.79) | -2.53 (-2.64 to -2.41) |
| Brunei Darussalam | 260 | 71.86 (91.52 to 55.59) | 153 | 44.22 (55.31 to 34.92) | -2 (-2.43 to -1.56) |
| Bulgaria | 467 | 48.29 (51.14 to 45.57) | 693 | 44.1 (47.36 to 41.03) | -0.32 (-0.46 to -0.17) |
| Burkina Faso | 390 | 11.95 (13.62 to 10.44) | 1049 | 11.3 (12.23 to 10.42) | -0.17 (-0.31 to -0.03) |
| Burundi | 212 | 31.98 (35.17 to 29.01) | 507 | 21.21 (22.79 to 19.71) | -1.5 (-1.75 to -1.25) |
| Cabo Verde | 766 | 30.28 (46.57 to 18.89) | 2330 | 16.88 (23.61 to 11.69) | -1.66 (-1.98 to -1.34) |
| Cambodia | 387 | 26.67 (28.84 to 24.62) | 742 | 14.89 (15.94 to 13.89) | -2.26 (-2.48 to -2.05) |
| Cameroon | 1177 | 12.29 (13.82 to 10.9) | 786 | 24.23 (25.3 to 23.19) | 2.29 (1.5 to 3.09) |
| Canada | 266 | 23.17 (24.16 to 22.21) | 156 | 13.46 (14.19 to 12.75) | -2.05 (-2.63 to -1.46) |
| Central African Republic | 705 | 21.81 (25.81 to 18.32) | 193 | 21.39 (24 to 19) | -0.22 (-0.32 to -0.12) |
| Chad | 842 | 16.71 (19.1 to 14.55) | 238 | 18.88 (20.37 to 17.48) | 0.38 (0.21 to 0.55) |
| Chile | 1372 | 38.75 (40.71 to 36.85) | 537 | 22.63 (23.89 to 21.42) | -1.61 (-1.85 to -1.38) |
| China | 45 | 30.95 (31.13 to 30.77) | 27 | 15.58 (15.7 to 15.47) | -2.82 (-3.19 to -2.46) |
| Colombia | 2006 | 30.04 (31.18 to 28.93) | 474 | 28.12 (28.97 to 27.29) | 0.52 (0.11 to 0.93) |
| Comoros | 7416 | 23.15 (34.95 to 14.65) | 3176 | 18.82 (25.57 to 13.51) | -1.16 (-1.64 to -0.68) |
| Congo | 474 | 20.47 (24.65 to 16.85) | 301 | 16.83 (18.99 to 14.86) | -1.04 (-1.26 to -0.82) |
| Cook Islands | 2697 | 66.82 (185.6 to 16.37) | 1632 | 44.33 (167.14 to 5.1) | -1.17 (-1.37 to -0.97) |
| Costa Rica | 1067 | 26.21 (29.83 to 22.95) | 1064 | 27.18 (29.9 to 24.66) | -0.05 (-0.43 to 0.33) |
| Côte d'Ivoire | 661 | 21.55 (23.31 to 19.9) | 326 | 21.03 (22.08 to 20.02) | 0.04 (-0.15 to 0.24) |
| Croatia | 2201 | 45.7 (49.21 to 42.38) | 877 | 17.36 (20.02 to 14.99) | -2.78 (-3.09 to -2.46) |
| Cuba | 172 | 28.75 (30.68 to 26.92) | 41 | 14.07 (15.49 to 12.76) | -2.22 (-2.51 to -1.94) |
| Cyprus | 188 | 33.5 (41.7 to 26.56) | 52 | 12.44 (16.17 to 9.49) | -3.95 (-4.39 to -3.5) |
| Czechia | 285 | 44.58 (47.03 to 42.24) | 184 | 19.4 (21.15 to 17.79) | -2.02 (-2.32 to -1.72) |
| Democratic People's Republic of Korea | 24259 | 37.41 (39.03 to 35.84) | 22748 | 25.73 (26.83 to 24.65) | -1.94 (-2.21 to -1.67) |
| Democratic Republic of the Congo | 268 | 16.94 (17.83 to 16.09) | 98 | 13.81 (14.3 to 13.33) | -0.85 (-0.99 to -0.71) |
| Denmark | 67 | 37.65 (40.87 to 34.63) | 77 | 13.45 (15.48 to 11.63) | -3.82 (-4.16 to -3.48) |
| Djibouti | 442 | 19.53 (30.43 to 11.89) | 144 | 19.01 (23.68 to 15.06) | -0.21 (-0.47 to 0.05) |
| Dominica | 14987 | 25.12 (61.81 to 7.65) | 6102 | 27.89 (62.52 to 9.75) | 0.36 (0.15 to 0.57) |
| Dominican Republic | 5366 | 35.27 (38.05 to 32.65) | 6555 | 33.69 (35.68 to 31.78) | 0.66 (0.35 to 0.97) |
| Ecuador | 346 | 27.79 (29.88 to 25.82) | 224 | 38.38 (40.06 to 36.77) | 1.88 (1.53 to 2.23) |
| Egypt | 8229 | 24.54 (25.36 to 23.74) | 1660 | 17.23 (17.69 to 16.77) | -0.69 (-0.96 to -0.42) |
| El Salvador | 154 | 48.69 (52.67 to 44.94) | 106 | 30.05 (32.68 to 27.6) | -1.36 (-1.97 to -0.74) |
| Equatorial Guinea | 1270 | 20.22 (31.74 to 12.14) | 920 | 11.42 (15.11 to 8.47) | -2.51 (-2.94 to -2.08) |
| Eritrea | 6 | 23.7 (27.42 to 20.37) | 3 | 21.44 (23.64 to 19.41) | -0.26 (-0.39 to -0.14) |
| Estonia | 573 | 36.21 (42.07 to 31) | 195 | 11.14 (15.32 to 7.99) | -4.54 (-4.92 to -4.16) |
| Eswatini | 80 | 10.4 (16.64 to 6.1) | 61 | 13.8 (18.28 to 10.2) | 1.32 (0.68 to 1.97) |
| Ethiopia | 729 | 7.39 (7.92 to 6.89) | 295 | 10.87 (11.27 to 10.49) | 1.38 (1.21 to 1.54) |
| Fiji | 1219 | 81.8 (94.48 to 70.48) | 348 | 75.06 (85.99 to 65.22) | -0.45 (-0.57 to -0.32) |
| Finland | 590 | 76 (80.41 to 71.79) | 338 | 24.03 (26.72 to 21.57) | -3.61 (-3.88 to -3.34) |
| France | 705 | 24.9 (25.64 to 24.17) | 398 | 15.45 (16.07 to 14.85) | -1.58 (-1.77 to -1.39) |
| Gabon | 16 | 13.34 (18.84 to 9.14) | 13 | 11.99 (15.44 to 9.15) | -0.5 (-0.63 to -0.37) |
| Gambia | 4432 | 20.22 (27.09 to 14.79) | 2494 | 26.16 (30.5 to 22.31) | 0.6 (0.33 to 0.87) |
| Georgia | 457 | 48.29 (51.73 to 45.02) | 206 | 49.61 (54.17 to 45.37) | 0.05 (-0.73 to 0.84) |
| Germany | 12220 | 49 (49.88 to 48.14) | 2818 | 12.5 (12.97 to 12.04) | -4.42 (-4.72 to -4.13) |
| Ghana | 284 | 35.13 (37.11 to 33.24) | 183 | 24.47 (25.47 to 23.5) | -1.17 (-1.36 to -0.98) |
| Greece | 24 | 20.21 (21.91 to 18.61) | 13 | 13.64 (15.2 to 12.21) | -1.32 (-1.45 to -1.18) |
| Greenland | 3633 | 104.32 (159.65 to 64.91) | 1160 | 36.51 (79.81 to 13.7) | -3.78 (-3.99 to -3.56) |
| Grenada | 568 | 52.27 (93.4 to 26.49) | 160 | 31.64 (60.41 to 14.53) | -1.45 (-1.69 to -1.2) |
| Guam | 1554 | 37.32 (59.26 to 22.16) | 451 | 51.73 (78.57 to 32.39) | 1.05 (0.82 to 1.28) |
| Guatemala | 40 | 12.39 (14.11 to 10.84) | 15 | 32.73 (34.44 to 31.08) | 3.91 (3.37 to 4.46) |
| Guinea | 555 | 18.06 (20.45 to 15.89) | 143 | 23.14 (24.84 to 21.52) | 1.16 (0.99 to 1.34) |
| Guinea-Bissau | 1243 | 32.85 (41.29 to 25.8) | 387 | 34.09 (39.34 to 29.41) | 0.23 (0.13 to 0.33) |
| Guyana | 2112 | 40.78 (50.26 to 32.75) | 1323 | 50.37 (60.91 to 41.3) | 0.52 (0.18 to 0.87) |
| Haiti | 1588 | 99.79 (104.77 to 94.99) | 1320 | 104.58 (107.77 to 101.47) | 0.58 (0.46 to 0.7) |
| Honduras | 490 | 66.67 (71.75 to 61.86) | 144 | 42.89 (45.33 to 40.55) | -2.02 (-2.35 to -1.68) |
| Hungary | 788 | 62.04 (64.84 to 59.33) | 424 | 19.5 (21.35 to 17.78) | -4.06 (-4.21 to -3.9) |
| Iceland | 8638 | 19.71 (32.06 to 11.27) | 2927 | 12.79 (22.14 to 6.77) | -0.7 (-0.93 to -0.47) |
| India | 4351 | 28.87 (29.09 to 28.65) | 4213 | 19.39 (19.52 to 19.26) | -1.31 (-1.63 to -0.99) |
| Indonesia | 2211 | 55.87 (56.52 to 55.22) | 1370 | 41.43 (41.85 to 41.01) | -0.8 (-0.96 to -0.63) |
| Iran (Islamic Republic of) | 21518 | 9.24 (9.78 to 8.73) | 16670 | 7.08 (7.38 to 6.78) | -0.72 (-0.9 to -0.55) |
| Iraq | 7 | 29.8 (31.49 to 28.18) | 5 | 16.5 (17.24 to 15.78) | -2.34 (-2.65 to -2.03) |
| Ireland | 936 | 45.31 (49.67 to 41.25) | 422 | 15.52 (17.83 to 13.45) | -3.59 (-3.96 to -3.21) |
| Israel | 30 | 20.17 (22.66 to 17.89) | 38 | 7.19 (8.31 to 6.19) | -3.18 (-3.37 to -2.99) |
| Italy | 5 | 22.31 (23.05 to 21.59) | 6 | 8.47 (8.98 to 7.99) | -3.06 (-3.27 to -2.85) |
| Jamaica | 686 | 32.69 (37.56 to 28.32) | 1166 | 28.88 (32.6 to 25.5) | -0.33 (-0.8 to 0.14) |
| Japan | 13 | 42.21 (42.89 to 41.53) | 28 | 21.2 (21.74 to 20.66) | -2.58 (-2.81 to -2.36) |
| Jordan | 1629 | 9.13 (11.5 to 7.16) | 4280 | 6.06 (6.9 to 5.31) | -1.69 (-1.83 to -1.55) |
| Kazakhstan | 31 | 46.48 (48.38 to 44.64) | 27 | 30.61 (32.04 to 29.23) | -1.36 (-1.95 to -0.76) |
| Kenya | 206 | 11.76 (12.77 to 10.82) | 265 | 12.74 (13.35 to 12.16) | 0.46 (0.23 to 0.7) |
| Kiribati | 12 | 96.04 (149.51 to 58.63) | 9 | 128.68 (171.04 to 94.78) | 0.81 (0.67 to 0.95) |
| Kuwait | 91 | 11.21 (13.97 to 8.88) | 110 | 6.01 (7.23 to 5) | -2.5 (-2.91 to -2.08) |
| Kyrgyzstan | 8 | 37.52 (41.12 to 34.16) | 8 | 33.02 (35.59 to 30.6) | -1.5 (-1.95 to -1.05) |
| Lao People's Democratic Republic | 37 | 63.42 (68.65 to 58.5) | 50 | 37.36 (39.9 to 34.94) | -1.87 (-1.98 to -1.77) |
| Latvia | 15 | 34.44 (38.84 to 30.44) | 14 | 19.04 (23.39 to 15.43) | -2.4 (-3.03 to -1.77) |
| Lebanon | 874 | 35.06 (39.54 to 30.99) | 1479 | 16.42 (18.3 to 14.7) | -2.06 (-2.64 to -1.47) |
| Lesotho | 145 | 8.19 (11.83 to 5.45) | 252 | 16.95 (20.77 to 13.68) | 3.3 (2.77 to 3.83) |
| Liberia | 749 | 21.31 (25.3 to 17.82) | 2128 | 24.9 (27.56 to 22.44) | 0.81 (0.64 to 0.99) |
| Libya | 236 | 18.93 (21.85 to 16.32) | 425 | 21 (22.89 to 19.23) | 0.34 (0.13 to 0.55) |
| Lithuania | 1576 | 40.55 (44.52 to 36.85) | 4446 | 20.47 (24.13 to 17.26) | -1.6 (-2.42 to -0.77) |
| Luxembourg | 636 | 30.83 (42.18 to 21.98) | 561 | 7.34 (12.4 to 4.09) | -5 (-5.23 to -4.78) |
| Madagascar | 2791 | 42.56 (45.07 to 40.17) | 4378 | 44.43 (45.98 to 42.91) | 0.21 (0.15 to 0.26) |
| Malawi | 5969 | 21.08 (23.11 to 19.19) | 12119 | 22.03 (23.4 to 20.73) | -0.08 (-0.42 to 0.27) |
| Malaysia | 232 | 29.59 (31.11 to 28.13) | 1503 | 26.48 (27.46 to 25.53) | 0.24 (-0.16 to 0.63) |
| Maldives | 722 | 39.26 (63.45 to 22.9) | 1272 | 15.9 (22.01 to 11.43) | -3.01 (-3.25 to -2.77) |
| Mali | 188 | 16.11 (18.03 to 14.35) | 409 | 14.46 (15.55 to 13.43) | -0.29 (-0.53 to -0.04) |
| Malta | 330 | 20.43 (30.73 to 13.06) | 586 | 10.62 (18.61 to 5.68) | -1.5 (-1.71 to -1.3) |
| Marshall Islands | 631 | 97.53 (175.78 to 48.88) | 833 | 121.54 (185.19 to 75.77) | 0.71 (0.63 to 0.79) |
| Mauritania | 3378 | 23.19 (27.89 to 19.12) | 3419 | 15.64 (18.2 to 13.36) | -1.27 (-1.4 to -1.14) |
| Mauritius | 3613 | 46.37 (53.88 to 39.68) | 5446 | 60.41 (68.98 to 52.67) | 2.58 (1.99 to 3.17) |
| Mexico | 1234 | 26.37 (27.05 to 25.69) | 2282 | 30.53 (31.08 to 29.99) | 0.6 (0.4 to 0.8) |
| Micronesia (Federated States of) | 1323 | 117.81 (167.48 to 80.18) | 1750 | 120.1 (167.08 to 83.89) | 0.05 (0.01 to 0.09) |
| Monaco | 34420 | 41.02 (117.22 to 9.39) | 28597 | 20.93 (88.37 to 1.78) | -2.17 (-2.25 to -2.09) |
| Mongolia | 34 | 39.85 (45.79 to 34.54) | 67 | 46.69 (50.96 to 42.71) | 0.56 (0.15 to 0.97) |
| Montenegro | 272 | 23.27 (31.11 to 17) | 336 | 15.35 (22.5 to 10.07) | -1.55 (-1.94 to -1.16) |
| Morocco | 1293 | 33.94 (35.36 to 32.55) | 2028 | 17.68 (18.47 to 16.92) | -2.42 (-2.64 to -2.2) |
| Mozambique | 77 | 23.17 (24.95 to 21.49) | 234 | 37.78 (39.27 to 36.35) | 2.13 (1.89 to 2.37) |
| Myanmar | 191 | 51.3 (52.65 to 49.97) | 521 | 41.23 (42.23 to 40.26) | -0.33 (-0.65 to -0.02) |
| Namibia | 2298 | 10.22 (14.42 to 7.02) | 2030 | 10.65 (13.31 to 8.41) | -0.22 (-0.62 to 0.17) |
| Nauru | 80 | 180.5 (420.66 to 60.34) | 128 | 217.67 (448.24 to 89.13) | 0.36 (-0.24 to 0.96) |
| Nepal | 38 | 35.25 (36.98 to 33.57) | 95 | 17.3 (18.18 to 16.45) | -2.53 (-2.71 to -2.34) |
| Netherlands | 635 | 32.13 (33.77 to 30.55) | 1928 | 10.52 (11.53 to 9.57) | -3.49 (-3.69 to -3.29) |
| New Zealand | 826 | 32.45 (36.06 to 29.12) | 558 | 15.14 (17.27 to 13.22) | -2.74 (-2.96 to -2.52) |
| Nicaragua | 80 | 37.09 (41.38 to 33.16) | 245 | 27.44 (29.76 to 25.26) | -0.64 (-0.85 to -0.44) |
| Niger | 354 | 15.34 (17.35 to 13.52) | 434 | 14.88 (16.02 to 13.81) | -0.03 (-0.12 to 0.06) |
| Nigeria | 602 | 9.96 (10.4 to 9.53) | 1666 | 11.39 (11.68 to 11.1) | 0.66 (0.41 to 0.91) |
| Niue | 61 | 93.5 (868.25 to 0.12) | 203 | 90.86 (1110.57 to 0.01) | -0.91 (-1.15 to -0.68) |
| North Macedonia | 8762 | 77.62 (84.95 to 70.79) | 4935 | 43 (48.21 to 38.26) | -1.64 (-1.96 to -1.32) |
| Northern Mariana Islands | 38 | 67.14 (115.64 to 35.55) | 47 | 66.2 (129.99 to 29.29) | -0.26 (-0.49 to -0.03) |
| Norway | 451 | 44.64 (48.47 to 41.04) | 1896 | 10.67 (12.47 to 9.09) | -4.63 (-4.86 to -4.41) |
| Oman | 67229 | 11.88 (14.83 to 9.4) | 87925 | 10.68 (12.17 to 9.37) | 0.28 (0.05 to 0.51) |
| Pakistan | 181 | 26.7 (27.36 to 26.06) | 549 | 40.29 (40.76 to 39.81) | 1.32 (1.17 to 1.47) |
| Palau | 1680 | 84.87 (213.32 to 24.77) | 1563 | 117.32 (265.07 to 41.35) | 1.24 (1.09 to 1.38) |
| Palestine | 436 | 8.85 (12.29 to 6.22) | 1168 | 6.37 (7.83 to 5.14) | -1.13 (-1.37 to -0.88) |
| Panama | 6548 | 27.79 (32.11 to 23.93) | 27703 | 33.23 (36.62 to 30.09) | 0.81 (0.48 to 1.15) |
| Papua New Guinea | 141 | 22.63 (25.72 to 19.83) | 294 | 39.18 (41.46 to 36.98) | 1.89 (1.44 to 2.34) |
| Paraguay | 19588 | 59.92 (64.81 to 55.32) | 33009 | 36.2 (38.75 to 33.78) | -1.37 (-1.5 to -1.24) |
| Peru | 114 | 26.94 (28.32 to 25.62) | 265 | 38.14 (39.28 to 37.02) | 2.02 (1.54 to 2.5) |
| Philippines | 33 | 27.08 (27.88 to 26.29) | 61 | 32.22 (32.83 to 31.62) | 1.21 (0.92 to 1.49) |
| Poland | 1499 | 57.73 (59.07 to 56.42) | 3146 | 26.8 (27.76 to 25.87) | -2.14 (-2.3 to -1.97) |
| Portugal | 19 | 43.83 (46.33 to 41.42) | 51 | 15.3 (16.92 to 13.8) | -3.77 (-4.14 to -3.4) |
| Puerto Rico | 23 | 27.98 (31.39 to 24.86) | 41 | 17.74 (20.87 to 14.98) | -1.84 (-2.1 to -1.58) |
| Qatar | 184 | 26.67 (34.66 to 20.36) | 410 | 11.39 (13.21 to 9.83) | -3.19 (-3.55 to -2.84) |
| Republic of Korea | 20 | 54.13 (55.32 to 52.96) | 80 | 11.42 (11.99 to 10.88) | -6.15 (-6.71 to -5.58) |
| Republic of Moldova | 435 | 19.23 (21.61 to 17.06) | 745 | 14.87 (17.28 to 12.78) | -0.98 (-1.54 to -0.41) |
| Romania | 590 | 39.83 (41.38 to 38.34) | 1794 | 34.57 (36.3 to 32.9) | -0.88 (-1.42 to -0.33) |
| Russian Federation | 825 | 47.68 (48.28 to 47.08) | 3118 | 49.09 (49.74 to 48.44) | -0.34 (-1 to 0.33) |
| Rwanda | 468 | 34.39 (37.26 to 31.7) | 1075 | 14.77 (16.04 to 13.57) | -3.72 (-4.15 to -3.29) |
| Saint Kitts and Nevis | 610 | 70.09 (139.1 to 30.57) | 567 | 28.86 (65.86 to 9.99) | -3.89 (-4.61 to -3.17) |
| Saint Lucia | 704 | 44.28 (73.96 to 24.63) | 2664 | 26.42 (44.19 to 14.57) | -1.78 (-2.07 to -1.48) |
| Saint Vincent and the Grenadines | 693 | 27.62 (56.63 to 11.57) | 1520 | 26.41 (51.57 to 11.71) | -0.73 (-1.03 to -0.44) |
| Samoa | 1204 | 75.25 (108.42 to 50.57) | 3304 | 92.96 (122.42 to 69.28) | 0.6 (0.46 to 0.73) |
| San Marino | 172 | 16.47 (84.08 to 0.65) | 219 | 7.62 (70 to 0.01) | -2.25 (-2.49 to -2.01) |
| Sao Tome and Principe | 8 | 25.67 (57.29 to 9.36) | 7 | 28.48 (45.3 to 16.8) | 0.25 (-0.22 to 0.73) |
| Saudi Arabia | 445 | 14.2 (15.36 to 13.1) | 1070 | 11.03 (11.54 to 10.54) | -0.75 (-0.94 to -0.55) |
| Senegal | 204 | 24.21 (26.72 to 21.89) | 1065 | 19.12 (20.52 to 17.8) | -0.51 (-0.78 to -0.24) |
| Serbia | 33 | 75.52 (78.74 to 72.4) | 78 | 33.8 (36.13 to 31.59) | -2.5 (-2.63 to -2.37) |
| Seychelles | 1029 | 41.56 (82.88 to 18.05) | 2548 | 22.15 (46.13 to 9.05) | -1.82 (-2.05 to -1.59) |
| Sierra Leone | 42 | 21.61 (24.65 to 18.88) | 80 | 26.03 (28.18 to 24.01) | 0.99 (0.73 to 1.24) |
| Singapore | 2040 | 13.14 (15.4 to 11.14) | 2575 | 5.19 (6.35 to 4.24) | -4.01 (-4.35 to -3.67) |
| Slovakia | 18 | 38.66 (41.74 to 35.77) | 49 | 20.13 (22.49 to 17.99) | -1.82 (-1.94 to -1.69) |
| Slovenia | 28 | 29.03 (33.51 to 25.02) | 94 | 9.77 (12.94 to 7.28) | -3.64 (-3.89 to -3.4) |
| Solomon Islands | 229 | 38.69 (56.25 to 25.64) | 619 | 48 (59.12 to 38.53) | 0.7 (0.58 to 0.81) |
| Somalia | 271 | 23.11 (25.36 to 21.01) | 1362 | 21.91 (23.28 to 20.61) | -0.07 (-0.23 to 0.08) |
| South Africa | 288 | 19.8 (20.69 to 18.94) | 2066 | 12.83 (13.34 to 12.34) | -1.63 (-2.39 to -0.87) |
| South Sudan | 142 | 14.5 (16.73 to 12.5) | 517 | 15.02 (16.77 to 13.4) | 0 (-0.32 to 0.33) |
| Spain | 23 | 19.3 (20.14 to 18.48) | 34 | 12.06 (12.73 to 11.41) | -2.27 (-2.53 to -2.01) |
| Sri Lanka | 48 | 20.98 (22.25 to 19.77) | 167 | 15.72 (16.73 to 14.75) | -0.95 (-1.11 to -0.79) |
| Sudan | 218 | 40.46 (42.34 to 38.65) | 689 | 25.66 (26.58 to 24.77) | -1.59 (-1.66 to -1.53) |
| Suriname | 1301 | 36.23 (50.45 to 25.32) | 2436 | 30.05 (39.67 to 22.28) | -0.86 (-1.14 to -0.57) |
| Sweden | 252 | 23.21 (25.23 to 21.32) | 774 | 5.14 (6.07 to 4.33) | -4.62 (-4.8 to -4.44) |
| Switzerland | 307 | 21.98 (24.02 to 20.07) | 750 | 5.74 (6.77 to 4.84) | -5.1 (-5.43 to -4.77) |
| Syrian Arab Republic | 647 | 29 (31.09 to 27.03) | 1645 | 18.11 (19.8 to 16.54) | -1.6 (-1.86 to -1.33) |
| Taiwan (Province of China) | 75 | 14.12 (15.01 to 13.27) | 192 | 10.04 (10.82 to 9.3) | -1.82 (-2.59 to -1.06) |
| Tajikistan | 115 | 29.19 (32.32 to 26.29) | 171 | 23.42 (25.18 to 21.76) | -1.34 (-1.59 to -1.09) |
| Thailand | 133 | 28.85 (29.65 to 28.07) | 377 | 49.2 (50.24 to 48.18) | 1.29 (0.81 to 1.77) |
| Timor-Leste | 2100 | 38.16 (47.53 to 30.28) | 6237 | 40.92 (48.58 to 34.24) | 0.44 (0.02 to 0.86) |
| Togo | 263 | 24.37 (28.03 to 21.09) | 734 | 20.03 (21.96 to 18.23) | -0.79 (-1.04 to -0.55) |
| Tokelau | 6 | 84.63 (1188.56 to 0) | 18 | 104.2 (1292.49 to 0.01) | 0.04 (-0.17 to 0.25) |
| Tonga | 203 | 41.52 (81.06 to 18.7) | 459 | 47.95 (83.09 to 25.28) | 0.56 (0.49 to 0.62) |
| Trinidad and Tobago | 11 | 39.39 (46.44 to 33.2) | 10 | 56.78 (64.4 to 49.92) | 1.11 (0.85 to 1.37) |
| Tunisia | 408 | 15.99 (17.77 to 14.35) | 789 | 11.54 (12.69 to 10.48) | -1.14 (-1.18 to -1.1) |
| Turkey | 4 | 55.29 (56.47 to 54.14) | 2 | 19.12 (19.66 to 18.59) | -3.74 (-3.87 to -3.61) |
| Turkmenistan | 228 | 38.99 (43.13 to 35.17) | 626 | 65.43 (69.53 to 61.53) | 2.02 (1.64 to 2.41) |
| Tuvalu | 18 | 108.95 (325.97 to 21.99) | 22 | 129.02 (315.7 to 39.58) | 0.56 (0.42 to 0.69) |
| Uganda | 3 | 18.96 (20.46 to 17.55) | 2 | 15.05 (15.84 to 14.29) | -1.67 (-2.02 to -1.33) |
| Ukraine | 5 | 33.48 (34.39 to 32.59) | 7 | 47.36 (48.55 to 46.21) | 1.22 (0.97 to 1.47) |
| United Arab Emirates | 1 | 20.1 (23.33 to 17.27) | 0 | 12.73 (14.32 to 11.31) | -1.43 (-1.71 to -1.15) |
| United Kingdom | 1 | 52.65 (53.78 to 51.55) | 1 | 15.75 (16.34 to 15.19) | -4.13 (-4.33 to -3.93) |
| United Republic of Tanzania | 22 | 17.88 (19.02 to 16.79) | 6 | 17.21 (17.89 to 16.54) | -0.35 (-0.51 to -0.18) |
| United States of America | 13 | 25.05 (25.39 to 24.72) | 8 | 18.37 (18.65 to 18.09) | -0.94 (-1.22 to -0.66) |
| United States Virgin Islands | 4 | 44.02 (75.14 to 23.6) | 6 | 32.8 (72.12 to 12.33) | -0.8 (-1.09 to -0.5) |
| Uruguay | 292 | 92.44 (99.13 to 86.09) | 146 | 44.89 (49.39 to 40.72) | -2.43 (-2.8 to -2.06) |
| Uzbekistan | 4 | 13.52 (14.52 to 12.57) | 2 | 20.4 (21.25 to 19.58) | 2.31 (1.37 to 3.26) |
| Vanuatu | 0 | 121.77 (162.01 to 89.67) | 0 | 133.59 (160.45 to 110.32) | 0.07 (-0.03 to 0.18) |
| Venezuela (Bolivarian Republic of) | 3 | 61.15 (63.26 to 59.1) | 5 | 44.35 (45.87 to 42.87) | -1.57 (-2.13 to -1) |
| Viet Nam | 194 | 32.39 (33.23 to 31.57) | 315 | 25.67 (26.22 to 25.14) | -0.15 (-0.47 to 0.17) |
| Yemen | 13 | 20.74 (22.47 to 19.11) | 6 | 17.6 (18.47 to 16.76) | -0.82 (-0.98 to -0.66) |
| Zambia | 8 | 12.52 (14.4 to 10.84) | 5 | 20.66 (21.95 to 19.43) | 1.98 (1.8 to 2.15) |
| Zimbabwe | 1901 | 11.86 (13.37 to 10.48) | 3174 | 32.51 (34.3 to 30.81) | 4.1 (3.1 to 5.1) |
